# Crosstalk between Plk1, p53, cell cycle, and G2/M DNA damage checkpoint regulation in cancer: computational modeling and analysis

**DOI:** 10.1038/s41540-021-00203-8

**Published:** 2021-12-09

**Authors:** Yongwoon Jung, Pavel Kraikivski, Sajad Shafiekhani, Scott S. Terhune, Ranjan K. Dash

**Affiliations:** 1 Department of Biomedical Engineering, Medical College of Wisconsin, Milwaukee, WI 53226 USA; 2 Academy of Integrated Science, Division of Systems Biology, Virginia Tech, Blacksburg, VA 24061 USA; 3 Department of Biomedical Engineering, School of Medicine, Tehran University of Medical Sciences, Tehran, Iran; 4 Departments of Microbiology and Immunology, Medical College of Wisconsin, Milwaukee, WI 53226 USA; 5 Center of Systems and Molecular Medicine, Medical College of Wisconsin, Milwaukee, WI 53226 USA; 6 Department of Physiology, Medical College of Wisconsin, Milwaukee, WI 53226 USA

**Keywords:** Regulatory networks, Cancer

## Abstract

Different cancer cell lines can have varying responses to the same perturbations or stressful conditions. Cancer cells that have DNA damage checkpoint-related mutations are often more sensitive to gene perturbations including altered Plk1 and p53 activities than cancer cells without these mutations. The perturbations often induce a cell cycle arrest in the former cancer, whereas they only delay the cell cycle progression in the latter cancer. To study crosstalk between Plk1, p53, and G2/M DNA damage checkpoint leading to differential cell cycle regulations, we developed a computational model by extending our recently developed model of mitotic cell cycle and including these key interactions. We have used the model to analyze the cancer cell cycle progression under various gene perturbations including Plk1-depletion conditions. We also analyzed mutations and perturbations in approximately 1800 different cell lines available in the Cancer Dependency Map and grouped lines by genes that are represented in our model. Our model successfully explained phenotypes of various cancer cell lines under different gene perturbations. Several sensitivity analysis approaches were used to identify the range of key parameter values that lead to the cell cycle arrest in cancer cells. Our resulting model can be used to predict the effect of potential treatments targeting key mitotic and DNA damage checkpoint regulators on cell cycle progression of different types of cancer cells.

## Introduction

The G2/M DNA damage checkpoint is an essential control mechanism in cell cycle regulation. It ensures that all damaged DNA is repaired before cells are permitted to continue cycle progression^1^. Cell cycle regulators that are involved in this checkpoint are often found to have abnormal expression in cancer cells^2^. Particularly, polo-like kinase (PLK1) gene expression is elevated in proliferating cells of tumors of various origin^3^. Another gene that often has abnormal expression in human cancers is TP53 which encodes the tumor suppresser protein p53^4^. It has been demonstrated that both Plk1 and p53 interactions play a critical role in regulating the DNA damage checkpoint^5^. Remarkably, Liu and co-workers^5^ have shown that depletion of Plk1 in p53-null cancer cells promotes the activation of DNA damage checkpoint and induces G2/M arrest and apoptosis. Furthermore, cancer cells with functional p53 are much less sensitive to Plk1 depletion than p53-deficient cancer cells^6,7^. Cancers with functional p53 have normal or slowed proliferation rates with no cell cycle arrest, whereas p53-deficient cancer cells show G2/M arrest^6,7^. Yim and Erikson^8^ have determined that Plk1 depletion induces DNA damage in both S and G2/M cell cycle phases with the effect in G2/M being more pronounced. They demonstrated that DNA damage occurs at the first S phase following Plk1 depletion and the response is more severe in Plk1-depleted p53-null cancer cells. Therefore, p53 interactions might be involved in Plk1-depletion-induced G2/M arrest in cancer cells. However, the exact mechanism that is responsible for this arrest and associated apoptosis in Plk1-depleted cancer cells remains unknown.

To uncover the mechanism of Plk1-depletion-induced G2/M arrest in cancer cells and the critical role of p53, we developed a computational model by extending our recently developed model of mitotic cell cycle^9^ and incorporating the crosstalk of Plk1, p53, G2/M DNA damage checkpoint, and associated regulators. We then used the model to investigate the consequences of altered molecular interactions between Plk1 and p53 pathways as well as between other components that are involved in the checkpoint and cell cycle control mechanisms. The model entails the use of ordinary differential equations (ODEs) based on mass balance principle that incorporate mechanistic kinetic fluxes involving molecular interactions of G2/M DNA damage checkpoint regulation and cell cycle progression. An ODE approach is extensively used to study the dynamic behavior of complex biochemical regulatory networks^10–13^, and has been effective to study both cell cycle^9,14–19^ and checkpoint regulations^18–25^.

The ODE approach to study the dynamics of biological systems requires the determination of numerical values of the associated kinetic constants (adjustable model parameters) based on available experimental data^9,11^. The parameter values are determined as detailed in ref. ^9^ and are justified by performing a sensitivity analysis. The corroboration and validation of the model was performed by using CRISPR perturbation data publicly available from the Cancer Dependency Map database as well as perturbation data gathered from literature. We analyzed mutations in 1749 different cancer cell lines and perturbations of genes in 808 cell lines to obtain a dataset for the model corroboration and validation. We also used several different sensitivity analysis methods to quantify dependencies of protein concentrations on parameter variations and to identify key G2/M checkpoint regulation components whose perturbations can cause cell cycle arrest in different cancer cell lines. This information can be used to predict the effect of a potential treatment on the cell cycle of cancer cells.

## Results

### G2/M DNA damage checkpoint regulation mechanism

**Fig. 1:**
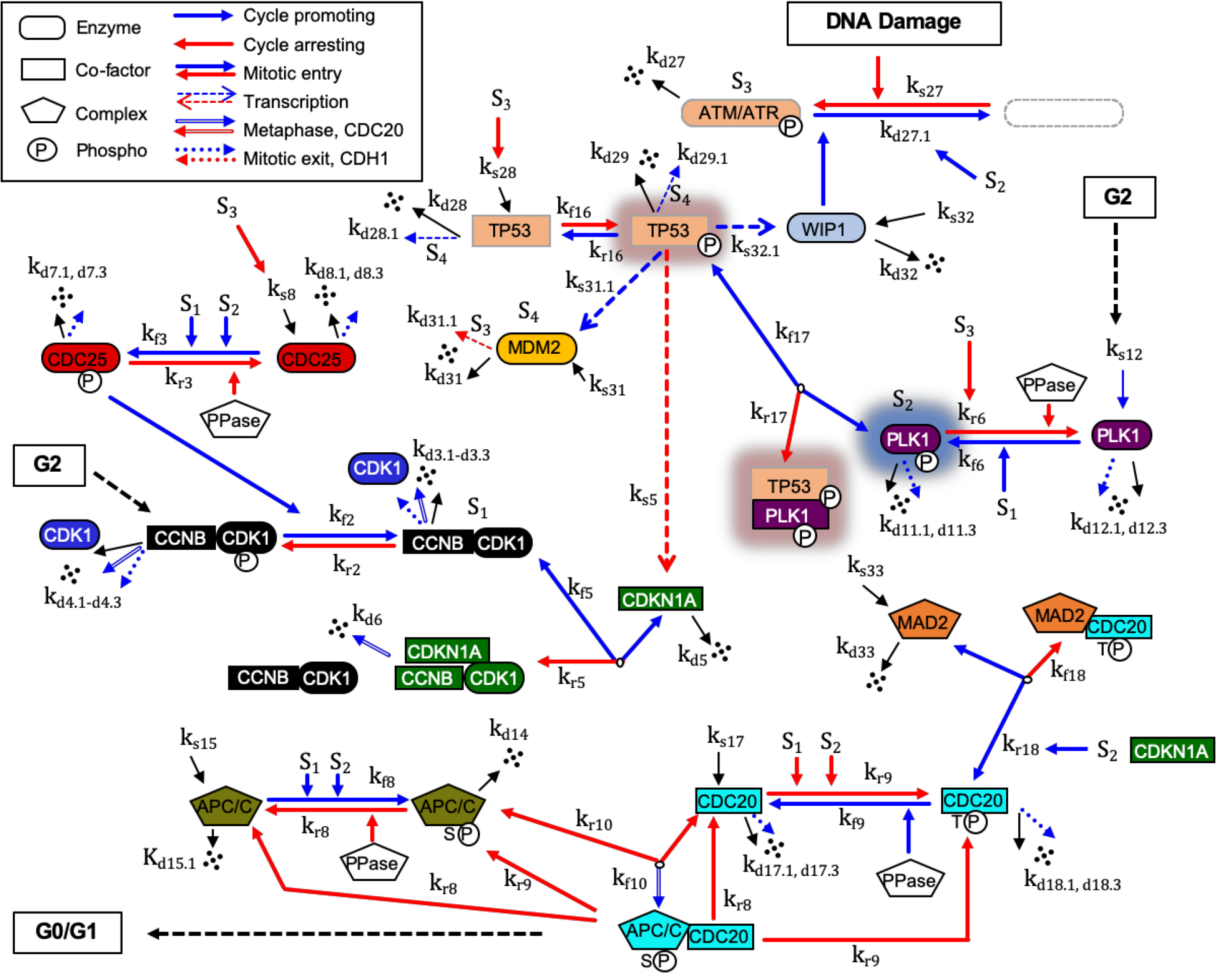
G2/M DNA damage checkpoint regulatory network. The G2/M DNA damage biopathway for the reaction network of key proteins is converted into a mathematical structure based on ODEs involving the law of mass conservations and a hybrid framework combining mass action and Michaelis-Menten kinetics. Each factor, as defined in the text and Supplementary information, is identified by a unique color. Gene symbols have been used to be consistent with our previous model of the mitotic cell cycle regulation^9^. Here, S1 denotes active MPF kinase (CCNB:CDK1), S2 denotes active PLK1 kinase (phosphorylated), S3 denotes activation of ATM or ATR-induced DNA damage response, and S4 denotes activities of the E3 ubiquitin ligase, MDM2. PPase represents generic phosphatases. The model consists of 34 ODEs governing 22 interaction reactions (*k*_fn_ denotes the forward rate constant and *k*_rn_ denotes the reverse rate constant for reaction *n*) of 50 key mitotic and DNA damage checkpoint regulation proteins and associated protein complexes and includes 15 synthesis reactions (*k*_s_: synthesis rate constant) and 26 reactions of multiple degradations (*k*_dn_: self-degradation rate constant, *k*_dn.n_: degradation rate constant by other factors including APP/CP:CDC20 and APC/CT:CDH1). Synthesis *k*_s_ represents either expression of a protein factor or accumulation of a specific post translationally modified factor. The crosstalk components in TP53 and PLK1 pathways are highlighted using background shadows.

To build the computational model of molecular mechanism of the G2/M DNA damage checkpoint regulation in cancer cells, we gathered relevant molecular interaction data from the published literature. The resulting G2/M DNA damage checkpoint regulatory network is shown in Fig. 1. This regulatory network was then integrated into our recently published regulatory network of the mitotic cell cycle^9^ resulting in an enhanced network describing interactions between 34 dynamic components. The integration of the DNA damage checkpoint regulation mechanism with the mitotic cell cycle regulation allowed us to computationally model and quantitatively characterize the phenotypes of perturbed cancer cells that often have mutations directly affecting genes involved in the DNA damage checkpoint and the cell cycle regulation.

In mammalian cells, the ATM (ataxia-telangiectasia mutated) and ATR (ATM- and Rad3-related) kinases are one of the upstream DNA damage response (DDR) signaling components^1,26^. As shown in Fig. 1, ATM/ATR checkpoint activation (signal S3) downregulates M-phase inducer phosphatase Cdc25^27^, activates tumor suppresser protein p53^28^, and inhibits Plk1 activity^29^. p53 is a transcription factor that induces the expression of its inhibitors Mdm2 and Wip1. Wip1 phosphatase dephosphorylates p53 protein and inhibits activity of ATM and ATR kinases^30,31^. Mdm2 (signal S4) is a p53-specific E3 ubiquitin ligase that promotes p53 degradation^32^. These regulations of p53 through negative feedback loops (p53 inducing the expression of Wip1 which dephosphorylates p53, and p53 inducing the expression of Mdm2 resulting ubiquitination of p53 and degradation) are known to induce oscillatory behavior of p53 activity^32–35^. Our computational model was successful in explaining these oscillations of DNA damage checkpoint components which may be essential in preventing the irreversible consequences of continuous excessive p53 activation^33^.

Our model describes several crosstalk pathways between the DNA damage checkpoint mechanism and cell cycle regulators. The activation of the p53 transcription factor directly induces the expression of p21 protein known as the cyclin-dependent protein kinase (CDK) inhibitor whose activity can induce a cell cycle arrest^36–38^. The induction of p21 by p53 indirectly represses Mad2 expression thus affecting the mitotic checkpoint complex (MCC) Mad2:Cdc20P^39^. However, the activation of p53 in response to a DNA damage signal is countered by active Plk1 that can physically bind and inhibit the p53 activity^40^. The phosphatase, Cdc25 plays a critical role in the G2/M DNA damage checkpoint with the damage signal (S3) disrupting Cdc25-mediated dephosphorylation CDK1 subunit of CyclinB:CDK1 complex (maturation-promoting factor MPF)^41,42^. MPF activates Plk1 as well as promotes the binding of Cdc20 to the anaphase-promoting complex/cyclosome APC/C (i.e., APC/CP). The resulting complex then triggers a final sequence of cell cycle events that eventually leads to the cell division^43,44^. In the same direction, Plk1 assists the formation of the APC/CP:Cdc20 complex (see Fig. 1) by both the induction of Mad2:Cdc20P complex disassembly via p31 comet^44–46^ and the activation of APC/C through downregulation of the APC/C inhibitor, Emi1^8,47^. In our model, Mad2:Cdc20P complex represents the MCC which is an important part of the spindle assembly checkpoint regulation^48^. Because Mad2:Cdc20P complex inhibits the cell cycle progression, we used it to detect the cell cycle arrest. Importantly, the role of Plk1 is not limited only to the Mad2:Cdc20P complex disassembly and the APC/C activation. It has also been shown that Plk1 depletion induces DNA damage^8^, and hence, there is a feedback loop involving Plk1 and the DNA damage checkpoint response mechanism.

**Fig. 2:**
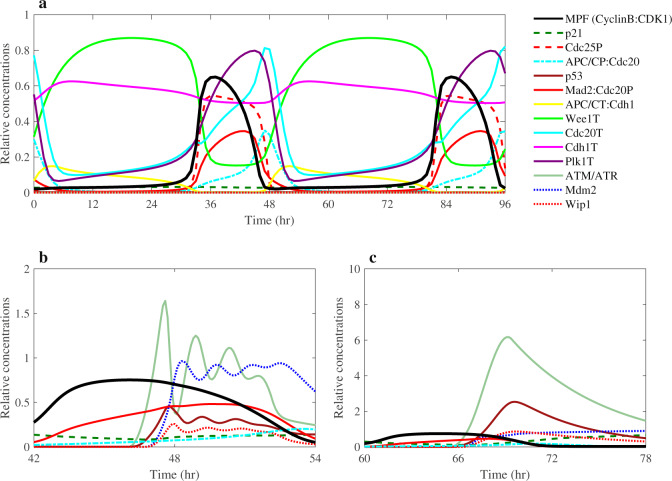
Numerical simulations of the mitotic cell cycle and G2/M DNA damage checkpoint regulation models. **a** Dynamic behavior of the mitotic cell cycle and DNA damage checkpoint components. **b**, **c** Numerical simulations of protein concentrations in Plk1-depleted cells. Plk1 concentration was reduced by 70% relative to its level in p53-wild-type cancer cells. The depletion of Plk1 induces DNA damage response and prompts the increase in the concentration of related regulators. **b** ATM, p53, Mdm2, and Wip1 exhibit oscillatory dynamics. **c** The oscillatory dynamics disappeared when the negative regulation of Wip1 on ATM was disrupted. These simulation results agree with similar observations reported in refs. ^32,35^. All concentrations in these plots are expressed relative to CDK1 concentration.

We assembled together all these individual molecular interactions to develop the G2/M DNA damage checkpoint regulation molecular network shown in Fig. 1. We then integrated this network with our recently developed molecular network of the mitotic cell cycle regulation^9^. All the molecular interactions are listed in Supplementary Table 1. Our integrated computational model of the complete regulatory molecular network that includes crosstalk between Plk1, p53, DNA damage checkpoint, and other key mitotic cell cycle regulators is based on ODEs as described in the “Methods” section. Our model of the G2/M DNA damage checkpoint regulation and mitotic cell cycle in cancer cells includes 34 ODEs and 137 kinetic parameters (see Supplementary Tables 2 and 3). We determined the values of the adjustable kinetic parameters of the model under the constraint that the cell cycle regulatory components exhibit limit-cycle dynamics (see Supplementary Fig. 1 and Supplementary Table 4), with correct timing and relative levels of major cell cycle regulators. For example, as shown in Fig. 2a, our model is in agreement qualitatively with experimentally observed relative concentration ranges and timing for Cdc25 phosphatase, Wee1, APC/CP:Cdc20 complex, and CyclinB:CDK1 that are major elements determining the mitotic cell cycle oscillatory behavior^49^. We required that our model correctly explains both the dynamics of the mitotic cell cycle components and oscillatory behavior of p53, Mdm2, and Wip1 components of the DNA damage checkpoint regulation module under different conditions (Fig. 2). The oscillatory behavior of p53 is well known and has been extensively studied^32,33^. Such oscillations have been observed when the DNA damage is induced by UV or gamma radiations^32,33^. The feedback loops between p53 and its transcriptional targets Mdm2 and Wip1 have been proposed to explain the p53 oscillatory dynamics^32,33^. Without the other cell cycle regulatory components, our module containing only the p53, Mdm2 and Wip1 components was able to produce an oscillation with a period of ~5 h (Supplementary Fig. 2a). We also observed p53 oscillations that are induced in the mitotic cell cycle phase due to the DNA-damages induced by Plk1 depletion (Fig. 2b). These oscillations have varying periods that decrease as Plk1 depletion increases (Supplementary Fig. 2b–f). Also, consistent with published observations^32,35^, if the negative regulation of Wip1 on ATM/ATR was disrupted, then p53 oscillations disappeared (Fig. 2c). The numerical simulations in Fig. 2b, c were obtained under the condition of Plk1 depletion which corresponded to 30% of Plk1 concentration in unperturbed cancer cells. The Plk1 depletion was used to activate the DDR.

### Effect of Plk1 depletion on different cancer cell lines

**Fig. 3:**
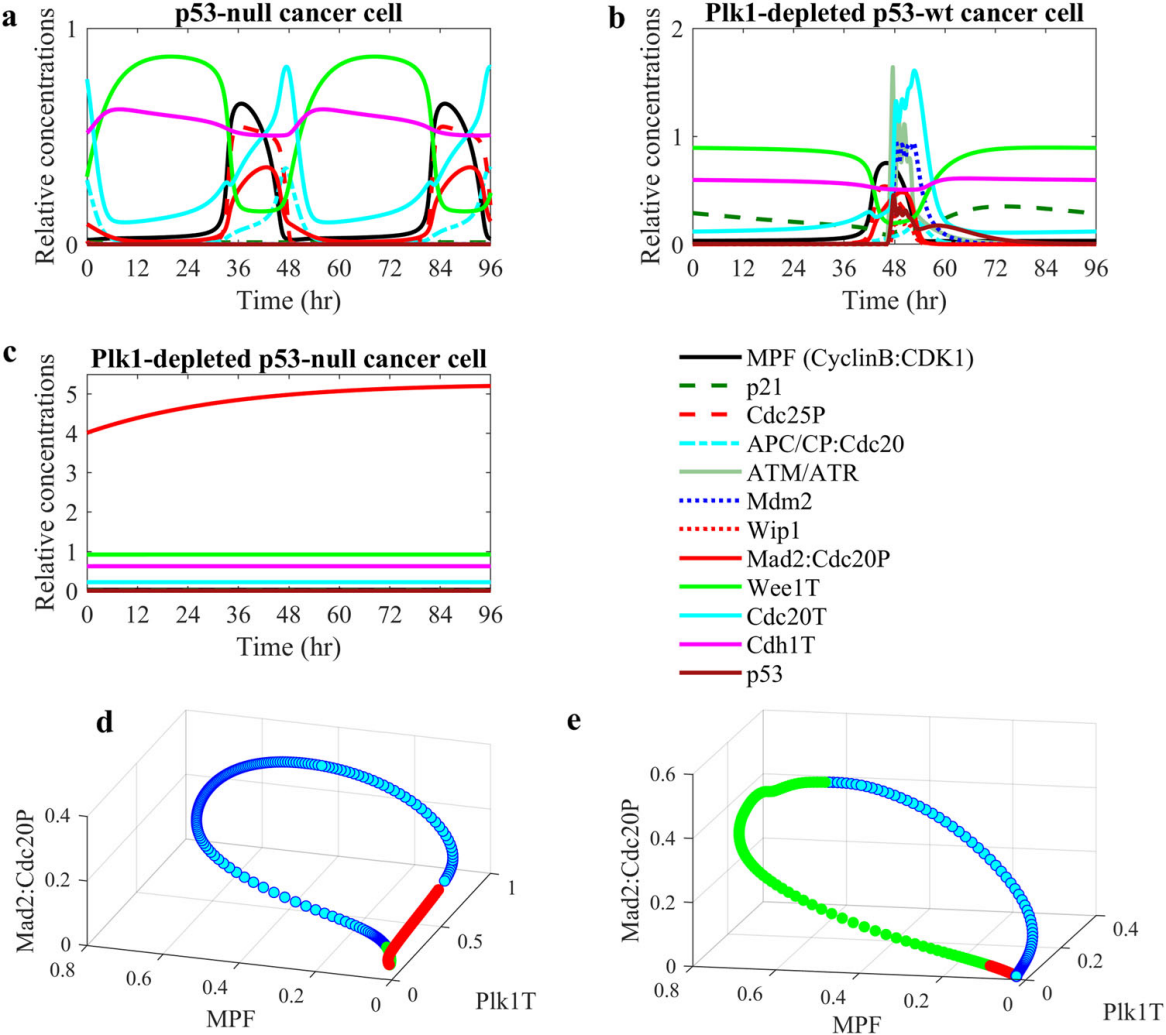
Numerical simulations of Plk1 depletion in p53-wild-type and p53-null cancer cells. **a** Dynamic behavior of model components in p53-null cancer cells. **b** Plk1-depleted p53-wt cancer cells. **c** Plk1-depleted p53-null cancer cells. The phase plots (**d**) and (**e**) show the limit cycles that correspond to simulations of p53-null and Plk1-depleted cells in (**a**) and (**b**). The limit cycle oscillations are directed counterclockwise from red to green. In the Plk1-depletion condition, Plk1 level was reduced by 70% relative to Plk1 level in cancer cells under normal conditions.

Next, we used the model to study the effect of Plk1 depletion on different cancer cell lines. Our analysis of mutations in approximately 1800 different cell lines available in the Cancer Dependency Map database revealed that 1091 cell lines have p53 mutation. Figure 3 shows the effect of Plk1 depletion in p53-null and p53-wild-type (p53-wt) cancer cell lines. The results from the model agreed with the experimental data showing that Plk1 depletion delays entry into mitosis in cells with p53-wt (Fig. 3b), whereas it induces the cell cycle arrest in p53-null cancer cells^5^ (Fig. 3c). These results can be compared with the numerical simulations of p53-wt cells (Fig. 2a) and p53-null cancer cells with normal Plk1 levels (Fig. 3a). The exit from mitosis requires inactivation of MPF which depends on the level of active APC/C:Cdc20 complex. The mitotic arrest-deficient protein Mad2 inhibits Cdc20 through the Mad2:Cdc20P complex formation. Therefore, we can detect the cell cycle arrest by monitoring the level of Mad2:Cdc20P complex. For example, the Mad2:Cdc20P complex level is constantly high in the arrested Plk1-depleted p53-null cancer cells (Fig. 3c) relative to the Mad2:Cdc20P complex level in dividing cells (Fig. 3a, b). Figure 3d, e shows limit cycle dynamic behaviors of Mad2:Cdc20P in p53-null and Plk1-depleted p53-wt cancer cells. In these phase plots, the vertical axis (*z*-axis) represents the level of Mad2:Cdc20P complex while Plk1 and p53 are plotted along *x* and *y* axes. Because Mad2:Cdc20P complex level increases as Plk1 is depleted and is constantly high in arrested cells (see Fig. 3c), we can use the Mad2:Cdc20P complex level as an indicator of the cell cycle arrest.

**Fig. 4:**
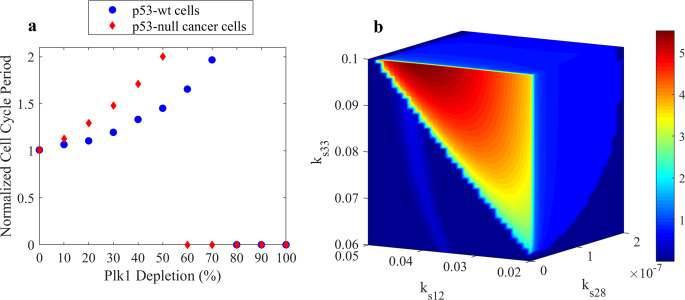
The effect of Plk1 depletion on the cell cycle progression in p53-wild-type and p53-null cancer cells. **a** The change of the cell cycle period in Plk1-depleted p53-wt (blue circles) and p53-null cancer (red diamonds) cells. The cell cycle periods of Plk1-depleted cells were computed for 10–100% Plk1-depletion levels and normalized by the cell cycle periods of corresponding unperturbed cells. Plk1 depletion above 50% causes a cell cycle arrest in p53-null cancer and only a cell cycle progression delay in p53-wt cancer cells. **b** The dependence of Mad2:Cdc20P complex concentration (relative to CDK1 concentration) on p53 synthesis rate (*k*_s28_), Plk1 synthesis rate (*k*_s12_), and Mad2 synthesis rate (*k*_s33_) parameters controlling levels of p53, Plk1, and Mad2 in cells. Mad2:Cdc20P complex concentration values are shown in the color bar and are highest (red) for *k*_s28_ = 0, *k*_s12_ = 0, and *k*_s33_ = 0.1 when p53 and Plk1 concentrations are low and Mad2 concentration is high. The color code bar shows the level of Mad2:Cdc20P complex concentration.

To quantify the effects of Plk1 depletion on the cell cycle progression in cancer cells, we simulated the cell cycle period as a function of Plk1-depletion level in the p53-wt and p53-null cancer cells (Fig. 4a). We found that the cell cycle period in p53-null cancer cells is more sensitive to Plk1 depletion than in p53-wt cells. Figure 4a shows the normalized cell cycle period (relative to the cell cycle period of unperturbed cells) as a function of the Plk1 synthesis rate which is reduced by 10–100% relative to the original rate constant value. Both p53-wt and p53-null cells show that the cell cycle period increases as the Plk1 synthesis rate decreases. However, the cell cycle period increases faster in p53-null cells than in p53-wt cells. Also, the cell cycle arrest of p53-null cells occurs when Plk1 synthesis rate parameter ${{{{{\mathrm{k}}}}}}_{{{{{{\mathrm{s}}}}}}12}$ is reduced by 60%, while p53-wt cells exhibit cell cycle arrest when Plk1 depletion reaches 80% (Fig. 4a).

Considering that we do not know the actual p53 activity in any specific cancer cells, we simulated the model results for different levels of p53 and found parameter ranges for which our model can produce the cell cycle arrest (Fig. 4b). The simulation results shown in Fig. 4b reveal that Mad2:Cdc20P complex concentration is highest when both p53 and Plk1 synthesis rate constants are close to zero. This result agrees with the experimental observation that p53-wt cancer cells are less sensitive to Plk1 depletion than p53-null cancer cells.

### CRISPR perturbations in different cell lines

**Fig. 5:**
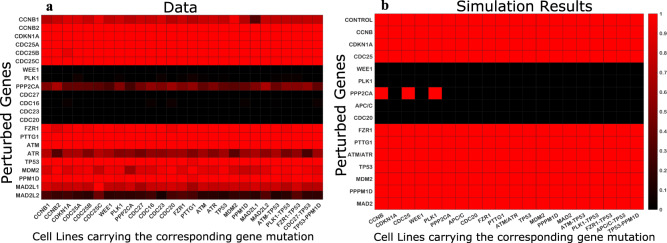
Heatmaps showing the gene perturbation results in different cancer cell lines. **a** The results derived from CRISPR (Avana) Public 20Q4 perturbation data analysis. The data were downloaded from Cancer Dependency Map database (depmap.org). **b** The model simulation results. The perturbed genes are listed along the *y*-axis, the cell lines carrying mutations in specific genes listed along the *x*-axis. The color scale shows whether the gene is reported essential or not. The black color shows that a gene is essential and the red color shows that a gene is not essential. For the heatmap representing the experimental data (**a**), we counted how many times a gene is reported as essential in each cell line carrying a specific gene mutation. For example, PLK1 gene is reported to be essential in all cell lines (black line), while ATR gene is essential in most cell lines carrying CCNB1 gene mutation (the heatmap rectangular cell is colored closer to black than red) but not in cell lines carrying CCNB2 mutation (the heatmap rectangular cell is colored closer to red than black). For the model results (**b**), the rectangular cell of the heatmap is colored black if a cell cycle arrest was observed and is colored red if the normal cell cycle progression was observed when genes were perturbed. The control represents unperturbed cancer cells. All unperturbed cancer cell lines were observed to be progressing through the cell cycle, thus are shown by red colored rectangular cells of the heatmap on the right.

Next, we analyzed mutation data in 1749 cancer cell lines and CRISPR data in 808 cancer cell lines from Cancer Dependency Map database (depmap.org). We observed that the TP53 gene is most frequently mutated in different cancer cell lines (1091 out of 1749 total characterized cell lines). CRISPR datasets are available for 549 cell lines carrying TP53 mutation. The second and third most frequent mutations found in different cancer cell lines are ATM and ATR. There are 267 and 184 cell lines carrying ATM and ATR mutations, respectively; among which 136 ATM and 92 ATR mutated cell lines have CRISPR data. Supplementary Table 5 summarizes the number of cell lines carrying specific mutations for which CRISPR datasets are available. Also, cancer cell lines carry mutations in many other genes. For example, we found 106 cell lines that carry both ATM and TP53 mutations and 24 cell lines carry PLK1 and TP53 mutations. We organized different cell lines into groups using mutation data for genes that are included in our model. Because our mathematical model includes 15 different genes, we can represent different cell lines by mutations in these genes. We have 15 distinct groups of cell lines that carry the corresponding 15 single-gene mutations. To demonstrate that our model can be used to simulate cell lines carrying several gene mutations, we simulated a few cell lines that carry two mutated genes. We also simulated the effect of gene deletions in different cell lines and compared the simulation results with CRISPR (Avana) Public 20Q4 perturbation data from Cancer Dependency Map database (depmap.org). The CRISPR perturbation of a gene was modeled by setting the synthesis rate parameter to zero for the corresponding gene in the model. A reduced value for the synthesis rate parameter was used if the model component can be represented by several genes (e.g., Cyclin B model component represents the products of CCNB1 and CCNB2 genes, Cdc25 represents the products of CDC25A and CDC25B genes). Also, APC/C complex has several different subunits produced by several genes (e.g., CDC27, CDC23, and CDC16). We assumed that the CRISPR perturbation of any gene that regulates the production of a complex subunit can disrupt the function of the complex. The data analysis and the simulation results are represented using the heatmaps shown in Fig. 5.

Our simulation results largely agreed with CRISPR perturbation data (see Fig. 5). The genes that are essential (with CERES dependency score >−1, see depmap.org for CERES definition) in different cancer cell lines are also essential for the cell cycle progression in our model.

### Sensitivity analysis of G2/M DNA damage checkpoint regulation

We used logarithmic sensitivity, partial rank correlation coefficients (PRCC), and fuzzy logic analysis methods to identify molecular components that strongly affect the cell cycle progression and whose perturbations can cause cell cycle arrest in cancer cells. The sensitivity analysis methods are described in the “Methods” section. First, we computed the average logarithmic sensitivity intensities (defined in “Methods”) for all model components by varying 137 model adjustable parameters in p53-wt, p53-null, and Plk1-depleted cells. The results are provided in Supplementary Tables 6 and 7, and Supplementary Fig. 3.

**Fig. 6:**
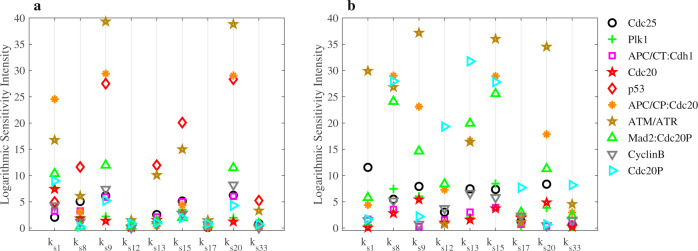
Logarithmic intensities for Cdc25, Plk1, APC/CT:Cdh1, Cdc20, ATM/ATR, Mad2:Cdc20P, p53, APC/CP:Cdc20, Cyclin B, and Cdc20P regulators. **a** p53-wild-type cancer cells. **b** p53-null cancer cells under the Plk1-depletion condition (Plk1 is depleted by 45% relative to Plk1 level in wild-type cells). The sensitivity intensities are obtained by varying *k*_s1_, *k*_s8_, *k*_s9_, *k*_s12_, *k*_s13_, *k*_s15_, *k*_s17_, *k*_s20_, and *k*_s33_ parameters that control the synthesis rates of Cyclin B, Cdc25, Wee1, Plk1, PP2A, APC/C, Cdc20, Cdh1, and Mad2, correspondingly.

We selected ten G2/M DNA damage checkpoint- and cell cycle-related regulators: Cdc25, Plk1, Cdc20, Cdc20P, APC/CT:Cdh1, ATM/ATR, Mad2:Cdc20P, p53, Cyclin B, and APC/CP:Cdc20 for which parameter variations produce different values of logarithmic intensities in p53-wt and p53-null cancer cells (Fig. 6) under condition of Plk1 depletion that activates DNA damage checkpoint. Perturbations of the following parameters: *k*_s1_, *k*_s8_, *k*_s9_, *k*_s12_, *k*_s13_, *k*_s15_, *k*_s17_, *k*_s20_, and *k*_s33_ induce significant changes in the G2/M DNA checkpoint-related regulators. These parameters control synthesis rates of Cyclin B, Cdc25, Wee1, Plk1, PP2A, APC/C, Cdc20, Cdh1, and Mad2, correspondingly. Other regulators are either not very sensitive to parameter variations or their changes are similar in both p53-wt and p53-null cancer cells (see Supplementary Fig. 4). Our results in Fig. 6 reveal that Mad2:Cdc20P, APC/CP:Cdc20, Cdc20, Cdc25, and ATM/ATR are more sensitive to parameter perturbations in p53-null cancer cell lines than in p53-wt cells. The concentration level of these proteins changes significantly in p53-null cancer cell lines when parameters are perturbed just within 1% of their corresponding values. Our sensitivity analysis results for Cdc20 are also in line with experimental observation^50^ showing that Cdc20 is upregulated in many types of p53-deficient cancer cells. We can also conclude that the higher sensitivity of Mad2:Cdc20 complex in p53-null cancer cells indicates that p53-null cancer cells are more susceptible to perturbations than p53-wt cancer cells. As suggested above, the high level of Mad2:Cdc20P complex can be used as an indicator of cell cycle arrest (see Figs. 3c and 4b). Both Plk1 and p21 inhibit the formation of Mad2:Cdc20P (see Fig. 1). Because p21 production is activated by p53 protein, p53-null cancer cells rely merely on Plk1 in regulating the level of Mad2:Cdc20P complex. Therefore, p53-null cancer cells are more susceptible to perturbations under the Plk1-depleted conditions.

We confirmed the main conclusions derived from the logarithmic sensitivity analysis by performing PRCC and fuzzy logic sensitivity analyses. PRCC is a global sensitivity analysis that reveals correlation between proteins and varied parameters^51,52^. The results of PRCC analysis are shown in Supplementary Fig. 5. We observed that the number of parameters that influence proteins (globally) were reduced in p53-null cells. The additional reduction in global correlations occurred when Plk1 was depleted. This indicates that the “active” wiring between nodes in the network describing cell cycle and G2/M DNA damage mechanisms is reduced when p53 is deleted and/or Plk1 is depleted.

In the Fuzzy logic analysis, the model parameters are perturbed by assigning a fuzzy uncertain number (with triangular membership function) instead of a crisp value for the model parameters (see “Methods” section). The analysis allowed us to determine the maximum uncertainty band of protein concentrations when specific parameters were perturbed. We performed this analysis for p53-null and p53-wt cancer cells in Plk1-normal and Plk1-depleted conditions. In agreement with the logogriphic sensitivity results, we observed that the following key regulators: ATM/ATR, Mad2:Cdc20P, APC/CP:Cdc20, Cdc25, and Cdc20P are significantly more sensitive to parameter perturbations in p53-null cancer cells as compared to p53-wt cancer cells when both cell types are tested in the Plk1-depletion condition (see Supplementary Fig. 6). The higher sensitivity of p53-null cells occurs only in stressful (Plk1 depletion) conditions, while in the normal Plk1 level condition, the sensitivity profiles of p53-null and p53-wt are comparable (see Supplementary Fig. 7). Overall, the Fuzzy logic sensitivity analysis supports our results and conclusions derived from the logarithmic sensitivity analysis.

### Model predictions of mutant phenotypes

We used our model to predict phenotypes of cancer cells that carry different mutations including gene deletions, deletion or inhibition of phosphorylation cites, deletion of distraction boxes and induced perturbations of other interactions described in the model. All mutations and perturbations that are described by the 137 model parameters are listed in Supplementary Table 8.

**Fig. 7:**
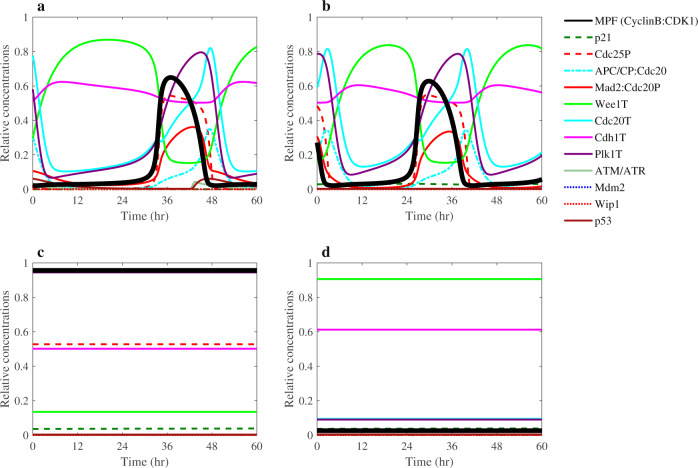
Dynamic behavior of cell cycle regulators in mutants. **a** CDKN1A gene deletion mutant is viable. **b** PTTG1 gene deletion mutant is viable with cell cycle comparable to that in wild-type cells (see Table 1). **c** CDC20 gene deletion mutant shows M-phase cell cycle arrest with high level of MPF. **d** CDC25 gene deletion induces early cell cycle arrest with low level of MPF.

The deletion of a gene that is nonessential for cell cycle progression is described as a viable phenotype, whereas the deletion of an essential gene leads to cell cycle arrest and an inviable phenotype. For example, Fig. 7 shows the dynamics of cell cycle components for CDKN1A, PTTG1, CDC20, and CDC25 gene deletion mutants. Loss of CDC20 gene or CDC25 gene results in cell cycle arrest, whereas loss of CDKN1A or PTTG1 gene does not significantly affect the cell cycle oscillations. Although, cells lacking CDKN1A exhibit cell cycle oscillations (see Fig. 7a), cells with elevated level of CDKN1A arrest (i.e., cell cycle oscillations disappear when p21 synthesis rate value *k*_s5_ is higher than 0.0073, see Supplementary Table 4). This result indicates that CDKN1A gene is an important cell cycle regulator that can be involved in the regulation of cell cycle progression. Cells lacking Cdc20 arrest in the M cell cycle phase with high level of MPF (see Fig. 7c). By contrast, cells lacking Cdc25 arrest in early in cell cycle phase with low levels of MPF (Fig. 7d). In our model, Cdc25 denotes all members of the CDC25 family.

We also found that p53-wt and p53-null cancer cells have the same set of essential genes for the cell cycle progression (see Table 1). This indicates that p53 deletion does not produce a stronger effect on the phenotype of gene deletion mutants. However, as we showed above, p53 is important for cell survivability under stressful conditions such as Plk1 depletion. Our model predicted that mutants with inviable phonotypes in the condition of Plk1 depletion (30% depletion is applied) remain inviable, however, the cell cycle period of mutants having viable phenotypes increases when Plk1 is depleted and this effect of the Plk1 depletion for some mutants is stronger in p53-null cells than in p53-wt cells (see Table 1). In addition, we gathered mutant data from the published literature and our model predictions agree with the phenotypic data on known gene deletion mutants (see Table 1). The model was also used to predict phenotypes of mutants that are not yet characterized in the published literature, for example the double gene deletion mutants (see Table 1) and mutants that carry other mutations in p53-null and p53-wt cancer cells (see Supplementary Table 8). Our analysis identified possible phenotypes of cancer cells perturbed by potential inhibitors or drugs targeting specific reactions in cell cycle and DNA damage checkpoint regulatory networks.

**Table 1:** Predicted phenotypes of some gene deletion mutants and comparison with data.

No	Genes or gene family	Proteins or complex	Mutant	p53-wt Cell phenotype^a^	p53-null Cell phenotype^a^	p53-wt Plk1-depleted cell phenotype^a^	p53-null Plk1-depleted cell phenotype^a^	Data and source
1	CCNB1 & CCNB2	Cyclin B	Cyclin B-null	Inviable	Inviable	Inviable	Inviable	Inviable^65,66^
2	CDKN1A	p21	p21-null	Viable *T*_m_/*T* = 1.0	Viable *T*_m_/*T* = 1.0	Viable *T*_m_/*T* = 1.3	Viable *T*_m_/*T* = 1.9	Viable^67^
3	CDC25	Cdc25	cdc25-null	Inviable	Inviable	Inviable	Inviable	Inviable^68^
4	WEE1	Wee1	wee1-null	Inviable	Inviable	Inviable	Inviable	Inviable^69^
5	PLK1	Plk1	plk1-null	Inviable	Inviable	Inviable	Inviable	Inviable^55^
6	PP2A	PP2A	PP2A-null	Inviable	Inviable	Inviable	Inviable	Inviable^70^
7	ANAPC1	APC/C	APC-null	Inviable	Inviable	Inviable	Inviable	Inviable^71^
8	CDC20	Cdc20	cdc20 -null	Inviable	Inviable	Inviable	Inviable	Inviable^72^
9	CDH1	Cdh1	cdh1-null	Viable *T*_m_/*T* = 0.3	Viable *T*_m_/*T* = 0.3	Viable *T*_m_/*T* = 0.3	Inviable	Suppression by RNAi is viable^73^
10	PTTG1	Pttg1	pttg1-null	Viable *T*_m_/*T* = 1.1	Viable *T*_m_/*T* = 1.1	Viable *T*_m_/*T* = 1.6	Viable *T*_m_/*T* = 2.1	Viable^74^
11	ATM	ATM	ATM-null	Viable *T*_m_/*T* = 1.0	Viable *T*_m_/*T* = 1.0	Viable *T*_m_/*T* = 1.4	Viable *T*_m_/*T* = 1.4	Viable^75^
12	TP53	p53	p53-null	Viable *T*_m_/*T* = 1.0	Viable *T*_m_/*T* = 1.0	Viable *T*_m_/*T* = 1.8	Viable *T*_m_/*T* = 1.8	Viable^55^
13	MAD2L1	Mad2	mad2-null	Viable *T*_m_/*T* = 1.0	Viable *T*_m_/*T* = 1.0	Viable, *T*_m_/*T* = 1.4	Viable *T*_m_/*T* = 1.8	Viable^76^
14	CDH1 & CDKN1A	Cdh1 & p21	cdh1-null p21-null	Inviable	Inviable	Inviable	Inviable	Prediction
15	CDKN1A PP2A	p21 & PP2A	p21-null PP2A-null	Inviable	Inviable	Inviable	Inviable	Prediction
16	CDKN1A WEE1	p21 & Wee1	p21-null wee1-null	Inviable	Inviable	Inviable	Inviable	Prediction
17	CDKN1A PTTG1	p21 & Pttg1	p21-null pttg1-null	Viable *T*_m_/*T* = 1.1	Viable *T*_m_/*T* = 1.1	Viable *T*_m_/*T* = 1.6	Viable *T*_m_/*T* = 2.2	Prediction
18	CDH1 MAD2	Cdh1 & Mad2	cdh1-null mad2-null	Inviable	Inviable	Viable *T*_m_/*T* = 0.3	Viable *T*_m_/*T* = 0.3	Prediction
19	CDC20 PTTG1	Cdc20 & Pttg1	cdc20-null pttg1-null	Inviable	Inviable	Inviable	Inviable	Prediction
20	CDC20 MAD2	Cdc20 & Mad2	cdc20-null mad2-null	Inviable	Inviable	Inviable	Inviable	Prediction
21	PP2A MAD2	PP2A & Mad2	PP2A-null mad2-null	Inviable	Inviable	Inviable	Inviable	Prediction
22	PP2A PTTG1	PP2A & Pttg1	PP2A-null pttg1-null	Inviable	Inviable	Inviable	Inviable	Prediction
23	CDC25 WEE1	Cdc25 & Wee1	cdc25-null wee1-null	Inviable	Inviable	Inviable	Inviable	Prediction

^a^*T*_m_/*T* is the ratio of cell cycle periods observed in the mutants *T*_m_ and in wild-type cancer cells *T*.

## Discussion

The major goals of this paper were: (i) to develop a computational model of the G2/M DNA damage checkpoint regulation; (ii) to uncover the effects of dynamic interactions between the cell cycle, Plk1, and p53 pathway regulators on the G2/M DNA damage checkpoint state; (iii) to explain Plk1-depletion-induced arrest and apoptosis in cancer cells; and (iv) to identify gene perturbations that induce a cell cycle arrest in different cancer cell lines. Understanding the regulation of the G2/M DNA damage checkpoint in cancer cells will help to predict novel therapeutic approaches against cancer.

We developed a computational model of the G2/M DNA damage checkpoint to study cell cycle progression in different cancer cell lines under various perturbations and conditions that include p53 and Plk1 depletions and CRISPR perturbations. Previous models of DNA damage checkpoint regulation explain oscillatory behavior of p53 transcription factor by giving special importance to regulatory feedback loops involving p53 targets^32,33,35,53^. These models describe a p53 regulatory mechanism with a few components responsible for oscillatory dynamics. By contrast, our DNA damage checkpoint regulation model is integrated with our recently developed model of the mitotic cell cycle regulation^9^, which allowed us not only to explain p53 oscillatory behavior but also to study crosstalk between DNA damage checkpoint and cell cycle regulators. The previous models have been aiming to explain p53 oscillations in response to DNA double- or single-strand breaks induced by UV or gamma radiations^32,33^. p53 oscillation is variable across different cell lines derived from different species (~3 h in mouse cells and ~5 h in human cells)^54^. Further, a Fourier analysis of p53 oscillations in human cells reveals three harmonics in Fourier spectrum of p53 dynamics with oscillation periods ~7, 3.5, and 2.4 h^53^. However, in our work we studied DNA damage checkpoint responses that are observed in conditions altering the cell cycle progression. We found that p53 oscillations can be induced by Plk1 depletion in M-phase of the cell cycle. We observed that the oscillation period decreases as the stress level caused by Plk1 depletion increases. The oscillation period varies and is ~1.1 h at high Plk1 depletion and increases to ~2 h as Plk1-depletion level decreases (Supplementary Fig. 2g, h). These p53 oscillations appear only in M-phase and their amplitude quickly decays as the cell cycle progresses through M-phase and the beginning of G1-phase.

In this paper, we extensively studied the role of p53 and Plk1 in the G2/M DNA damage checkpoint and cell cycle regulations. While the Plk1 and p53 pathways are often studied independently, our model allowed us to study the crosstalk between these pathways and their individual and cooperative regulatory functions. In our model, both p53 and Plk1 mediate Mad2 repression and thus allow cells to normally progress out of G2-phase to M-phase and then to anaphase. The correct functioning of p53 and Plk1 ensures that the Mad2 level is normal, which is important for the subsequent spindle assembly checkpoint regulation. However, the cell cycle progression in p53-deficient cancer cells relies merely on Plk1, wherefore Plk1 level in p53-deficient cancer cells is often higher than in normal or p53-wt cancer cells. Therefore, Plk1-depleted p53-deficient cancer cells arrest, while Plk1-depleted p53-wt cells still can progress out of G2- and M-phases. Our results are supported by the fact that the p53 pathway is known as a suppressor of chromosome instability. Chromosome instability can be caused by a high level of Mad2, and p53 prevents it by promoting Mad2 repression^39^. It has been also shown that Plk1 depletion induces DNA damage in both S and G2/M cell cycle phases^8^ and thus activates p53 pathway. Moreover, a normal level of Plk1 is also critical for maintaining chromosomal stability^55^. While the induction of DNA damage in Plk1-depleted p53-wt cancer cells can be countered with activation of the p53 pathway, Plk1 depletion can induce cell cycle arrest of p53-null cancer cells. Our simulation results agree with these experimental findings and also with the direct experimental observations^6,7^ showing that p53-deficient cancer cells are more sensitive to Plk1 depletion than normal cells and cancer cells with functional p53 protein.

Next, we performed three types of comprehensive sensitivity analyses for all regulators involved in the G2/M DNA damage checkpoint and cell cycle regulations. We identified regulators and parameters that strongly and distinctly affect the concentration of these regulators in p53-null and p53-wt cancer cells. The effect of parameter perturbations on proteins had been studied in p53-null and p53-wt cancer cells under the Plk1-depletion condition that induces the DNA damage checkpoint activation. We found that key proteins in p53-null cancer cells are more sensitive to parameter changes as compared to p53-wt cancer cells. The high sensitivity of ATM/ATR, Mad2:Cdc20P, and APC/CP:Cdc20 to parameter perturbations in p53-null cancer cells is due to the activation of DNA damage checkpoint. The level of ATM/ATR in p53-null cancer cell is much higher than that in p53-wt cells under the Plk1-depletion condition. The ATM/ATR dynamics in p53-null Plk1-depleted cells is similar to that shown in Fig. 2c, when the inhibition of ATM/ATR by Wip1 is reduced and DNA damage is activated, the level of ATM/ATR is several-fold higher than that in p53-wt cells shown in Fig. 2b. Because the synthesis of Wip1 is controlled by p53 transcription factor, p53-null cancer cells have low Wip1 levels, and the DNA damage-related proteins behave as in Fig. 2c. In addition, Mad2:Cdc20P is also an indicator of DNA damage checkpoint activation due to DNA replication stress in Plk1-depleted cells. And, because Mad2:Cdc20P has a direct influence on APC/CP:Cdc20 (Fig. 1), we observed that sensitivity intensities correlate for ATM/ATR, Mad2:Cdc20P, and APC/CP:Cdc20 regulators when DNA damage checkpoint is activated. Thus, we can conclude that the dynamic behavior of these regulators under perturbations is governed by DNA damage checkpoint activation.

Notably, we found that the cell-division cycle protein Cdc20P was also significantly more sensitive to some parameter perturbations in p53-null cancer cells. Specifically, Cdc20P in p53-null cancer cells is sensitive to parameters controlling production of Cdc25, PP2A, APC/C, and Plk1. It has been reported that Cdc20 is elevated in many p53-deficient cancer cells^50^. Kidokoro and co-workers suggested the negative regulation of Cdc20 by p53 to explain the elevation of Cdc20 level in p53-deficient cells and proposed Cdc20 protein as a potential cancer therapeutic target^50^. Our results support this observation, and the model also reveals the distinct sensitivity of Cdc20 protein in both p53-null and p53-wt cancer cells under the condition that induces DNA damage checkpoint activation. Our analysis suggests that the combination of Plk1 and one of the following proteins: Cdc25, PP2A, APC/C inhibitors, can be potentially used to induce the cell cycle arrest and apoptosis of p53-null cancer cell lines.

We also studied CRISPR perturbations and gene deletion mutants to identify gene perturbations that cause synthetic lethality. Our model is successful in explaining CRISPR perturbation data in different cancer cell lines. These results can be used to identify essential genes that can be considered as potential cancer therapeutic targets. The model can be used to test the effect of perturbations applied to multiple gene targets. We found that the deletion of CDC20, CDC25, or PLK1 genes is lethal for all cancer cell lines. Cdc25, Cdc20, and Plk1 are well-known direct targets for inhibitors that are used to induce a cell cycle arrest in cancer cells and tumor regression^56–59^. However, sensitivity analysis shows how to differentially affect these crucial regulators in different cancer cells, indirectly, by suppressing other cell cycle proteins.

Overall, our model and the results derived from sensitivity and mutation analyses can be used to predict effects of existing and potential treatments on the cell cycle progression in different cancer cell lines.

## Methods

### Model development

To build the G2/M DNA damage checkpoint regulatory molecular network, we summarized information from many publications on the individual interactions among relevant proteins and the associated gene regulatory mechanisms (Fig. 1). We then integrated this network with our recently developed molecular network for the mitotic cell cycle regulation^9^. The resulting reactions are described in Supplementary Table 1. We described the rates of individual reactions using the law of mass action or the Michaelis-Menten kinetics. The G2/M DNA damage checkpoint regulation mechanism in Fig. 1 along with the regulation mechanism of mitotic cell cycle are converted into a set of ODEs that determine how the state of the integrated control system evolves over time.

1 $$\frac{1}{\tau }\frac{{{{{\mathrm{d}}}}\left[ {{{{{{\mathrm{p21}}}}}}} \right]}}{{{{{\mathrm{d}}}}t}} = {{k}}_{{{{{{\mathrm{s}}}}}}5}\left( {1 + \frac{{\left[ {{{{{{\mathrm{p53}}}}}}} \right]}}{{K_{{{{{{\mathrm{p53}}}}}}}}}} \right) + 3\left( {{{k}}_{{{{{{\mathrm{f}}}}}}5}\left[ {{{{{{\mathrm{p21:MPF}}}}}}} \right] - {{k}}_{{{{{{\mathrm{r}}}}}}5}\left[ {{{{{{\mathrm{MPF}}}}}}} \right]\left[ {{{{{{\mathrm{p21}}}}}}} \right]^3} \right) - {{k}}_{{{{{{\mathrm{d}}}}}}5}\left[ {{{{{{\mathrm{p21}}}}}}} \right]$$

2 $$\begin{array}{l}\frac{1}{\tau }\frac{{{{{\mathrm{d}}}}\left[ {{{{{{\mathrm{Cdc25}}}}}}} \right]}}{{{{{\mathrm{d}}}}t}} = {{k}}_{{{{{{\mathrm{s}}}}}}8}\frac{{K_{{{{{{\mathrm{A2}}}}}}}}}{{K_{{{{{{\mathrm{A2}}}}}}} + \left[ {{{{{{\mathrm{ATM/ATR}}}}}}} \right]}} - {{k}}_{{{{{{\mathrm{f}}}}}}3}\left[ {{{{{{\mathrm{Cdc25}}}}}}} \right] + {{k}}_{{{{{{\mathrm{r}}}}}}3}\frac{{\left[ {{{{{{\mathrm{Cdc25P}}}}}}} \right]}}{{K_{{{{{{\mathrm{Cdc25P1}}}}}}} + \left[ {{{{{{\mathrm{Cdc25P}}}}}}} \right]}}\\ - \left( {{{k}}_{{{{{{\mathrm{d}}}}}}8.1} + {{k}}_{{{{{{\mathrm{d}}}}}}8.3}\frac{{\left[ {{{{{{\mathrm{APC/CT:Cdh1}}}}}}} \right]}}{{K_{{{{{{\mathrm{Cdc25}}}}}}} + \left[ {{{{{{\mathrm{Cdc25}}}}}}} \right]}}} \right)\left[ {{{{{{\mathrm{Cdc25}}}}}}} \right]\end{array}$$

3 $$\begin{array}{ll}\frac{1}{\tau }\frac{{{{{\mathrm{d}}}}\left[ {{{{{{\mathrm{Plk1P}}}}}}} \right]}}{{{{{\mathrm{d}}}}t}} = {{k}}^{\prime} _{{{{{{\mathrm{f6}}}}}}}\left[ {{{{{{\mathrm{Plk1}}}}}}} \right]\left[ {{{{{{\mathrm{MPF}}}}}}} \right]\frac{{K_{{{{{{\mathrm{A1}}}}}}}}}{{K_{{{{{{\mathrm{A1}}}}}}} + \left[ {{{{{{\mathrm{ATM/ATR}}}}}}} \right]}} - {{k}}_{{{{{{\mathrm{r6}}}}}}}\frac{{\left[ {{{{{{\mathrm{Plk1P}}}}}}} \right]}}{{K_{{{{{{\mathrm{Plk1P1}}}}}}} + \left[ {{{{{{\mathrm{Plk1P}}}}}}} \right]}}\\ \qquad\qquad\qquad- \,\left( {{{k}}_{{{{{{\mathrm{d11}}}}}}{{{{{\mathrm{.1}}}}}}} + {{k}}_{{{{{{\mathrm{d11}}}}}}{{{{{\mathrm{.3}}}}}}}\frac{{\left[ {{{{{{\mathrm{APC/CT:Cdh1}}}}}}} \right]}}{{K_{{{{{{\mathrm{Plk1P2}}}}}}} + \left[ {{{{{{\mathrm{Plk1P}}}}}}} \right]}}} \right)\left[ {{{{{{\mathrm{Plk1P}}}}}}} \right] - {{k}}_{{{{{{\mathrm{f17}}}}}}}\left[ {{{{{{\mathrm{p}}}}}}53{{{{{\mathrm{P}}}}}}} \right]\left[ {{{{{{\mathrm{Plk}}}}}}1{{{{{\mathrm{P}}}}}}} \right]\\ \qquad\qquad\qquad+\, {{k}}_{{{{{{\mathrm{r17}}}}}}}\left[ {{{{{{\mathrm{p}}}}}}53{{{{{\mathrm{P}}}}}}:{{{{{\mathrm{Plk}}}}}}1{{{{{\mathrm{P}}}}}}} \right]\end{array}$$

4 $$\begin{array}{ll}\frac{1}{\tau }\frac{{{{{\mathrm{d}}}}\left[ {{{{{{\mathrm{Cdc20P}}}}}}} \right]}}{{{{{\mathrm{d}}}}t}} = {{k}}_{{{{{{\mathrm{r9}}}}}}}\left[ {{{{{{\mathrm{Cdc20}}}}}}} \right] - {{k}}_{{{{{{\mathrm{f9}}}}}}}\frac{{\left[ {{{{{{\mathrm{Cdc20P}}}}}}} \right]}}{{{{{{{\mathrm{K}}}}}}_{{{{{{\mathrm{Cdc20P1}}}}}}} + \left[ {{{{{{\mathrm{Cdc20P}}}}}}} \right]}}\\ \qquad\qquad\qquad+\, {{k}}_{{{{{{\mathrm{r9}}}}}}}\left[ {{{{{{\mathrm{APC/CP:Cdc20}}}}}}} \right] - {{k}}_{{{{{{\mathrm{f18}}}}}}}\left[ {{{{{{\mathrm{Mad2}}}}}}} \right]\left[ {{{{{{\mathrm{Cdc20P}}}}}}} \right]\\ \qquad\qquad\qquad+\, {{k}}^{\prime} _{{{{{{\mathrm{r}}}}}}18}\left( {1 + \frac{{\left[ {{{{{{\mathrm{p21}}}}}}} \right]}}{{K_{{{{{{\mathrm{MCD}}}}}}}}} + \frac{{\varepsilon \left[ {{{{{{\mathrm{Plk1P}}}}}}} \right]}}{{K_{{{{{{\mathrm{MCD}}}}}}}}}} \right)\left[ {{{{{{\mathrm{Mad2:Cdc20P}}}}}}} \right]\\ \qquad\qquad\qquad- \,\left( {{{k}}_{{{{{{\mathrm{d18}}}}}}{{{{{\mathrm{.1}}}}}}} + {{k}}_{{{{{{\mathrm{d18}}}}}}{{{{{\mathrm{.3}}}}}}}\frac{{\left[ {{{{{{\mathrm{APC/CT:Cdh1}}}}}}} \right]}}{{{{{{{\mathrm{K}}}}}}_{{{{{{\mathrm{Cdc20P2}}}}}}} + \left[ {{{{{{\mathrm{Cdc20P}}}}}}} \right]}}} \right)\left[ {{{{{{\mathrm{Cdc20P}}}}}}} \right]\end{array}$$

5 $$\frac{1}{\tau }\frac{{{{{\mathrm{d}}}}\left[ {{{{{{\mathrm{ATM/ATR}}}}}}} \right]}}{{{{{\mathrm{d}}}}t}} = {{k}}_{{{{{{\mathrm{s}}}}}}27}\left( {1 + K_{{{{{{\mathrm{DDS}}}}}}}{{{{{\mathrm{Sig}}}}}}} \right) - \frac{{{{k}}_{{{{{{\mathrm{d}}}}}}27.1}\left[ {{{{{{\mathrm{ATM/ATR}}}}}}} \right]\left[ {{{{{{\mathrm{Wip1}}}}}}} \right]^4}}{{{{{{{\mathrm{K}}}}}}_{{{{{{\mathrm{Wip1}}}}}}}^4 + \left[ {{{{{{\mathrm{Wip1}}}}}}} \right]^4}} - {{k}}_{{{{{{\mathrm{d}}}}}}27}\left[ {{{{{{\mathrm{ATM/ATR}}}}}}} \right]$$

6 $$\begin{array}{l}\frac{1}{\tau }\frac{{{{{\mathrm{d}}}}\left[ {{{{{{\mathrm{p53}}}}}}} \right]}}{{{{{\mathrm{d}}}}t}} = {{k}}_{{{{{{\mathrm{s}}}}}}28}\left( {1 + \frac{{\left[ {{{{{{\mathrm{ATM/ATR}}}}}}} \right]}}{{K_{{{{{{\mathrm{A3}}}}}}}}}} \right) - {{k}}_{{{{{{\mathrm{f}}}}}}16}\left[ {{{{{{\mathrm{p53}}}}}}} \right] + {{k}}_{{{{{{\mathrm{r}}}}}}16}\left[ {{{{{{\mathrm{p53P}}}}}}} \right] - {{k}}_{{{{{{\mathrm{d}}}}}}28.1}\left[ {{{{{{\mathrm{p53}}}}}}} \right]\left[ {{{{{{\mathrm{Mdm2}}}}}}} \right]\\ - {{k}}_{{{{{{\mathrm{d}}}}}}28}\left[ {{{{{{\mathrm{p53}}}}}}} \right]\end{array}$$

7 $$\begin{array}{l}\frac{1}{\tau }\frac{{{{{\mathrm{d}}}}\left[ {{{{{{\mathrm{p53P}}}}}}} \right]}}{{{{{\mathrm{d}}}}t}} = {{k}}_{{{{{{\mathrm{f}}}}}}16}\left[ {{{{{{\mathrm{p53}}}}}}} \right] - {{k}}_{{{{{{\mathrm{r}}}}}}16}\left[ {{{{{{\mathrm{p53P}}}}}}} \right] + {{k}}_{{{{{{\mathrm{r}}}}}}17}\left[ {{{{{{\mathrm{p53P:Plk1P}}}}}}} \right] - {{k}}_{{{{{{\mathrm{f}}}}}}17}\left[ {{{{{{\mathrm{p53P}}}}}}} \right]\left[ {{{{{{\mathrm{Plk1P}}}}}}} \right]\\ - \,{{k}}_{{{{{{\mathrm{d}}}}}}29.1}\left[ {{{{{{\mathrm{p53P}}}}}}} \right]\left[ {{{{{{\mathrm{Mdm2}}}}}}} \right] - {{k}}_{{{{{{\mathrm{d}}}}}}29}\left[ {{{{{{\mathrm{p53P}}}}}}} \right]\end{array}$$

8 $$\frac{1}{\tau }\frac{{{{{\mathrm{d}}}}\left[ {{{{{{\mathrm{p53P:Plk1P}}}}}}} \right]}}{{{{{\mathrm{d}}}}t}} = {{k}}_{{{{{{\mathrm{f}}}}}}17}\left[ {{{{{{\mathrm{p53P}}}}}}} \right]\left[ {{{{{{\mathrm{Plk1P}}}}}}} \right] - {{k}}_{{{{{{\mathrm{r}}}}}}17}\left[ {{{{{{\mathrm{p53P:Plk1P}}}}}}} \right]$$

9 $$\frac{1}{\tau }\frac{{{{{\mathrm{d}}}}\left[ {{{{{{\mathrm{Mdm2}}}}}}} \right]}}{{{{{\mathrm{d}}}}t}} = {{k}}_{{{{{{\mathrm{s}}}}}}31} + {{k}}_{{{{{{\mathrm{s}}}}}}31.1}\left[ {{{{{{\mathrm{p53P}}}}}}} \right] - {{k}}_{{{{{{\mathrm{d}}}}}}31.1}\left[ {{{{{{\mathrm{ATM/ATR}}}}}}} \right]\left[ {{{{{{\mathrm{Mdm}}}}}}2} \right] - {{k}}_{{{{{{\mathrm{d}}}}}}31}\left[ {{{{{{\mathrm{Mdm2}}}}}}} \right]$$

10 $$\frac{1}{\tau }\frac{{{{{\mathrm{d}}}}\left[ {{{{{{\mathrm{Wip1}}}}}}} \right]}}{{{{{\mathrm{d}}}}t}} = {{k}}_{{{{{{\mathrm{s}}}}}}32} + {{k}}_{{{{{{\mathrm{s}}}}}}32.1}\left[ {{{{{{\mathrm{p53P}}}}}}} \right] - {{k}}_{{{{{{\mathrm{d}}}}}}32}\left[ {{{{{{\mathrm{Wip1}}}}}}} \right]$$

11 $$\begin{array}{l}\frac{1}{\tau }\frac{{{{{\mathrm{d}}}}\left[ {{{{{{\mathrm{Mad2}}}}}}} \right]}}{{{{{\mathrm{d}}}}t}} = {{k}}_{{{{{{\mathrm{s}}}}}}33} - {{k}}_{{{{{{\mathrm{f}}}}}}18}\left[ {{{{{{\mathrm{Mad2}}}}}}} \right]\left[ {{{{{{\mathrm{Cdc20P}}}}}}} \right] + {{k}}^{\prime} _{{{{{{\mathrm{r}}}}}}18}\left( {1 + \frac{{\left[ {{{{{{\mathrm{p21}}}}}}} \right]}}{{K_{{{{{{\mathrm{MCD}}}}}}}}} + \frac{{\varepsilon \left[ {{{{{{\mathrm{Plk1P}}}}}}} \right]}}{{K_{{{{{{\mathrm{MCD}}}}}}}}}} \right)\left[ {{{{{{\mathrm{Mad2:Cdc20P}}}}}}} \right]\\ - \,{{k}}_{{{{{{\mathrm{d}}}}}}33}\left[ {{{{{{\mathrm{Mad2}}}}}}} \right]\end{array}$$

12 $$\begin{array}{l}\frac{1}{\tau }\frac{{{{{\mathrm{d}}}}\left[ {{{{{{\mathrm{Mad2:Cdc20P}}}}}}} \right]}}{{{{{\mathrm{d}}}}t}} = {{k}}_{{{{{{\mathrm{f}}}}}}18}\left[ {{{{{{\mathrm{Mad2}}}}}}} \right]\left[ {{{{{{\mathrm{Cdc20P}}}}}}} \right]\\ \qquad\qquad\qquad\qquad- \,{{k}}^{\prime} _{{{{{{\mathrm{r}}}}}}18}\left( {1 + \frac{{\left[ {{{{{{\mathrm{p21}}}}}}} \right]}}{{K_{{{{{{\mathrm{MCD}}}}}}}}} + \frac{{\varepsilon \left[ {{{{{{\mathrm{Plk1P}}}}}}} \right]}}{{K_{{{{{{\mathrm{MCD}}}}}}}}}} \right)\left[ {{{{{{\mathrm{Mad2:Cdc20P}}}}}}} \right]\end{array}$$

where τ = 1.65 and other parameter values are provided in Supplementary Table 3. Here, we only show ODEs that are significantly modified (Eqs. (1)–(4)) in the mitotic cell cycle model^9^ and 8 new ODEs (Eqs. (5)–(12)) describing the G2/M DNA damage checkpoint regulation mechanism in Fig. 1. The complete description of our model is represented by 63 reactions, 34 ODEs, and 137 parameters provided in Supplementary Tables 1, 2, and 3, respectively. Because the concentration of total CDK1 in the cell is constant, concentrations of all proteins and protein complexes in the model are normalized with respect to the total CDK1 concentration which is set to 1.

### Model parameterization

For the mitotic cell cycle part, we started with 104 parameters which were determined in our recent study^9^. We changed the values of some parameters (specifically, 25 parameters were modified) in this basal parameter set in order to achieve a better agreement with experimental observations described in this work. We introduced 33 additional parameters to model the G2/M DNA damage checkpoint regulation mechanism. The new parameters were fine-tuned to reproduce experimental observations for wild-type cancer cells, Plk1-depleted wild-type cancer cells, as well as phenotypic data for known single-gene deletion mutants. We also required that mitotic cell cycle components continue to exhibit limit cycle oscillations^10^. We performed a sensitivity analysis using a limit cycle as the model output criterion. The model parameters were also varied to identify the ranges for which the limit cycle oscillations exist. Supplementary Fig. 1 and Supplementary Table 4 show that the limit cycle oscillations exist only for specific ranges of parameter values. As demonstrated in Supplementary Fig. 1, the limit cycle exists when *k*_s8_ parameter values is between 0.08 and 0.1, but not for values of *k*_s8_ less than or equal to 0.07 and greater than or equal to 0.5 indicating that there are Hopf bifurcation points between *k*_s8_ = 0.07 and *k*_s8_ = 0.5. For all parameters, we determined left and right endpoints of the interval that covers corresponding parameter values for which limit cycle oscillations exist (see Supplementary Table 4).

### Model simulations

We used MATLAB’s variable-step, variable-order solver ode15s to solve the set of 34 ODEs listed in Supplementary Table 2 with the parameter values as defined in Supplementary Table 3, and to produce the simulation results shown in Figs. 2–7. The MATLAB code used to generate all the results in this work is posted in GitHub at https://github.com/Yongwoon-Jung/PLK1.

To simulate a p53-null cancer cell, we set the synthesis parameter for p53 to zero (*k*_s28_ = 0) and initial condition p53(*t* = 0) = 0.

To simulate each mutant, we used exactly the same equations (Supplementary Table 2) and parameter values (Supplementary Table 3) except for those parameter changes that describe the mutation. For example, to simulate CDC20 gene deletion, we only set the parameter that describes the synthesis of Cdc20 to zero (*k*_s17_ = 0). To simulate the mutation that effects phosphorylation of protein X, we set the parameter that describes the phosphorylation rate constant for this protein to zero. To simulate a degron deletion for a protein X, we set the degradation for this protein to zero. In Supplementary Table 8, we listed all mutations and marked mutations that produce cell cycle arrest.

DNA damage response (DDR) in our model can be induced by Plk1 depletion, as has been shown in ref. ^8^. In the model, DDR activation is set to be a function of Mad2:Cdc20P complex that is influenced by Plk1. If Mad2:Cdc20P is greater than 0.6 (this is the cell cycle arrest and apoptosis condition), we set the concentration of Cdc20 = 0 and the strength of the DNA damage signal variable (DDS) is defined as DDS = 200(Mad2:Cdc20P–0.6), whereas if Mad2:Cdc20P is greater than 0.36 (DNA damage signal activation condition), DDS = 200(Mad2:Cdc20P–0.36). Also, the DNA damage signal is not activated (DDS = 0) if Mad2:Cdc20P is less than 0.36. The variable Sig in Eq. (5) for ATM/ATR is defined as ${{{{{\mathrm{Sig}}}}}} = {{{{{\mathrm{DDS}}}}}}e^{ - 0.00000001t}$^18^.

The timescale and the cell cycle period in our model are controlled by parameter ${\uptau}$. As reported by Cooperman and co-workers^60^, the cultured leukemic B cells had a heterogeneous division rate that ranged between once every 26 to once every 240 h. Because we also use the cell cycle part of the model from ref. ^9^, we set the same cell cycle period *T* = 48 h by τ = 1.65.

### Parameter sensitivity analysis

A state of our modeled system was determined by a specification of the concentrations of all its components. To characterize the sensitivity of the concentration $X_{P_i}^k$ of *k*th protein (*k* = 1…50) when the value *P*_*i*_ of *i*th parameter (*i* = 1…137) is changed, we used the logarithmic sensitivity intensity^61^, which is defined as

13 $$S_{P_i}^k = \frac{{{{{\mathrm{d}}}}\ln f_k\left( {P_i} \right)}}{{{{{\mathrm{d}}}}\ln P_i}} = \frac{{P_i}}{{f_k\left( {P_i} \right)}}\frac{{{{{\mathrm{d}}}}f_k\left( {P_i} \right)}}{{{{{\mathrm{d}}}}P_i}} \approx \frac{{P_i}}{{f_k\left( {P_i} \right)}}\frac{{\left| {f_k\left( {P_i + h} \right) - f_k\left( {P_i - h} \right)} \right|}}{{2h}}$$

where the function $f_k\left( {P_i} \right)$ represents the average concentration defined as

14 $$f_k\left( {P_i} \right) = \overline {X_{P_i}^k} = \frac{1}{N}\mathop {\sum }\limits_{j = 1}^N X_{P_i}^k\left( {t_0 + j \cdot \Delta t} \right).$$

We set the initial time *t*_0_ = 0, Δ*t* = 1 h and $N = {{{{{\mathrm{floor}}}}}}\left( {T/\Delta t} \right)$ where *T* is the integration time that is bigger than the cell cycle period and floor(X) is the floor function that returns the greatest integer less than or equal to the argument value. The integration time *T* was set to 96 h to cover the cell cycle in Plk1-depletion condition for which the cell cycle period could increase twice (see Fig. 4a). We varied each parameter by 1% of its value, therefore *h* = 0.01*P*_*i*_ in our analysis. The logarithmic intensities $S_{P_i}^k$ were computed for all 50 proteins including the total protein concentrations and protein complexes, and also 137 parameters, to identify components that can significantly alter limit cycle oscillations in the cell cycle system. Then, the average logarithmic sensitivity intensity $< S^k >$ for *k*th protein $X_{P_i}^k$, was defined as

15 $$< S^k > = \frac{1}{{137}}\mathop {\sum }\limits_{i = 1}^{137} S_{P_i}^k$$

The results for average logarithmic sensitivity for all 50 model components are provided in Supplementary Table 6 and also plotted in Supplementary Fig. 3 for normal, Plk1-depleted normal, p53-null cancer, and Plk1-depleted p53-null cancer cells.

#### Modeling different cancer cell lines and CRISPR perturbations

Different cancer cell lines were represented by specific gene mutations which were gathered from the Cancer Dependency Map Database (depmap.org). Each gene mutation was modeled by altering a synthesis rate constant for the mutated gene. If a gene was not essential for the cell cycle progression then the corresponding synthesis rate constant was set to zero. For example, for p53-null cancer cell lines, ${{k}}_{{{{{{\mathrm{s}}}}}}28} = 0$, for Mdm2-null cancer cell lines ${{k}}_{{{{{{\mathrm{s}}}}}}31} = 0$. We assumed that all modeled cancer cell lines must divide despite mutations in their genes. Therefore, for genes that are necessary for the cell cycle progression (e.g., Plk1) the values of synthesis rate constants were only reduced, ensuring that all cancer cells continue their cell cycle progression. All parameter values for synthesis rate constants that were used to represent gene mutations in different cancer cell lines are provided in Supplementary Table 9.

CRISPR perturbations were also modeled by adjusting synthesis rate parameters for perturbed genes. If a perturbed gene in the model corresponds to a family of genes (e.g., Cyclin B in the model represents the product of both CCNB1 and CCNB2 genes), then the perturbation of the gene was modeled by reducing the value of the corresponding synthesis rate constant. For example, perturbation of either CCNB1 or CCNB2 gene was modeled by reducing the value of the synthesis rate constant for Cyclin B. If a perturbed gene was represented by a unique gene, then the gene perturbation was modeled by setting the corresponding synthesis rate constant to zero. Supplementary Table 9 provides the synthesis rate constants that were used to simulate CRISPR perturbations.

### PRCC analysis

We also assessed the dependence of model components on parameters by computing partial rank correlation coefficients (PRCC) between the model outcome measures (50 state variables defined by ODEs and conservation laws) and model kinetic parameters (all 137 parameters). We applied a global sensitivity analysis algorithm developed in ref. ^51^ by randomly perturbing all parameter values simultaneously (±1%) and generating 1000 samples from these normal distributions using Latin hypercube sampling (LHS)^62^. Then, samples were used to compute PRCC values and the corresponding *p*-values for all model components. The sensitivity analysis was performed for p53-wt and p53-null cancer cells under Plk1-normal and -depleted conditions. The results of our global sensitivity analysis were represented in heatmaps shown in Supplementary Fig. 5. The PRCC analysis was performed for the following four cases: (1) p53-wt Plk1-normal, (2) p53-wt Plk1-depleted by 45%, (3) p53-null Plk1-normal, and (4) p53-null Plk1-depleted by 45%.

### Fuzzy analysis

To further analyze the dependence of model variables on parameter variations, we investigated the effect of uncertainty in parameter values on the model components by using the methods based on the fuzzy theorem^63,64^ by assigning a fuzzy number (with triangular membership function) instead of a crisp value for the model parameters. The results of Fuzzy analysis are shown in Supplementary Figs. 6 and 7. The vertical axes show the different α-cut levels where zero α-cut level value corresponds to a maximum parameter perturbation range (1%) and one corresponds to the minimum parameter perturbation level (0%). The horizontal axes show the maximum uncertainty band of protein concentrations. The analysis was performed for all model components under perturbation of *k*_s1_, *k*_s5_, *k*_s8_, *k*_s9_, *k*_s12_, *k*_s13_, *k*_s15_, *k*_s17_, *k*_s20_, *k*_s24_, *k*_s27_, *k*_s28_, *k*_s31_, *k*_s32_, and *k*_s33_ parameters that control the synthesis of Cyclin B, p21, Cdc25, Wee1, Plk1, PP2A, APC/C, Cdc20, Cdh1, Pttg1, ATM/ATR, p53, Mdm2, Wip1, and Mad2 proteins. As for PRCC analysis, we investigated for cases: (1) p53-wt Plk1-normal, (2) p53-wt Plk1-depleted by 45%, (3) p53-null Plk1-normal, and (4) p53-null Plk1-depleted by 45%. To compare how Plk1 depletion affects p53-wt and p53-null cells, we jointly present case#2 with case#4 (Supplementary Fig. 6) and case#1 with case#4 (Supplementary Fig. 7).

## Supplementary information

Supplementary information.

## Data Availability

The datasets generated and analyzed during the current study are available from the corresponding authors on reasonable request.

## Code availability

The MATLAB code used to generate all the results in this manuscript is posted in GitHub at https://github.com/Yongwoon-Jung/PLK1.
