# Supplementary material for: Crosstalk between Plk1, p53, cell cycle, and G2/M DNA damage checkpoint regulation in cancer: computational modeling and analysis
Source: NPJ Syst Biol Appl. 2021 Dec 9;7:46. doi: 10.1038/s41540-021-00203-8 (PMC8660825; doi:10.1038/s41540-021-00203-8)
Supplement: Supplementary file 1 — Supplementary information. [file 41540_2021_203_MOESM1_ESM.pdf]

Supplementary Information for the Manuscript

Crosstalk between Plk1, p53, Cell Cycle, and DNA Damage Checkpoint Regulation in Cancer: Computational Modeling and Analysis

Yongwoon Jung[1,#], Pavel Kraikivski[2,#,*], Sajad Shafiekhani[3], Scott S. Terhune[4,6], and Ranjan K. Dash[1,5,6,*]

[1]Department of Biomedical Engineering, Medical College of Wisconsin, Milwaukee, WI 53226, USA

[2]Academy of Integrated Science, Division of Systems Biology, Virginia Tech, Blacksburg, VA 24061, USA

[3]Department of Biomedical Engineering, School of Medicine, Tehran University of Medical Sciences, Tehran, Iran

[4]Departments of Microbiology and Immunology, Medical College of Wisconsin, Milwaukee, WI 53226, USA

[5]Department of Physiology, Medical College of Wisconsin, Milwaukee, WI 53226, USA

[6]Center of Systems and Molecular Medicine, Medical College of Wisconsin, Milwaukee, WI 53226, USA

[#]These authors contributed equally to this work as first authors

[*]These authors contributed equally to this work as senior authors

Correspondence: pavelkr@vt.edu; rdash@mcw.edu

Overview of Supplementary Materials

This supplemental text includes 9 Supplementary Tables and 8 sets of Supplementary Figures. Supplementary Tables and Supplementary Figures are presented in the order they are cited in the main text.

Our complete model consists 15 synthesis, 26 reactions of multiple degradations and 22 transformation reactions (Supplementary Table 1), 34 ODEs (Supplementary Table 2), and 137 parameters (Supplementary Table 3). Initial conditions are also provided in Supplementary Table 2.

Supplementary Figure 1 demonstrates that the limit cycle exists only for a specific range of parameter values. For example, the limit cycle is observed when k_{s8} parameter values is between 0.08 and 0.1, but not for values of k_{s8} less than or equal to 0.07 and greater than or equal to 0.5. These results indicate that there are Hopf bifurcation points between k_{s8}=0.07 and k_{s8}=0.5. For all other parameter.

Supplementary Table 4 provides left and right endpoints of the interval that covers corresponding parameter values for which limit cycle oscillations exist.

Supplementary Figure 2 shows the period of oscillations of ATM/ATR, p53, Wip1, Mdm2 regulators depending on Plk1 depletion levels.

Supplementary Table 5 provides the number of different cancer cell lines that carry a specific gene mutation from Dependency Map database (depmap.org) and the number of cell lines for which CRISPR data are available.

To compute the average logarithmic sensitivity intensity for k^{th} protein, we compute the logarithmic sensitivity intensity $S^k_{\mathrm{P}_i}$ (see definition in the main text) of this protein for every parameter P_i (i=1…137) and then define the average as:

$$< S^k >= \frac{1}{137} \sum_{i=1}^{137} S^k_{\mathrm{P}_i}$$

Supplementary Table 6 provides the average logarithmic sensitivity intensities for WT, Plk1-depleted WT, p53-null cancer, and Plk1-depleted p53-null cancer cells.

Supplementary Table 7 provides the list of twenty proteins with largest average logarithmic sensitivity intensities.

The average logarithmic sensitivity intensities for 50 model components are also shown in Supplementary Figure 3.

Supplementary Figure 4 shows the logarithmic sensitivity intensities for some important regulators involved in G2/M DNA damage checkpoint and cell cycle regulations as function of varied parameters that control expression of 15 proteins.

Supplementary Figure 5 show results of Partial Rank Correlation Coefficients (PRCC) analysis for the following four cases: 1) p53-wt Plk1-normal, 2) p53-wt Plk1 depleted by 45%, 3) p53-null Plk1-normal, 4) p53-null Plk1 depleted by 45%.

Supplementary Figure 6 and Supplementary Figure 7 show results of Fuzzy analysis for the following four cases: 1) p53-wt Plk1-normal, 2) p53-wt Plk1 depleted by 45%, 3) p53-null Plk1-normal, 4) p53-null Plk1 depleted by 45%. Case #2 and Case #4 are combined in Supplementary Figure 5. Case #1 and Case #3 are combined in Supplementary Figure 6.

Supplementary Table 8 provides predicted phenotypes of p53-wt and p53-null cancer cells that carry a mutation in the interaction described by the corresponding parameter that is set to 0. The mutation can induce the cell cycle arrest or cause the change in cell cycle period which is represented by the ratio T_m/T, where T_m is the mutant cell cycle period and T is the cell cycle period in wild type.

Supplementary Figure 8 and Supplementary Figure 9 show the effect of gene deletions on the phenotypes of p53-wt and p53-null cancer cells.

Supplementary Table 9 provides parameter values used to model cell lines and CRISPR perturbations.

The MATLAB code is posted in GitHub at https://github.com/Yongwoon-Jung/PLK1.

Supplementary Table 1. Reactions

$\xrightarrow{k_{s1}} \text{CycB} \xrightarrow{k_{d1.1},\ k_{d1.2}S_6,\ k_{d1.3}S_7}$<br><br>$S_6 = \text{APC/CP:Cdc20};\ S_7 = \text{APC/CT:Cdh1}$	(1)
$\text{CycB} + \text{CDK1} \underset{k_{r1}}{\overset{k_{f1}}{\rightleftharpoons}} \text{MPF}$	(2)
$\text{preMPF} \underset{k_{r2}}{\overset{k_{f2}}{\rightleftharpoons}} \text{MPF}$<br><br>$\text{MPF} \xrightarrow{k_{d3.1},\ k_{d3.2}S_6,\ k_{d3.3}S_7}$<br><br>$\text{preMPF} \xrightarrow{k_{d4.1},\ k_{d4.2}S_6,\ k_{d4.3}S_7}$<br><br>$k_{f2} = k'_{f2}[\text{Cdc25P}] + k''_{f2}[\text{Cdc25}]$<br><br>$k_{r2} = k'_{r2}[\text{Wee1}] + k''_{r2}[\text{Wee1P}]$	(3)
$\text{Cdc25} \underset{k_{r3}}{\overset{k_{f3}}{\rightleftharpoons}} \text{Cdc25P}$<br><br>$\text{Cdc25P} \xrightarrow{k_{d7.1},\ k_{d7.3}S_7}$<br><br>$\xrightarrow{k_{s8}S_3} \text{Cdc25} \xrightarrow{k_{d8.1},\ k_{d8.3}S_7}$<br><br>$k_{s8}S_3 = k_{s8}\dfrac{K_{A2}}{K_{A2} + [\text{ATM/ATR}]};$<br><br>$k_{f3} = k'_{f3}[\text{MPF}] + k''_{f3}[\text{Plk1P}];\ \ k_{r3} = k'_{r3}[\text{PP2A}]$	(4)
$\text{Wee1} \underset{k_{r4}}{\overset{k_{f4}}{\rightleftharpoons}} \text{Wee1P}$<br><br>$\xrightarrow{k_{s9}} \text{Wee1} \xrightarrow{k_{d9}}$<br><br>$\text{Wee1P} \xrightarrow{k_{d10}}$<br><br>$k_{f4} = k'_{f4}[\text{MPF}] + k''_{f4}[\text{Plk1P}];\ \ k_{r4} = k'_{r4}[\text{PP2A}]$	(5)

$p21{:}MPF \underset{k_{r5}}{\overset{k_{f5}}{\rightleftarrows}} 3p21 + MPF$<br>$\xrightarrow{k_{s5}S_5} p21 \xrightarrow{k_{d5}}$<br>$k_{s5}S_5 = k_{s5}\left(1 + [p53]/K_{p53}\right)$	(6)
$Plk1 \underset{k_{r6}}{\overset{k_{f6}}{\rightleftarrows}} Plk1P$<br>$Plk1P \xrightarrow{k_{d11.1},\ k_{d11.3}S_7}$<br>$\xrightarrow{k_{s12}} Plk1 \xrightarrow{k_{d12.1},\ k_{d12.3}S_7}$<br>$k_{f6} = k'_{f6}[MPF]\dfrac{K_{A1}}{K_{A1} + [ATM/ATR]};\ \ k_{r6} = k'_{r6}[PP2A]$	(7)
$PP2AP \underset{k_{r7}}{\overset{k_{f7}}{\rightleftarrows}} PP2A$<br>$\xrightarrow{k_{s13}} PP2A \xrightarrow{k_{d13}}$<br>$PP2AP \xrightarrow{k_{d14}}$<br>$k_{r7} = k'_{r7}[MPF]$	(8)
(i) $APC/C \underset{k_{r8}}{\overset{k_{f8}}{\rightleftarrows}} APC/CP$<br>$\xrightarrow{k_{s15}} APC/C \xrightarrow{k_{d15}}$<br>$APC/CP \xrightarrow{k_{d16}}$<br>(ii) $APC/C{:}Cdh1 \underset{k_{r8}}{\overset{k_{f8}}{\rightleftarrows}} APC/CP{:}Cdh1$<br>(iii) $APC/CP{:}Cdc20 \xrightarrow{k_{r8}} APC/C + Cdc20$<br>$k_{f8} = k'_{f8}[MPF] + k''_{f8}[Plk1P];\ \ k_{r8} = k'_{r8}[PP2A]$	(9)

(i) $\text{Cdc20P} \underset{k_{r9}}{\overset{k_{f9}}{\rightleftarrows}} \text{Cdc20}$<br><br>$\xrightarrow{k_{s17}} \text{Cdc20} \xrightarrow{k_{d17.1},\ k_{d17.3}S_7}$<br><br>$\text{Cdc20P} \xrightarrow{k_{d18.1},\ k_{d18.3}S_7}$<br><br>(ii) $\text{APC/CP:Cdc20} \xrightarrow{k_{r9}} \text{APC/CP} + \text{Cdc20P}$<br><br>$k_{r9} = k'_{r9}[\text{MPF}] + k''_{r9}[\text{Plk1P}];\ \ k_{f9} = k'_{f9}[\text{PP2A}]$	(10)
$\text{APC/CP} + \text{Cdc20} \underset{k_{r10}}{\overset{k_{f10}}{\rightleftarrows}} \text{APC/CP: Cdc20}$	(11)
(i) $\text{Cdh1P} \underset{k_{r11}}{\overset{k_{f11}}{\rightleftarrows}} \text{Cdh1}$<br><br>$\xrightarrow{k_{s20}} \text{Cdh1} \xrightarrow{k_{d20}}$<br><br>$\text{Cdh1P} \xrightarrow{k_{d21}}$<br><br>(ii) $\text{APC/C:Cdh1} \xrightarrow{k_{r11}} \text{APC/C} + \text{Cdh1P}$<br><br>(iii) $\text{APC/CP:Cdh1} \xrightarrow{k_{r11}} \text{APC/CP} + \text{Cdh1P}$<br><br>$k_{r11} = k'_{r11}[\text{MPF}] + k''_{r11}[\text{Plk1P}];\ \ k_{f11} = k'_{f11}[\text{PP2A}]\dfrac{K_{\text{Pttg1}}}{K_{\text{Pttg1}} + [\text{Pttg1}]}$	(12)
$\text{APC/C} + \text{Cdh1} \underset{k_{r12}}{\overset{k_{f12}}{\rightleftarrows}} \text{APC/C: Cdh1}$	(13)
$\text{APC/CP} + \text{Cdh1} \underset{k_{r13}}{\overset{k_{f13}}{\rightleftarrows}} \text{APC/CP: Cdh1}$	(14)
$\text{Pttg1P} \underset{k_{r14}}{\overset{k_{f14}}{\rightleftarrows}} \text{Pttg1}$<br><br>$\xrightarrow{k_{s24}} \text{Pttg1} \xrightarrow{k_{d24.1},\ k_{d24.2}S_6}$<br><br>$\text{Pttg1P} \xrightarrow{k_{d25.1},\ k_{d25.2}S_6}$	(15)

$k_{r14} = k'_{r14}[MPF]; \; k_{f14} = k'_{f14}[PP2A]$	
$LMNA \underset{k_{r15}}{\overset{k_{f15}}{\rightleftarrows}} LMNAP$<br><br>$k_{f15} = k'_{f15}[MPF]$	(16)
$\xrightarrow{k_{s27}DDS} ATM/ATR \xrightarrow{k_{d27},\; k_{d27.1}S_2}$<br><br>$k_{d27.1}S_2 = k_{d27.1}\frac{[Wip1]^4}{{K_{Wip1}}^4 + [Wip1]^4}$	(17)
$p53 \underset{k_{r16}}{\overset{k_{f16}}{\rightleftarrows}} p53P$<br>$\xrightarrow{k_{s28}S_3} p53 \xrightarrow{k_{d28},\; k_{d28.1}S_4}$<br><br>$p53P \xrightarrow{k_{d28},\; k_{d28.1}S_4}$<br><br>$S_4 = Mdm2$<br><br>$k_{s28}S_3 = k_{s28}(1 + [ATM/ATR]/K_{A3})$<br><br>$k_{f16} = k'_{f16}\frac{[ATM/ATR]^4}{{K_{A4}}^4 + [ATM/ATR]^4}; \; k_{r16} = k'_{r16}[Wip1]$	(18)
$\xrightarrow{k_{s31},\; k_{s31.1}S_8} Mdm2 \xrightarrow{k_{d31},\; k_{d31.1}S_3}$<br><br>$S_8 = p53P; S_3 = ATM/ATR$	(19)
$\xrightarrow{k_{s32},\; k_{s32.1}S_8} Wip1 \xrightarrow{k_{d32}}$	(20)
$p53P + Plk1P \underset{k_{r17}}{\overset{k_{f17}}{\rightleftarrows}} p53P{:}Plk1P$	(21)
$Mad2 + Cdc20P \underset{k_{r18}}{\overset{k_{f18}}{\rightleftarrows}} Mad2{:}Cdc20P$<br><br>$\xrightarrow{k_{s33}} Mad2 \xrightarrow{k_{d33}}$<br><br>$k_{r18} = k'_{r18}(1 + [p21]/K_{MCD} + \varepsilon[Plk1P]/K_{MCD})$	(22)

Supplementary Table 2. Model differential equations and initial conditions (τ=1.65: Time scaling factor)

$\frac{1}{\tau}\frac{d[\mathrm{CycB}]}{dt} = \mathrm{k_{s1}} + \mathrm{k_{r1}}[\mathrm{MPF}] - \mathrm{k_{f1}}[\mathrm{CycB}][\mathrm{CDK1}] - \left(\mathrm{k_{d1.1}} + \mathrm{k_{d1.2}}\frac{[\mathrm{APC/CP{:}Cdc20}]}{\mathrm{K_{CycB1}} + [\mathrm{CycB}]} + \mathrm{k_{d1.3}}\frac{[\mathrm{APC/CT{:}Cdh1}]}{\mathrm{K_{CycB2}} + [\mathrm{CycB}]}\right)[\mathrm{CycB}]$ [CycB](0)=0	(1)
$\frac{1}{\tau}\frac{d[\mathrm{CDK1}]}{dt} = -\mathrm{k_{f1}}[\mathrm{CycB}][\mathrm{CDK1}] + \mathrm{k_{r1}}[\mathrm{MPF}] + \left(\mathrm{k_{d3.1}} + \mathrm{k_{d3.2}}\frac{[\mathrm{APC/CP{:}Cdc20}]}{\mathrm{K_{MPF1}} + [\mathrm{MPF}]} + \mathrm{k_{d3.3}}\frac{[\mathrm{APC/CT{:}Cdh1}]}{\mathrm{K_{MPF2}} + [\mathrm{MPF}]}\right)[\mathrm{MPF}] + \left(\mathrm{k_{d4.1}} + \mathrm{k_{d4.2}}\frac{[\mathrm{APC/CP{:}Cdc20}]}{\mathrm{K_{preMPF1}} + [\mathrm{preMPF}]} + \mathrm{k_{d4.3}}\frac{[\mathrm{APC/CT{:}Cdh1}]}{\mathrm{K_{preMPF2}} + [\mathrm{preMPF}]}\right)[\mathrm{preMPF}]$ [CDK1T]=1 and [CDK1](0)=1	(2)
$\frac{1}{\tau}\frac{d[\mathrm{MPF}]}{dt} = \mathrm{k_{f1}}[\mathrm{CycB}][\mathrm{CDK1}] - \mathrm{k_{r1}}[\mathrm{MPF}] + \mathrm{k_{f2}}[\mathrm{preMPF}] - \mathrm{k_{r2}}[\mathrm{MPF}] + \mathrm{k_{f5}}[\mathrm{p21{:}MPF}] - \mathrm{k_{r5}}[\mathrm{p21}]^3[\mathrm{MPF}] + \mathrm{k_{d6.2}}\left(\frac{[\mathrm{APC/CP{:}Cdc20}]}{\mathrm{K_{p21MPF1}} + [\mathrm{p21{:}MPF}]}\right)[\mathrm{p21{:}MPF}] - \left(\mathrm{k_{d3.1}} + \mathrm{k_{d3.2}}\frac{[\mathrm{APC/CP{:}Cdc20}]}{\mathrm{K_{MPF1}} + [\mathrm{MPF}]} + \mathrm{k_{d3.3}}\frac{[\mathrm{APC/CT{:}Cdh1}]}{\mathrm{K_{MPF2}} + [\mathrm{MPF}]}\right)[\mathrm{MPF}]$ $\mathrm{k_{f2}} = \mathrm{k'_{f2}}[\mathrm{Cdc25P}] + \mathrm{k''_{f2}}[\mathrm{Cdc25}]$, $\mathrm{k_{r2}} = \mathrm{k'_{r2}}[\mathrm{Wee1}] + \mathrm{k''_{r2}}[\mathrm{Wee1P}]$, as in Supplementary Table 1. [MPF](0)=0	(3)

$\frac{1}{\tau}\frac{d[\mathrm{preMPF}]}{dt} = \mathrm{k_{r2}}[\mathrm{MPF}] - \mathrm{k_{f2}}[\mathrm{preMPF}] - \left(\mathrm{k_{d4.1}} + \mathrm{k_{d4.2}}\frac{[\mathrm{APC/CP{:}Cdc20}]}{\mathrm{K_{preMPF1}} + [\mathrm{preMPF}]} + \mathrm{k_{d4.3}}\frac{[\mathrm{APC/CT{:}Cdh1}]}{\mathrm{K_{preMPF2}} + [\mathrm{preMPF}]}\right)[\mathrm{preMPF}]$ [preMPF](0)=0	(4)
$\frac{1}{\tau}\frac{d[\mathrm{p21}]}{dt} = \mathrm{k_{s5}}\left(1 + [\mathrm{p53}]/\mathrm{K_{p53}}\right) + 3(\mathrm{k_{f5}}[\mathrm{p21{:}MPF}] - \mathrm{k_{r5}}[\mathrm{MPF}][\mathrm{p21}]^3) - \mathrm{k_{d5}}[\mathrm{p21}]$ [p21](0)=0	(5)
$\frac{1}{\tau}\frac{d[\mathrm{p21{:}MPF}]}{dt} = \mathrm{k_{r5}}[\mathrm{MPF}][\mathrm{p21}]^3 - \mathrm{k_{f5}}[\mathrm{p21{:}MPF}] - \mathrm{k_{d6}}\left(\frac{[\mathrm{APC/CP{:}Cdc20}]}{\mathrm{K_{p21MPF}} + [\mathrm{p21{:}MPF}]}\right)[\mathrm{p21{:}MPF}]$ [p21:MPF](0)=0	(6)
$\frac{1}{\tau}\frac{d[\mathrm{Cdc25P}]}{dt} = \mathrm{k_{f3}}[\mathrm{Cdc25}] - \mathrm{k_{r3}}\frac{[\mathrm{Cdc25P}]}{\mathrm{K_{Cdc25P1}} + [\mathrm{Cdc25P}]} - \left(\mathrm{k_{d7.1}} + \mathrm{k_{d7.3}}\frac{[\mathrm{APC/CT{:}Cdh1}]}{\mathrm{K_{Cdc25P2}} + [\mathrm{Cdc25P}]}\right)[\mathrm{Cdc25P}]$ $\mathrm{k_{f3}} = \mathrm{k'_{f3}}[\mathrm{MPF}] + \mathrm{k''_{f3}}[\mathrm{Plk1P}]$, $\mathrm{k_{r3}} = \mathrm{k'_{r3}}[\mathrm{PP2A}]$, as in Supplementary Table 1. [Cdc25P](0)=0	(7)
$\frac{1}{\tau}\frac{d[\mathrm{Cdc25}]}{dt} = \mathrm{k_{s8}}\frac{\mathrm{K_{A2}}}{\mathrm{K_{A2}} + [\mathrm{ATM/ATR}]} - \mathrm{k_{f3}}[\mathrm{Cdc25}] + \mathrm{k_{r3}}\frac{[\mathrm{Cdc25P}]}{\mathrm{K_{Cdc25P1}} + [\mathrm{Cdc25P}]} - \left(\mathrm{k_{d8.1}} + \mathrm{k_{d8.3}}\frac{[\mathrm{APC/CT{:}Cdh1}]}{\mathrm{K_{Cdc25}} + [\mathrm{Cdc25}]}\right)[\mathrm{Cdc25}]$ [Cdc25](0)=1	(8)

$\frac{1}{\tau}\frac{d[\mathrm{Wee1}]}{dt} = \mathrm{k_{s9}} + \mathrm{k_{r4}}\frac{[\mathrm{Wee1P}]}{\mathrm{K_{Wee1P}} + [\mathrm{Wee1P}]} - \mathrm{k_{f4}}[\mathrm{Wee1}] - \mathrm{k_{d9}}[\mathrm{Wee1}]$ <br>$\mathrm{k_{f4}} = \mathrm{k'_{f4}}[\mathrm{MPF}] + \mathrm{k''_{f4}}[\mathrm{Plk1P}],\ \mathrm{k_{r4}} = \mathrm{k'_{r4}}[\mathrm{PP2A}]$, as in Supplementary Table 1. <br>[Wee1](0)=1	(9)
$\frac{1}{\tau}\frac{d[\mathrm{Wee1P}]}{dt} = \mathrm{k_{f4}}[\mathrm{Wee1}] - \mathrm{k_{r4}}\frac{[\mathrm{Wee1P}]}{\mathrm{K_{Wee1P}} + [\mathrm{Wee1P}]} - \mathrm{k_{d10}}[\mathrm{Wee1P}]$ <br>[Wee1P](0)=0	(10)
$\frac{1}{\tau}\frac{d[\mathrm{Plk1P}]}{dt} = \mathrm{k_{f6}}[\mathrm{Plk1}] - \mathrm{k_{r6}}\frac{[\mathrm{Plk1P}]}{\mathrm{K_{Plk1P1}} + [\mathrm{Plk1P}]}$ <br>$- \left(\mathrm{k_{d11.1}} + \mathrm{k_{d11.3}}\frac{[\mathrm{APC/CT{:}Cdh1}]}{\mathrm{K_{Plk1P2}} + [\mathrm{Plk1P}]}\right)[\mathrm{Plk1P}] - \mathrm{k_{f17}}[\mathrm{p53P}][\mathrm{Plk1P}]$ <br>$+ \mathrm{k_{r17}}[\mathrm{p53P{:}\,Plk1P}]$ <br>$\mathrm{k_{f6}} = \mathrm{k'_{f6}}[\mathrm{MPF}]\frac{\mathrm{K_{A1}}}{\mathrm{K_{A1}}+[\mathrm{ATM/ATR}]},\ \mathrm{k_{r6}} = \mathrm{k'_{r6}}[\mathrm{PP2A}]$, as in Supplementary Table 1. <br>[Plk1P](0)=0	(11)
$\frac{1}{\tau}\frac{d[\mathrm{Plk1}]}{dt} = \mathrm{k_{s12}} + \mathrm{k_{r6}}\frac{[\mathrm{Plk1P}]}{\mathrm{K_{Plk1P1}} + [\mathrm{Plk1P}]} - \mathrm{k_{f6}}[\mathrm{Plk1}] - \mathrm{k_{d12.1}}[\mathrm{Plk1}]$ <br>$- \left(\mathrm{k_{d12.3}}\frac{[\mathrm{APC/CT{:}Cdh1}]}{\mathrm{K_{Plk1}}+[\mathrm{Plk1}]}\right)[\mathrm{Plk1}]$ <br>[Plk1](0)=1	(12)
$\frac{1}{\tau}\frac{d[\mathrm{PP2A}]}{dt} = \mathrm{k_{s13}} + \mathrm{k_{f7}}[\mathrm{PP2AP}] - \mathrm{k_{r7}}[\mathrm{PP2A}] - \mathrm{k_{d13}}[\mathrm{PP2A}]$ <br>$\mathrm{k_{r7}} = \mathrm{k'_{r7}}[\mathrm{MPF}]$, as in Supplementary Table 1. <br>[PP2A](0)=1	(13)
$\frac{1}{\tau}\frac{d[\mathrm{PP2AP}]}{dt} = \mathrm{k_{r7}}[\mathrm{PP2A}] - \mathrm{k_{f7}}[\mathrm{PP2AP}] - \mathrm{k_{d14}}[\mathrm{PP2AP}]$ <br>[PP2AP](0)=0	(14)

$$\frac{1}{\tau}\frac{d[\text{APC/C}]}{dt} = k_{s15} - k_{f8}[\text{APC/C}] + k_{r8}\frac{[\text{APC/CP}]}{K_{APC/CP} + [\text{APC/CP}]} + k_{r8}[\text{APC/CP:Cdc20}]$$ $$+ (k_{r11} + k_{r12})[\text{APC/C:Cdh1}] - k_{f12}[\text{APC/C}][\text{Cdh1}] - k_{d15}[\text{APC/C}]$$ $k_{f8} = k'_{f8}[\text{MPF}] + k''_{f8}[\text{Plk1P}]$, $k_{r8} = k'_{r8}[\text{PP2A}]$, as in Supplementary Table 1. [APC/C](0)=1	(15)
$$\frac{1}{\tau}\frac{d[\text{APC/CP}]}{dt} = k_{f8}[\text{APC/C}] - k_{r8}\frac{[\text{APC/CP}]}{K_{APC/CP} + [\text{APC/CP}]} - k_{f10}[\text{APC/CP}][\text{Cdc20}]$$ $$- k_{f13}[\text{APC/CP}][\text{Cdh1}] + (k_{r9} + k_{r10})[\text{APC/CP:Cdc20}]$$ $$+ (k_{r11} + k_{r13})[\text{APC/CP:Cdh1}] - k_{d16}[\text{APC/CP}]$$ [APC/CP](0)=0	(16)
$$\frac{1}{\tau}\frac{d[\text{Cdc20}]}{dt} = k_{s17} + k_{f9}\frac{[\text{Cdc20P}]}{K_{Cdc20P1} + [\text{Cdc20P}]} - k_{r9}[\text{Cdc20}]$$ $$+ (k_{r8} + k_{r10})[\text{APC/CP:Cdc20}] - k_{f10}[\text{Cdc20}][\text{APC/CP}]$$ $$- \left(k_{d17.1} + k_{d17.3}\frac{[\text{APC/CT:Cdh1}]}{K_{Cdc20} + [\text{Cdc20}]}\right)[\text{Cdc20}]$$ $k_{s17} = 0, \text{when Mad2: Cdc20P} \geq 0.6$ [Cdc20](0)=1	(17)
$$\frac{1}{\tau}\frac{d[\text{Cdc20P}]}{dt} = k_{r9}[\text{Cdc20}] - k_{f9}\frac{[\text{Cdc20P}]}{K_{Cdc20P1} + [\text{Cdc20P}]} + k_{r9}[\text{APC/CP:Cdc20}]$$ $$-k_{f18}[\text{Mad2}][\text{Cdc20P}] + k_{r18}[\text{Mad2:Cdc20P}]$$ $$- \left(k_{d18.1} + k_{d18.3}\frac{[\text{APC/CT:Cdh1}]}{K_{Cdc20P2} + [\text{Cdc20P}]}\right)[\text{Cdc20P}]$$ $k_{f9} = k'_{f9}[\text{PP2A}]$, $k_{r9} = k'_{r9}[\text{MPF}] + k''_{f9}[\text{Plk1P}]$ and $k_{r18} = k'_{r18}(1 + [\text{p21}]/K_{MCD} + \varepsilon[\text{Plk1P}]/K_{MCD})$, as in Supplementary Table 1. [Cdc20P](0)=0	(18)

$$\frac{1}{\tau}\frac{d[\text{APC/CP:Cdc20}]}{dt} = \mathrm{k_{f10}}[\text{APC/CP}][\text{Cdc20}] - (\mathrm{k_{r8}} + \mathrm{k_{r9}} + \mathrm{k_{r10}})[\text{APC/CP:Cdc20}]$$ [APC/CP:Cdc20](0)=0	(19)
$$\frac{1}{\tau}\frac{d[\text{Cdh1}]}{dt} = \mathrm{k_{s20}} - \mathrm{k_{r11}}[\text{Cdh1}] + \mathrm{k_{f11}}\frac{[\text{Cdh1P}]}{\mathrm{K_{Cdh1P}} + [\text{Cdh1P}]} + \mathrm{k_{r12}}[\text{APC/C:Cdh1}] - \mathrm{k_{f12}}[\text{Cdh1}][\text{APC/C}] + \mathrm{k_{r13}}[\text{APC/CP:Cdh1}] - \mathrm{k_{f13}}[\text{Cdh1}][\text{APC/CP}] - \mathrm{k_{d20}}[\text{Cdh1}]$$ $\mathrm{k_{f11}} = \mathrm{k'_{f11}}[\text{PP2A}]\frac{\mathrm{K_{Pttg11}}}{\mathrm{K_{Pttg11}}+[\text{Pttg1}]}$, $\mathrm{k_{r11}} = \mathrm{k'_{r11}}[\text{MPF}] + \mathrm{k''_{r11}}[\text{Plk1P}]$, as in Supplementary Table 1. [Cdh1](0)=1	(20)
$$\frac{1}{\tau}\frac{d[\text{Cdh1P}]}{dt} = \mathrm{k_{r11}}[\text{Cdh1}] - \mathrm{k_{f11}}\frac{[\text{Cdh1P}]}{\mathrm{K_{Cdh1P}} + [\text{Cdh1P}]} + \mathrm{k_{r11}}[\text{APC/C:Cdh1}] + \mathrm{k_{r11}}[\text{APC/CP:Cdh1}] - \mathrm{k_{d21}}[\text{Cdh1P}]$$ [Cdh1P](0)=0	(21)
$$\frac{1}{\tau}\frac{d[\text{APC/C:Cdh1}]}{dt} = \mathrm{k_{f12}}[\text{APC/C}][\text{Cdh1}] + \mathrm{k_{r8}}[\text{APC/CP:Cdh1}] - (\mathrm{k_{f8}} + \mathrm{k_{r11}} + \mathrm{k_{r12}})[\text{APC/C:Cdh1}]$$ [APC/C:Cdh1](0)=0	(22)
$$\frac{1}{\tau}\frac{d[\text{APC/CP:Cdh1}]}{dt} = \mathrm{k_{f13}}[\text{APC/CP}][\text{Cdh1}] + \mathrm{k_{f8}}[\text{APC/C:Cdh1}] - (\mathrm{k_{r8}} + \mathrm{k_{r11}} + \mathrm{k_{r13}})[\text{APC/CP:Cdh1}]$$ [APC/CP:Cdh1](0)=0	(23)

$\frac{1}{\tau}\frac{d[\mathrm{Pttg1}]}{dt} = \mathrm{k_{s24}} + \mathrm{k_{f14}}\frac{[\mathrm{Pttg1P}]}{\mathrm{K_{Pttg1P1}} + [\mathrm{Pttg1P}]} - \mathrm{k_{r14}}[\mathrm{Pttg1}]$ $- \left(\mathrm{k_{d24.1}} + \mathrm{k_{d24.2}}\frac{[\mathrm{APC/CP{:}Cdc20}]}{\mathrm{K_{Pttg1}} + [\mathrm{Pttg1}]}\right)[\mathrm{Pttg1}]$<br>$\mathrm{k_{f14}} = \mathrm{k'_{f14}}[\mathrm{PP2A}] + \mathrm{k''_{f14}}[\mathrm{Plk1P}],\ \mathrm{k_{r14}} = \mathrm{k'_{r14}}[\mathrm{MPF}]$, as in Supplementary Table 1.<br>[Pttg1](0)=1	(24)
$\frac{1}{\tau}\frac{d[\mathrm{Pttg1P}]}{dt} = \mathrm{k_{r14}}[\mathrm{Pttg1}] - \mathrm{k_{f14}}\frac{[\mathrm{Pttg1P}]}{\mathrm{K_{Pttg1P1}} + [\mathrm{Pttg1P}]}$ $- \left(\mathrm{k_{d25.1}} + \mathrm{k_{d25.2}}\frac{[\mathrm{APC/CP{:}Cdc20}]}{\mathrm{K_{Pttg1P2}} + [\mathrm{Pttg1P}]}\right)[\mathrm{Pttg1P}]$<br>[Pttg1P](0)=0	(25)
$\frac{1}{\tau}\frac{d[\mathrm{LMNAP}]}{dt} = \mathrm{k_{f15}}[\mathrm{LMNA}] - \mathrm{k_{f15}}[\mathrm{LMNAP}]$<br>$\mathrm{k_{f15}} = \mathrm{k'_{f15}}[\mathrm{MPF}],\ \mathrm{LMNA} = \mathrm{LMNAT} - \mathrm{LMNAP},\ \text{where LMNAT} = 1.$<br>[LMNAP](0)=0	(26)
$\frac{1}{\tau}\frac{d[\mathrm{ATM/ATR}]}{dt}$ $= \mathrm{k_{s27}}(1 + \mathrm{K_{DDS}}\mathrm{Sig}) - \frac{\mathrm{k_{d27.1}}[\mathrm{ATM/ATR}][\mathrm{Wip1}]^4}{\mathrm{K_{Wip1}}^4 + [\mathrm{Wip1}]^4} - \mathrm{k_{d27}}[\mathrm{ATM/ATR}]$<br>When $[\mathrm{Mad2{:}Cdc20P}] \geq 0.6$, $\mathrm{DDS} = 200([\mathrm{Mad2{:}Cdc20P}] - 0.6)$<br>When $[\mathrm{Mad2{:}Cdc20P}] \geq 0.36$, $\mathrm{DDS} = 200([\mathrm{Mad2{:}Cdc20P}] - 0.36)$<br>When $[\mathrm{Mad2{:}Cdc20P}] < 0.36$, $\mathrm{DDS} = 0$<br>$\mathrm{Sig} = \mathrm{DDS}\ \mathrm{e}^{-0.00000001\mathrm{t}}$<br>[ATM/ATR](0)=0	(27)
$\frac{1}{\tau}\frac{d[\mathrm{p53}]}{dt} = \mathrm{k_{s28}}(1 + [\mathrm{ATM/ATR}]/\mathrm{K_{A3}}) - \mathrm{k_{f16}}[\mathrm{p53}] + \mathrm{k_{r16}}[\mathrm{p53P}]$ $- \mathrm{k_{d28.1}}[\mathrm{p53}][\mathrm{Mdm2}] - \mathrm{k_{d28}}[\mathrm{p53}]$	(28)

[p53](0)=0	
$\frac{1}{\tau}\frac{d[\text{p53P}]}{dt} = \mathrm{k_{f16}}[\text{p53}] - \mathrm{k_{r16}}[\text{p53P}] + \mathrm{k_{r17}}[\text{p53P:Plk1P}] - \mathrm{k_{f17}}[\text{p53P}][\text{Plk1P}] - \mathrm{k_{d29.1}}[\text{p53P}][\text{Mdm2}] - \mathrm{k_{d29}}[\text{p53P}]$<br>$\mathrm{k_{f16}} = \mathrm{k'_{r16}}\frac{[\text{ATM/ATR}]^4}{\mathrm{K_{A4}}^4+[\text{ATM/ATR}]^4}$, $\mathrm{k_{r16}} = \mathrm{k'_{r16}}[\text{Wip1}]$, as in Supplementary Table 1.<br>[p53P](0)=0	(29)
$\frac{1}{\tau}\frac{d[\text{p53P:Plk1P}]}{dt} = \mathrm{k_{f17}}[\text{p53P}][\text{Plk1P}] - \mathrm{k_{r17}}[\text{p53P:Plk1P}]$<br>[p53P:Plk1P](0)=0	(30)
$\frac{1}{\tau}\frac{d[\text{Mdm2}]}{dt} = \mathrm{k_{s31}} + \mathrm{k_{s31.1}}[\text{p53P}] - \mathrm{k_{d31.1}}[\text{ATM/ATR}][\text{Mdm2}] - \mathrm{k_{d31}}[\text{Mdm2}]$<br>[Mdm2](0)=0	(31)
$\frac{1}{\tau}\frac{d[\text{Wip1}]}{dt} = \mathrm{k_{s32}} + \mathrm{k_{s32.1}}[\text{p53P}] - \mathrm{k_{d32}}[\text{Wip1}]$<br>[Mdm2](0)=0	(32)
$\frac{1}{\tau}\frac{d[\text{Mad2}]}{dt} = \mathrm{k_{s33}} - \mathrm{k_{f18}}[\text{Mad2}][\text{Cdc20P}] + \mathrm{k_{r18}}[\text{Mad2:Cdc20P}] - \mathrm{k_{d33}}[\text{Mad2}]$<br>$\mathrm{k_{r18}} = \mathrm{k'_{r18}}(1 + [\text{p21}]/\mathrm{K_{MCD}} + \varepsilon[\text{Plk1P}]/\mathrm{K_{MCD}})$, as in Supplementary Table 1.<br>[Mad2](0)=1	(33)
$\frac{1}{\tau}\frac{d[\text{Mad2:Cdc20P}]}{dt} = \mathrm{k_{f18}}[\text{Mad2}][\text{Cdc20P}] - \mathrm{k_{r18}}[\text{Mad2:Cdc20P}]$<br>[Mad2:Cdc20P](0)=0	(34)

Supplementary Table 3. Model parameter definitions and values

No	Symbol	Definition	Value
1	k_{f1}	Forward rate constant for association of CycB and CDK1	18
2	k_{r1}	Reverse rate constant for dissociation of MPF	0.1
3	$k_{f2}^{'}$	Dephosphorylation rate constant of preMPF by Cdc25P	10
4	$k_{f2}^{''}$	Dephosphorylation rate constant of preMPF by Cdc25	0.2
5	$k_{r2}^{'}$	Phosphorylation rate constant of MPF by Wee1	3
6	$k_{r2}^{''}$	Phosphorylation rate constant of MPF by Wee1P	0.1
7	$k_{f3}^{'}$	Phosphorylation rate constant of Cdc25 by MPF	1
8	$k_{f3}^{''}$	Phosphorylation rate constant of Cdc25 by Plk1P	2
9	$k_{r3}^{'}$	Dephosphorylation rate constant of Cdc25P by PP2A	0.54
10	$k_{f4}^{'}$	Phosphorylation rate constant of Wee1 by MPF	1
11	$k_{f4}^{''}$	Phosphorylation rate constant of Wee1 by Plk1P	2
12	$k_{r4}^{'}$	Dephosphorylation rate constant of Wee1 by PP2A	0.2
13	k_{f5}	Forward rate constant for dissociation of p21:MPF	8
14	k_{r5}	Reverse rate constant for association of p21 and MPF	80
15	$k_{f6}^{'}$	Phosphorylation rate constant of Plk1 by MPF	5
16	$k_{r6}^{'}$	Dephosphorylation rate constant of Plk1P by PP2A	0.5
17	k_{f7}	Dephosphorylation rate constant of PP2AP	0.4
18	k_{r7}	Phosphorylation rate constant of PP2A by MPF	4
19	$k_{f8}^{'}$	Phosphorylation rate constant of APC/C by MPF	0.1
20	k'_{f8}	Phosphorylation rate constant of APC/C by Plk1P	0.35
21	$k_{r8}^{'}$	Dephosphorylation rate constant of APC/CP by PP2A	0.04

22	$k_{r9}^{'}$	Phosphorylation rate constant of Cdc20 by MPF	11
23	$k'^{'}_{r9}$	Phosphorylation rate constant of Cdc20 by Plk1P	11
24	$k_{f9}^{'}$	Dephosphorylation rate constant of Cdc20 by PP2A	20
25	k_{f10}	Forward rate constant for association of APC/CP and Cdc20	80
26	k_{r10}	Reverse rate constant for dissociation of APC/CP:Cdc20	20
27	$k_{f11}^{'}$	Dephosphorylation rate constant of Cdh1P by PP2A	0.02
28	$k_{r11}^{'}$	Phosphorylation rate constant of Cdh1 by MPF	22
29	$k_{r11}^{''}$	Phosphorylation rate constant of Cdh1 by Plk1P	22
30	k_{f12}	Forward rate constant for association of APC/C and Cdh1	80
31	k_{r12}	Reverse rate constant for dissociation of APC/C:Cdh1	20
32	k_{f13}	Forward rate constant for association of APC/CP and Cdh1	80
33	k_{r13}	Reverse rate constant for dissociation of APC/CP:Cdh1	3
34	$k_{f14}^{'}$	Dephosphorylation rate constant of Pttg1P by PP2A	0.01
35	$k_{r14}^{'}$	Phosphorylation rate constant of Pttg1 by MPF	50
36	$k_{f15}^{'}$	Phosphorylation rate constant of LMNA by MPF	1
37	k_{r15}	Dephosphorylation rate constant of LMNAP	1
38	$k_{f16}^{'}$	Phosphorylation rate constant of p53 by ATM/ATR	1
39	$k_{r16}^{'}$	Dephosphorylation rate constant of p53P by Wip1	3
40	k_{f17}	Forward rate constant for association of Plk1P and p53P	80
41	k_{r17}	Reverse rate constant for association of Plk1P:p53P	20
42	k_{f18}	Forward rate constant for association of Mad2 and Cdc20P	20
43	k'_{r18}	Reverse rate constant for dissociation of Mad2:Cdc20P	0.1
44	k_{s1}	CycB synthesis rate constant	0.1
45	k_{s5}	p21 synthesis rate constant influenced by p53	0.0001
46	k_{s8}	Cdc25 synthesis rate constant	0.1
47	k_{s9}	Wee1 synthesis rate constant	0.1

48	k_{s12}	Plk1 synthesis rate constant	0.1
49	k_{s13}	PP2A synthesis rate constant	0.1
50	k_{s15}	APC/C synthesis rate constant	0.1
51	k_{s17}	Cdc20 synthesis rate constant	0.1
52	k_{s20}	Cdh1 synthesis rate constant	0.1
53	k_{s24}	Pttg1 synthesis rate constant	0.1
54	k_{s27}	ATM/ATR synthesis rate constant influenced by DDS	0.0001
55	k_{s28}	p53 synthesis rate constant influenced by ATM/ATR	0.0001
56	k_{s31}	Mdm2 synthesis rate constant	0.0001
57	$k_{s31.1}$	p53P-dependent Mdm2 synthesis rate constant	6
58	k_{s32}	Wip1 synthesis rate constant	0.0001
59	$k_{s32.1}$	p53P-dependent Wip1 synthesis rate constant	6
60	k_{s33}	Mad2 synthesis rate constant	0.1
61	$k_{d1.1}$	CycB self-degradation rate constant	0.3
62	$k_{d1.2}$	CycB degradation rate constant by APC/CP:Cdc20	1
63	$k_{d1.3}$	CycB degradation rate constant by APC/CT:Cdh1	1
64	$k_{d3.1}$	MPF self-degradation rate constant	0.01
65	$k_{d3.2}$	MPF degradation rate constant by APC/CP:Cdc20	1
66	$k_{d3.3}$	MPF degradation rate constant by APC/CT:Cdh1	1
67	$k_{d4.1}$	preMPF self-degradation rate constant	0.01
68	$k_{d4.2}$	preMPF degradation rate constant by APC/CP:Cdc20	1
69	$k_{d4.3}$	preMPF degradation rate constant by APC/CT:Cdh1	1
70	k_{d5}	p21 self-degradation rate constant	0.01
71	k_{d6}	p21 degradation rate constant in p21:MPF by APC/CP:Cdc20	1
72	$k_{d7.1}$	Cdc25P self-degradation rate constant	0.18
73	$k_{d7.3}$	Cdc25P degradation rate constant by APC/CT:Cdh1	0.1
74	$k_{d8.1}$	Cdc25 self-degradation rate constant	0.08
75	$k_{d8.3}$	Cdc25 degradation rate constant by APC/CT:Cdh1	1
76	k_{d9}	Wee1 self-degradation rate constant	0.1
77	k_{d10}	Wee1P self-degradation rate constant	1
78	$k_{d11.1}$	Plk1P self-degradation rate constant	0.1
79	$k_{d11.3}$	Plk1P degradation rate constant by APC/CT:Cdh1	1.5

80	$k_{d12.1}$	Plk1 self-degradation rate constant	0.2
81	$k_{d12.3}$	Plk1 degradation rate constant by APC/CT:Cdh1	1.5
82	k_{d13}	PP2A self-degradation rate constant	0.1
83	k_{d14}	PP2AP self-degradation rate constant	0.1
84	k_{d15}	APC/C self-degradation rate constant	0.2
85	k_{d16}	APC/CP self-degradation rate constant	0.1
86	$k_{d17.1}$	Cdc20 self-degradation rate constant	0.2
87	$k_{d17.3}$	Cdc20 degradation rate constant by APC/CT:Cdh1	1.5
88	$k_{d18.1}$	Cdc20P self-degradation rate constant	0.2
89	$k_{d18.3}$	Cdc20P degradation rate constant by APC/CT:Cdh1	1.5
90	k_{d20}	Cdh1 self-degradation rate constant	0.2
91	k_{d21}	Cdh1P self-degradation rate constant	0.2
92	$k_{d24.1}$	Pttg1 self-degradation rate constant	0.1
93	$k_{d24.2}$	Pttg1 degradation rate constant by APC/CP:Cdc20	1
94	$k_{d25.1}$	Pttg1P self-degradation rate constant	0.1
95	$k_{d25.2}$	Pttg1P degradation rate constant by APC/CP:Cdc20	1
96	k_{d27}	ATM/ATR self-degradation rate constant	0.1
97	$k_{d27.1}$	Saturating Wip1-dependent ATM/ATR degradation rate	100
98	k_{d28}	p53 self-degradation rate constant	0.2
99	$k_{d28.1}$	Mdm2-dependent p53 degradation rate	1
100	k_{d28}	p53P degradation rate constant	0.2
101	$k_{d29.1}$	Mdm2-dependent p53P degradation rate	1
102	$k_{d31.1}$	ATM/ATR dependent Mdm2 inactivation rate	1
103	k_{d31}	Mdm2 degradation rate constant	0.2
104	k_{d32}	Wip1 degradation rate constant	5
105	k_{d33}	Mad2 degradation rate constant	0.2
106	K_{A1}	Intensifying scale rate of k'_{f6} by ATM_ATR	1
107	K_{Pttg11}	Intensifying scale rate of k'_{f11} by Pttg1	0.5
108	K_{A2}	Intensifying scale rate of k_{s8} by ATM/ATR	1
109	K_{p53}	Intensifying scale rate of ks5 by p53	0.001
110	K_{DDS}	Intensifying scale rate of DDS	1000
111	K_{A3}	Intensifying scale rate of ks28 by ATM_ATR	0.0002

112	K_{MCD}	Intensifying scale rate of Mad2_Cdc20P dissociation by p21	0.01
113	ε	Intensifying scale ratio of Plk1/p21	1
114	K_{CycB1}	MM constant of CycB degradation by APC/CP:Cdc20	0.1
115	K_{CycB2}	MM constant of CycB degradation by APC/CP:Cdh1	0.1
116	K_{MPF1}	MM constant of MPF degradation by APC/CP:Cdc20	0.1
117	K_{MPF2}	MM constant of MPF degradation by APC/CT:Cdh1	0.1
118	$K_{preMPF1}$	MM constant of MPF degradation by APC/CP:Cdc20	0.1
119	$K_{preMPF2}$	MM constant of MPF degradation by APC/CP:Cdh1	0.1
120	K_{p21MPF}	MM constant of p21:MPF degradation by APC/CP:Cdc20	0.1
121	$K_{Cdc25P1}$	MM constant of Cdc25CP Dephosphorylation by PP2A	0.1
122	$K_{Cdc25P2}$	MM constant of Cdc25P degradation by APC/CT:Cdh1	0.1
123	K_{Cdc25}	MM constant of Cdc25 degradation by APC/CT:Cdh1	0.1
124	K_{Wee1P}	MM constant of Wee1P dephosphorylation by PP2A	0.1
125	K_{Plk1P1}	MM constant of Plk1P dephosphorylation by PP2A	0.1
126	K_{Plk1P2}	MM constant of Plk1P degradation by APC/CT:Cdh1	0.1
127	K_{Plk1}	MM constant of Plk1 degradation by APC/CT:Cdh1	0.1
128	$K_{APC/CP}$	MM constant of APC/CP dephosphorylation by PP2A	0.1
129	$K_{Cdc20P1}$	MM constant of Cdc20P dephosphorylation by PP2A	0.1
130	K_{Cdc20}	MM constant of Cdc20 degradation by APC/CT:Cdh1	0.1
131	$K_{Cdc20P2}$	MM constant of Cdc20P degradation by APC/CT:Cdh1	0.1
132	K_{Cdh1P}	MM constant of Cdh1P dephosphorylation by PP2A	0.1
133	$K_{Pttg1P1}$	MM constant of Pttg1P dephosphorylation by PP2A	0.1
134	K_{Pttg1}	MM constant of Pttg1 degradation by APC/CP:Cdc20	0.1
135	$K_{Pttg1P2}$	MM constant of Pttg1P degradation by APC/CP:Cdc20	0.1
136	K_{A4}	MM constant of p53 phosphorylation by Wip1	0.5
137	K_{Wip1}	MM constant of ATM/ATR signal degradation by Wip1	0.5

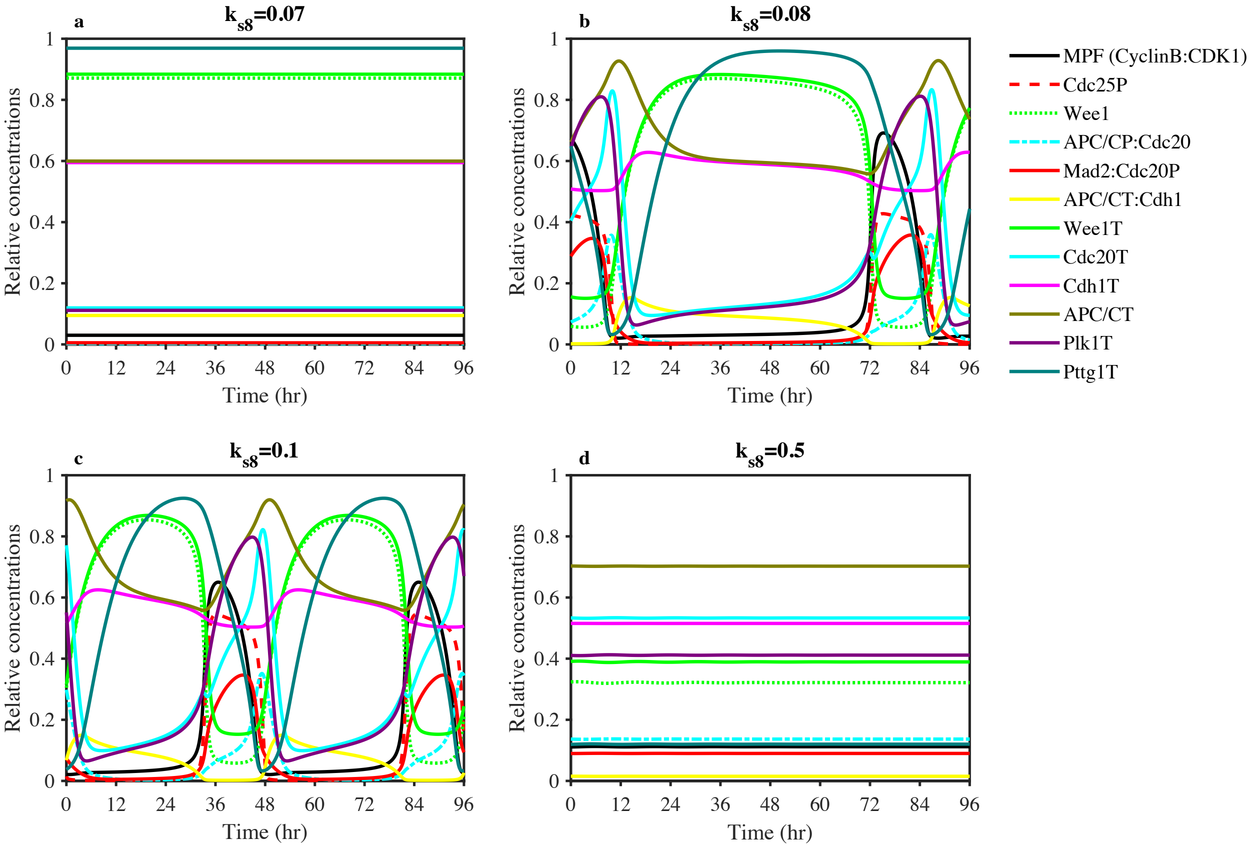

Supplementary Figure 1. Numerical simulation results obtained using four different k_{s8} parameter values: **(a)** 0.07, **(b)** 0.08, **(c)** 0.1, and **(d)** 0.5. Dynamics of cell cycle components shows that limit cycle oscillations exist when k_{s8} is between 0.08 and 0.1 but not for values less than or equal to 0.07 and greater than or equal to 0.5.

Supplementary Table 4. Parameter ranges for the model to exhibit limit cycle oscillations

No	Symbol	Value	Left endpoint	Right endpoint
1	k_{f1}	18	10	191
2	k_{r1}	0.1	0	3.1
3	k'_{f2}	10	0.3	120
4	k''_{f2}	0.2	0.1	2
5	k'_{r2}	3	1	3
6	k''_{r2}	0.1	0	26
7	k'_{f3}	1	0	9
8	k''_{f3}	2	0	49
9	k'_{r3}	0.54	0.07	>1000
10	k'_{f4}	1	0	15
11	k''_{f4}	2	0	25
12	k'_{r4}	0.2	0	3.4
13	k_{f5}	8	0	>1000
14	k_{r5}	80	0	>1000
15	k'_{f6}	5	1	14
16	k'_{r6}	0.5	0.1	16.4
17	k_{f7}	0.4	0.2	>1000
18	k'_{r7}	4	0	6
19	k'_{f8}	0.1	0	0.4
20	k''_{f8}	0.35	0.05	1.9
21	k'_{r8}	0.04	0.01	0.13
22	k'_{r9}	11	0	>1000
23	k''_{r9}	11	0	>1000
24	k'_{f9}	20	0	>1000
25	k_{f10}	80	0	400
26	k_{r10}	20	4	58

27	k'_{f11}	0.02	0	0.03
28	k'_{r11}	22	20	>1000
29	k''_{r11}	22	0	>1000
30	k_{f12}	80	0	104
31	k_{r12}	20	16	>1000
32	k_{f13}	80	0	848
33	k_{r13}	3	0	>1000
34	k'_{f14}	0.01	0	>1000
35	k'_{r14}	50	0	>1000
36	k'_{f15}	1	0	>1000
37	k_{r15}	1	0	>1000
38	k'_{f16}	1	0	>1000
39	k'_{r16}	3	0	>1000
40	k_{f17}	80	0	>1000
41	k_{r17}	20	0	>1000
42	k_{f18}	20	0	>1000
43	k'_{r18}	0.1	0	>1000
44	k_{s1}	0.1	0.1	0.1
45	k_{s5}	0.0001	0	0.0073
46	k_{s8}	0.1	0.1	0.3
47	k_{s9}	0.1	0.1	0.1
48	k_{s12}	0.1	0.1	0.1
49	k_{s13}	0.1	0	0.1
50	k_{s15}	0.1	0	0.1
51	k_{s17}	0.1	0.1	0.2
52	k_{s20}	0.1	0	0.1
53	k_{s24}	0.1	0	>1000
54	k_{s27}	0.0001	0	10
55	k_{s28}	0.0001	0	0.00981

56	k_{s31}	0.0001	0	>1000
57	k_{s31p}	6	0	>1000
58	k_{s32}	0.0001	0	10
59	k_{s32p}	6	0	>1000
60	k_{s33}	0.1	0	>1000
61	$k_{d1.1}$	0.3	0	1.4
62	$k_{d1.2}$	1	0	49
63	$k_{d1.3}$	1	0	2
64	$k_{d3.1}$	0.01	0	0.23
65	$k_{d3.2}$	1	1	8
66	$k_{d3.3}$	1	0	1
67	$k_{d4.1}$	0.01	0	0.16
68	$k_{d4.2}$	1	0	4
69	$k_{d4.3}$	1	0	1
70	k_{d5}	0.01	0	>1000
71	k_{d6}	1	0	>1000
72	$k_{d7.1}$	0.18	0	>1000
73	$k_{d7.3}$	0.1	0	>1000
74	$k_{d8.1}$	0.08	0	0.24
75	$k_{d8.3}$	1	0	1
76	k_{d9}	0.1	0.1	0.5
77	k_{d10}	1	1	>1000
78	$k_{d11.1}$	0.1	0	92.7
79	$k_{d11.3}$	1.5	0	96.5
80	$k_{d12.1}$	0.2	0	18.5
81	$k_{d12.3}$	1.5	0	21.2
82	k_{d13}	0.1	0	0.4
83	k_{d14}	0.1	0	0.2
84	k_{d15}	0.2	3	>1000

85	k_{d16}	0.1	0	>1000
86	$k_{d17.1}$	0.2	0	0.9
87	$k_{d17.3}$	1.5	0	14
88	$k_{d18.1}$	0.2	0	0.6
89	$k_{d18.3}$	1.5	0	48.6
90	k_{d20}	0.2	0.1	>1000
91	k_{d21}	0.2	0	>1000
92	$k_{d24.1}$	0.1	0	>1000
93	$k_{d24.2}$	1	0	>1000
94	$k_{d25.1}$	0.1	0	>1000
95	$k_{d25.2}$	1	0	>1000
96	k_{d27}	0.1	0	>1000
97	k_{d27p}	100	0	>1000
98	k_{d28}	0.2	0	>1000
99	k_{d28p}	1	0	>1000
100	k_{d29}	0.2	0	>1000
101	k_{d29p}	1	0	>1000
102	$k_{d31.1}$	1	0	>1000
103	k_{d31}	0.2	0	>1000
104	k_{d32}	5	0	>1000
105	k_{d33}	0.2	0	>1000
106	K_{A1}	1	1	>1000
107	K_{Pttg1}	0.5	0	>1000
108	K_{A2}	1	1	>1000
109	K_{p53}	0.001	0.001	100
110	K_{DDS}	1000	0	>1000
111	K_{A3}	0.0002	0.0001	>1000
112	K_{MCD}	0.01	0.01	>1000
113	ε	1	0	>1000

114	K_{CycB1}	0.1	0.1	>1000
115	K_{CycB2}	0.1	0.1	>1000
116	K_{MPF1}	0.1	0.1	0.9
117	K_{MPF2}	0.1	0.1	>1000
118	$K_{preMPF1}$	0.1	0.1	>1000
119	$K_{preMPF2}$	0.1	0.1	>1000
120	K_{p21MPF}	0.1	0.1	>1000
121	$K_{Cdc25P1}$	0.1	0.1	1.2
122	$K_{Cdc25P2}$	0.1	0.1	>1000
123	K_{Cdc25}	0.1	0.1	>1000
124	K_{Wee1P}	0.1	0.1	>1000
125	K_{Plk1P1}	0.1	0.1	0.4
126	K_{Plk1P2}	0.1	0.1	>1000
127	K_{Plk1}	0.1	0.1	>1000
128	$K_{APC/CP}$	0.1	0.1	>1000
129	$K_{Cdc20P1}$	0.1	0.1	0.4
130	K_{Cdc20}	0.1	0.1	>1000
131	$K_{Cdc20P2}$	0.1	0.1	>1000
132	K_{Cdh1P}	0.1	0.1	>1000
133	$K_{Pttg1P1}$	0.1	0.1	>1000
134	K_{Pttg1}	0.1	0	>1000
135	$K_{Pttg1P2}$	0.1	0.1	>1000
136	K_{A4}	0.5	0.1	>1000
137	K_{Wip1}	0.5	0.1	>1000

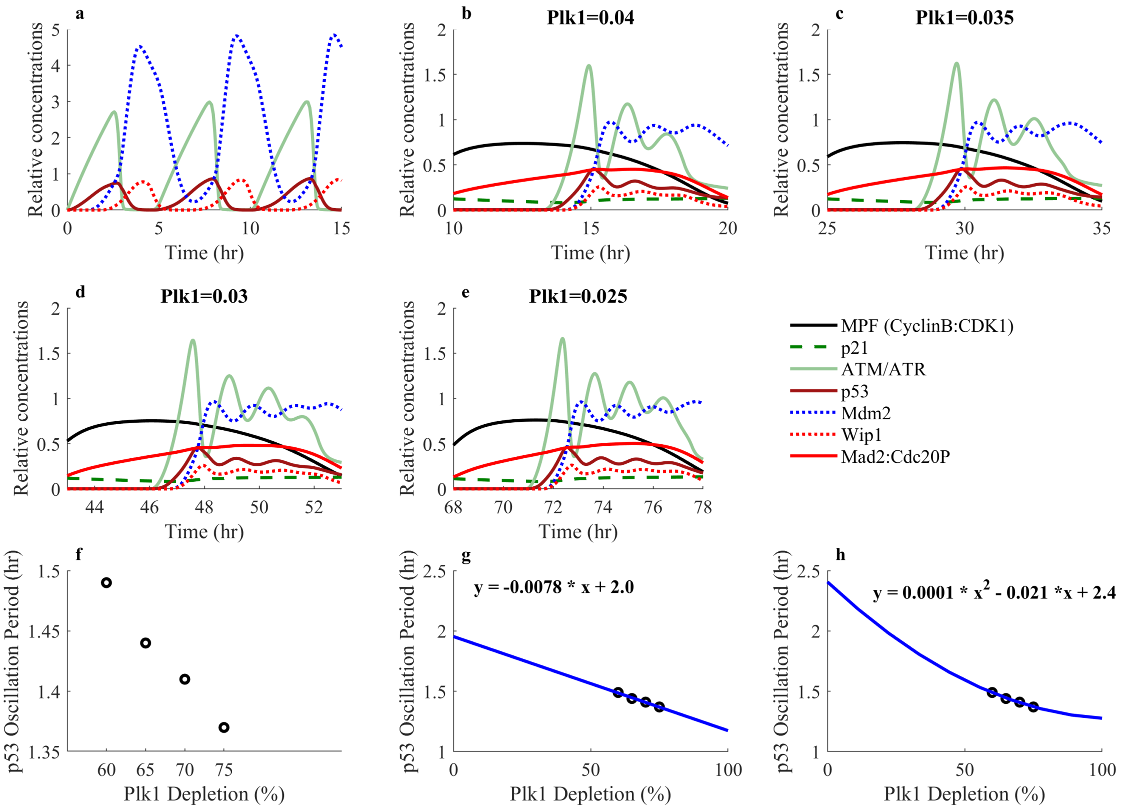

Supplementary Figure 2. The period of oscillations of ATM/ATR, p53, Wip1, Mdm2 regulators. (**a**) When ATM/ATR-p53-Wip1-Mdm2 subnetwork is disconnected from the cell cycle regulation it produces oscillations with a period of ~5hrs. (**b-f**) Oscillations in p53 regulation that is connected with the cell cycle regulation (our full model). The period of oscillations depends on the level of Plk1 depletion that induces the activation of DNA damage checkpoint. (**f**) The dependence of the period of p53 oscillations on the level of Plk1 depletion. (**g-h**) Fitting and extrapolation the dependence of p53 oscillation period on Plk1 depletion level using a linear and polynomial functions. Oscillations can be resolved only at high level of Plk1 depletion, when DNA damage checkpoint is activated. The extrapolation to lower Plk1 depletion values shows the expected period of oscillations at lower levels of Plk1 depletion.

Supplementary Table 5. The number of different cancer cell lines that carry a specific gene mutation from Dependency Map database (depmap.org) and the number of cell lines for which CRISPR data are available.

Gene	Number of different cancer cell lines that carry a corresponding gene mutation	Number of different cancer cell lines that carry a corresponding gene mutation and for which CRISPR data are available
TP53	1091	549
ATM	267	136
ATR	184	92
PPM1D	78	28
FZR1	74	34
CDC25B	62	24
CDC27	59	29
PLK1	58	31
LMNA	51	26
CDC16	48	20
CDC23	39	20
CDC25A	39	15
CDKN1A	39	23
CDC25C	37	14
CDC20	35	18
MDM2	34	20
WEE1	34	17
CCNB1	27	14
CCNB2	24	9
PPP2CA	23	11
PTTG1	20	14
MAD2L1	18	6
MAD2L2	18	9
ATM-TP53	190	106
ATR-TP53	128	63
FZR1-TP53	56	23
PPM1D-TP53	56	21
ATM-ATR	53	29
CDC25B-TP53	43	18
CDC27-TP53	41	21
ATM-FZR1	39	20
PLK1-TP53	39	24
LMNA-TP53	34	16
CDC16-TP53	33	17
CDC23-TP53	31	16

CDKN1A-TP53	29	17
CDC20-TP53	28	15
MDM2-TP53	28	15
ATM-PPM1D	27	9
CDC25A-TP53	27	12
ATM-CDC25B	23	9
CDC25C-TP53	22	8
ATR-FZR1	21	9
TP53-WEE1	21	12
ATM-CDC23	20	8
ATR-PPM1D	20	6
ATM-CDC27	19	11
CCNB2-TP53	19	9
ATM-PLK1	18	9
ATR-CDC25B	17	7
PTTG1-TP53	17	13
ATM-CDC25A	16	6
ATR-LMNA	16	5
ATM-CDC16	15	6
ATM-CDC20	15	8
ATM-LMNA	15	7
ATM-MDM2	15	5
ATR-CDC23	15	6
FZR1-PLK1	15	9
PPP2CA-TP53	15	7
ATR-CDC27	14	8
CCNB1-TP53	14	8
CDC25A-FZR1	14	5
ATM-CDC25C	13	4
ATR-PLK1	13	8
MAD2L1-TP53	13	5
ATM-CCNB2	12	4
ATM-CDKN1A	12	7
ATM-WEE1	12	7
ATR-CDC25A	12	4
ATR-CDC25C	12	3
ATR-CDKN1A	12	6
ATR-MDM2	12	4
CDC25B-FZR1	12	3
FZR1-PPM1D	12	3
MAD2L2-TP53	12	7

ATM-PPP2CA	11	2
ATM-PTTG1	11	9
ATR-CDC16	11	3
ATR-WEE1	11	5
CDC20-FZR1	11	5
CDC25C-PPM1D	10	1
CDC27-FZR1	10	5
ATR-CDC20	9	5
CDC16-FZR1	9	4
CDC23-FZR1	9	1
CDC25A-LMNA	9	4
CDC27-PLK1	9	6
MDM2-PPM1D	9	2
ATM-MAD2L1	8	3
ATR-CCNB1	8	5
ATR-PPP2CA	8	4
CDC20-WEE1	8	5
CDC25A-CDC25B	8	1
CDC25B-PLK1	8	5
CDKN1A-FZR1	8	4
FZR1-LMNA	8	2
ATM-MAD2L2	7	4
ATR-MAD2L2	7	2
CDC20-CDC25B	7	3
CDC20-PPM1D	7	2
CDC25A-CDC27	7	3
CDC25B-CDC27	7	3
CDC25B-WEE1	7	3
CDC25C-CDKN1A	7	4
CDC25C-LMNA	7	2
CDKN1A-PPM1D	7	3
FZR1-PTTG1	7	5
FZR1-WEE1	7	3
MDM2-PLK1	7	4
PLK1-PPM1D	7	1
PLK1-PTTG1	7	6
CDC16-CDC25A	6	2
CDC16-LMNA	6	2
CDC16-PLK1	6	3
CDC16-WEE1	6	2
CDC20-CDC25A	6	2

CDC20-CDC27	6	2
CDC23-CDC27	6	3
CDC25A-CDC25C	6	2
CDC25A-MDM2	6	1
CDC25B-MDM2	6	3
CDC27-WEE1	6	3
FZR1-MDM2	6	2
LMNA-PPM1D	6	2
ATM-CCNB1	5	3
ATR-CCNB2	5	2
ATR-MAD2L1	5	2
ATR-PTTG1	5	4
CDC16-CDC20	5	3
CDC16-MAD2L2	5	2
CDC23-MDM2	5	2
CDC23-PPM1D	5	1
CDC25A-CDKN1A	5	2
CDC25A-MAD2L2	5	2
CDC25A-PLK1	5	2
CDC25A-PPM1D	5	0
CDC25A-WEE1	5	2
CDC25B-MAD2L1	5	1
CDC25C-FZR1	5	2
CDC25C-MDM2	5	1
CDKN1A-MDM2	5	1
CDKN1A-PLK1	5	1
FZR1-MAD2L2	5	4
FZR1-PPP2CA	5	1
LMNA-MDM2	5	1
LMNA-PLK1	5	3
MAD2L2-PPM1D	5	1
PPM1D-PPP2CA	5	1
CCNB1-CDC25A	4	2
CCNB2-FZR1	4	2
CDC16-CDC23	4	1
CDC16-CDC27	4	1
CDC16-PPM1D	4	0
CDC16-PPP2CA	4	2
CDC20-CDKN1A	4	2
CDC20-LMNA	4	1
CDC20-MAD2L2	4	2

CDC20-PLK1	4	2
CDC23-CDC25A	4	0
CDC23-LMNA	4	0
CDC23-PLK1	4	2
CDC23-WEE1	4	2
CDC25A-MAD2L1	4	2
CDC25B-PPM1D	4	1
CDC25C-CDC27	4	3
CDC27-MDM2	4	2
CDC27-PPP2CA	4	1
CDKN1A-LMNA	4	1
LMNA-PPP2CA	4	1
MAD2L1-PLK1	4	2
MAD2L2-WEE1	4	2
CCNB1-CDC25B	3	2
CCNB1-CDC25C	3	1
CCNB1-LMNA	3	1
CCNB1-MAD2L2	3	1
CCNB2-CDC25A	3	0
CCNB2-CDC25B	3	1
CCNB2-PLK1	3	3
CDC16-CDC25B	3	1
CDC16-CDKN1A	3	2
CDC16-MAD2L1	3	1
CDC16-MDM2	3	0
CDC16-PTTG1	3	2
CDC20-CDC23	3	1
CDC20-MAD2L1	3	1
CDC20-PTTG1	3	2
CDC23-CDC25B	3	1
CDC23-CDKN1A	3	1
CDC23-PPP2CA	3	0
CDC25A-PPP2CA	3	2
CDC25B-CDC25C	3	1
CDC25B-CDKN1A	3	1
CDC25B-LMNA	3	1
CDC25C-MAD2L2	3	0
CDC27-LMNA	3	1
CDC27-PPM1D	3	1
CDC27-PTTG1	3	2
CDKN1A-PPP2CA	3	3

CDKN1A-PTTG1	3	2
FZR1-MAD2L1	3	1
LMNA-MAD2L2	3	1
LMNA-WEE1	3	1
MAD2L1-PPM1D	3	1
MAD2L2-PLK1	3	3
MDM2-WEE1	3	1
PPM1D-WEE1	3	0
CCNB1-CDC20	2	1
CCNB1-CDC27	2	2
CCNB1-FZR1	2	2
CCNB1-MAD2L1	2	1
CCNB1-PPM1D	2	0
CCNB2-CDC16	2	1
CCNB2-CDC20	2	1
CCNB2-CDC23	2	0
CCNB2-MAD2L2	2	1
CCNB2-PTTG1	2	2
CDC16-CDC25C	2	0
CDC20-CDC25C	2	1
CDC20-MDM2	2	0
CDC23-MAD2L2	2	1
CDC23-PTTG1	2	2
CDC25B-MAD2L2	2	1
CDC25B-PTTG1	2	2
CDC25C-PLK1	2	0
CDC25C-WEE1	2	1
CDC27-CDKN1A	2	1
CDC27-MAD2L1	2	2
CDC27-MAD2L2	2	2
MAD2L1-MAD2L2	2	1
MAD2L1-PPP2CA	2	2
PLK1-PPP2CA	2	0
PLK1-WEE1	2	2
PPM1D-PTTG1	2	1
PTTG1-WEE1	2	2
CCNB1-CCNB2	1	0
CCNB1-CDC16	1	0
CCNB1-CDKN1A	1	1
CCNB1-PLK1	1	0
CCNB1-PPP2CA	1	1

CCNB1-WEE1	1	1
CCNB2-CDC25C	1	0
CCNB2-CDKN1A	1	1
CCNB2-MAD2L1	1	0
CCNB2-MDM2	1	0
CCNB2-PPM1D	1	0
CCNB2-PPP2CA	1	0
CDC20-PPP2CA	1	1
CDC23-CDC25C	1	0
CDC23-MAD2L1	1	0
CDC25C-MAD2L1	1	0
CDC25C-PPP2CA	1	0
CDC25C-PTTG1	1	1
CDKN1A-MAD2L1	1	0
CDKN1A-MAD2L2	1	0
CDKN1A-WEE1	1	0
LMNA-MAD2L1	1	0
LMNA-PTTG1	1	1
MAD2L1-MDM2	1	0
MAD2L1-WEE1	1	1
MAD2L2-MDM2	1	0
MDM2-PPP2CA	1	0
MDM2-PTTG1	1	1
PPP2CA-WEE1	1	0

Supplementary Table 6. The average logarithmic sensitivity intensities for p53-wt, p53-wt Plk1 depleted, p53-null cancer and Plk1-depleted p53-null cancer cells.

p53-wt cancer cells		Plk1 depleted to 0.055 level in p53-wt cells		p53-null cells		Plk1 depleted to 0.055 level in p53-null cells	
Protein	Intensity	Protein	Intensity	Protein	Intensity	Protein	Intensity
Cdc20P	0.88	p53P	4.29	ATM/ATR	19.97	Cdc20P	6.37
APC/CP:Cdc20	0.87	Wip1	3.29	APC/CP:Cdc20	0.89	Plk1P	5.38
p53P:Plk1P	0.86	p53	3.2	Cdc20P	0.87	APC/CP	5.11
Plk1P	0.72	p53P:Plk1P	3.14	APC/CP	0.71	Cdc25P	4.78
APC/CP	0.71	p53T	2.96	Plk1P	0.7	ATM/ATR	4.57
Mad2:Cdc20P	0.7	p21:MPF	2.9	Mad2:Cdc20P	0.64	APC/CP:Cdc20	4.28
APC/CP:Cdh1	0.6	ATM/ATR	2.73	p21:MPF	0.58	Mad2:Cdc20P	4.19
Cdc25P	0.58	Mdm2	2.71	Cdc25P	0.56	p21:MPF	3.84
MPF (Cy-clinB:CDK1)	0.53	APC/CP:Cdh1	2.68	APC/CP:Cdh1	0.54	MPF (Cy-clinB:CDK1)	3.53
p21:MPF	0.5	APC/CP:Cdc20	1.52	MPF (Cy-clinB:CDK1)	0.53	LMNAP	3.07
Plk1	0.49	preMPF	1.49	Plk1	0.49	Plk1T	2.43
LMNAP	0.47	APC/CP	1.21	LMNAP	0.47	preMPF	1.63
Cdc20T	0.45	CyclinBT	1.06	Cdc20T	0.46	Wee1P	1.63
preMPF	0.44	p21T	1	preMPF	0.43	Cdc25	1.59
Pttg1P	0.44	p21	0.95	Plk1T	0.43	APC/CP:Cdh1	1.42
Pttg1T	0.43	APC/CPT	0.83	APC/C:Cdh1	0.43	PP2AP	1.39
Plk1T	0.42	Cdc25	0.81	Pttg1P	0.43	Mdm2	1.37
APC/C:Cdh1	0.42	Cdc20T	0.78	Pttg1T	0.42	CyclinB	1.33
Cdh1	0.39	Mad2:Cdc20P	0.78	Cdh1	0.39	Cdc20T	1.26
Cdc20	0.39	Pttg1P	0.74	Cdc20	0.39	Wee1	0.96
APC/CPT	0.37	Cdc25T	0.71	APC/CPT	0.36	Wee1T	0.85
Pttg1	0.32	APC/CT:Cdh1	0.7	Pttg1	0.33	APC/C:Cdh1	0.82
Wee1	0.32	Pttg1T	0.67	Wee1	0.33	CyclinBT	0.78
p53P	0.3	Wee1P	0.67	Cdc25	0.29	Plk1	0.77
Wee1P	0.29	Plk1T	0.53	Wee1T	0.28	Cdh1	0.77
Wee1T	0.28	CyclinB	0.51	Wee1P	0.28	Pttg1	0.74
Cdc25	0.27	Cdc20P	0.5	CyclinBT	0.27	PP2A	0.68
CyclinBT	0.27	Plk1	0.49	CyclinB	0.26	APC/CT:Cdh1	0.64
PP2AP	0.26	Pttg1	0.48	PP2AP	0.26	Pttg1T	0.62
CyclinB	0.26	MPF (Cy-clinB:CDK1)	0.46	APC/CT:Cdh1	0.21	Cdc20	0.61
APC/CT:Cdh1	0.2	Plk1P	0.45	Cdc25T	0.2	Pttg1P	0.61
Cdc25T	0.19	Cdc20	0.43	PP2A	0.17	Mad2T	0.56
PP2A	0.17	APC/C:Cdh1	0.43	Mad2T	0.13	Cdc25T	0.52
Mad2T	0.13	CDK1	0.42	APC/C	0.12	APC/CPT	0.43
p21	0.11	LMNAP	0.35	CDK1	0.11	APC/C	0.34

p21T	0.11	Cdh1	0.34	APC/CT	0.1	CDK1	0.33
APC/C	0.11	PP2AP	0.31	Mdm2	0.08	Mad2	0.24
CDK1	0.11	Cdc25P	0.3	Mad2	0.06	APC/CT	0.14
APC/CT	0.1	APC/CT	0.23	Cdh1T	0.05	Cdh1T	0.12
Mad2	0.06	Wee1T	0.17	Cdh1P	0.03	Cdh1P	0.06
Cdh1T	0.05	Wee1	0.16	p21	0.03	p21	0.03
Cdh1P	0.03	PP2A	0.14	p21T	0.03	p21T	0.03
p53T	0.03	Mad2T	0.13	PP2AT	0.01	Wip1	0.01
p53	0.03	Mad2	0.1	Wip1	0.01	PP2AT	0.01
Mdm2	0.01	Cdh1T	0.08	CDK1T	0	CDK1T	0
PP2AT	0.01	APC/C	0.08	LMNAT	0	LMNAT	0
ATM/ATR	0.01	Cdh1P	0.07	p53	NaN	p53	NaN
Wip1	0.01	PP2AT	0.01	p53P	NaN	p53P	NaN
CDK1T	0	CDK1T	0	p53P:Plk1P	NaN	p53P:Plk1P	NaN
LMNAT	0	LMNAT	0	p53T	NaN	p53T	NaN

Supplementary Table 7. Twenty proteins with largest average logarithmic sensitivity intensities for p53-wt cells, p53-wt Plk1 depleted cells, p53-null cancer and Plk1-depleted p53-null cancer cells.

p53-wt cancer cells		Plk1 depleted to 0.055 level in p53-wt cancer cells		p53-null cells		Plk1 depleted to 0.055 level in p53-null cells	
Protein	Intensity	Protein	Intensity	Protein	Intensity	Protein	Intensity
Cdc20P	0.88	p53P	4.29	ATM/ATR	19.97	Cdc20P	6.37
APC/CP:Cdc20	0.87	Wip1	3.29	APC/CP:Cdc20	0.89	Plk1P	5.38
p53P:Plk1P	0.86	p53	3.2	Cdc20P	0.87	APC/CP	5.11
Plk1P	0.72	p53P:Plk1P	3.14	APC/CP	0.71	Cdc25P	4.78
APC/CP	0.71	p53T	2.96	Plk1P	0.7	ATM/ATR	4.57
Mad2:Cdc20P	0.7	p21:MPF	2.9	Mad2:Cdc20P	0.64	APC/CP:Cdc20	4.28
APC/CP:Cdh1	0.6	ATM/ATR	2.73	p21:MPF	0.58	Mad2:Cdc20P	4.19
Cdc25P	0.58	Mdm2	2.71	Cdc25P	0.56	p21:MPF	3.84
MPF (Cy-clinB:CDK1)	0.53	APC/CP:Cdh1	2.68	APC/CP:Cdh1	0.54	MPF (Cy-clinB:CDK1)	3.53
p21:MPF	0.5	APC/CP:Cdc20	1.52	MPF (Cy-clinB:CDK1)	0.53	LMNAP	3.07
Plk1	0.49	preMPF	1.49	Plk1	0.49	Plk1T	2.43
LMNAP	0.47	APC/CP	1.21	LMNAP	0.47	preMPF	1.63
Cdc20T	0.45	CyclinBT	1.06	Cdc20T	0.46	Wee1P	1.63
preMPF	0.44	p21T	1	preMPF	0.43	Cdc25	1.59
Pttg1P	0.44	p21	0.95	Plk1T	0.43	APC/CP:Cdh1	1.42
Pttg1T	0.43	APC/CPT	0.83	APC/C:Cdh1	0.43	PP2AP	1.39
Plk1T	0.42	Cdc25	0.81	Pttg1P	0.43	Mdm2	1.37
APC/C:Cdh1	0.42	Cdc20T	0.78	Pttg1T	0.42	CyclinB	1.33
Cdh1	0.39	Mad2:Cdc20P	0.78	Cdh1	0.39	Cdc20T	1.26
Cdc20	0.39	Pttg1P	0.74	Cdc20	0.39	Wee1	0.96

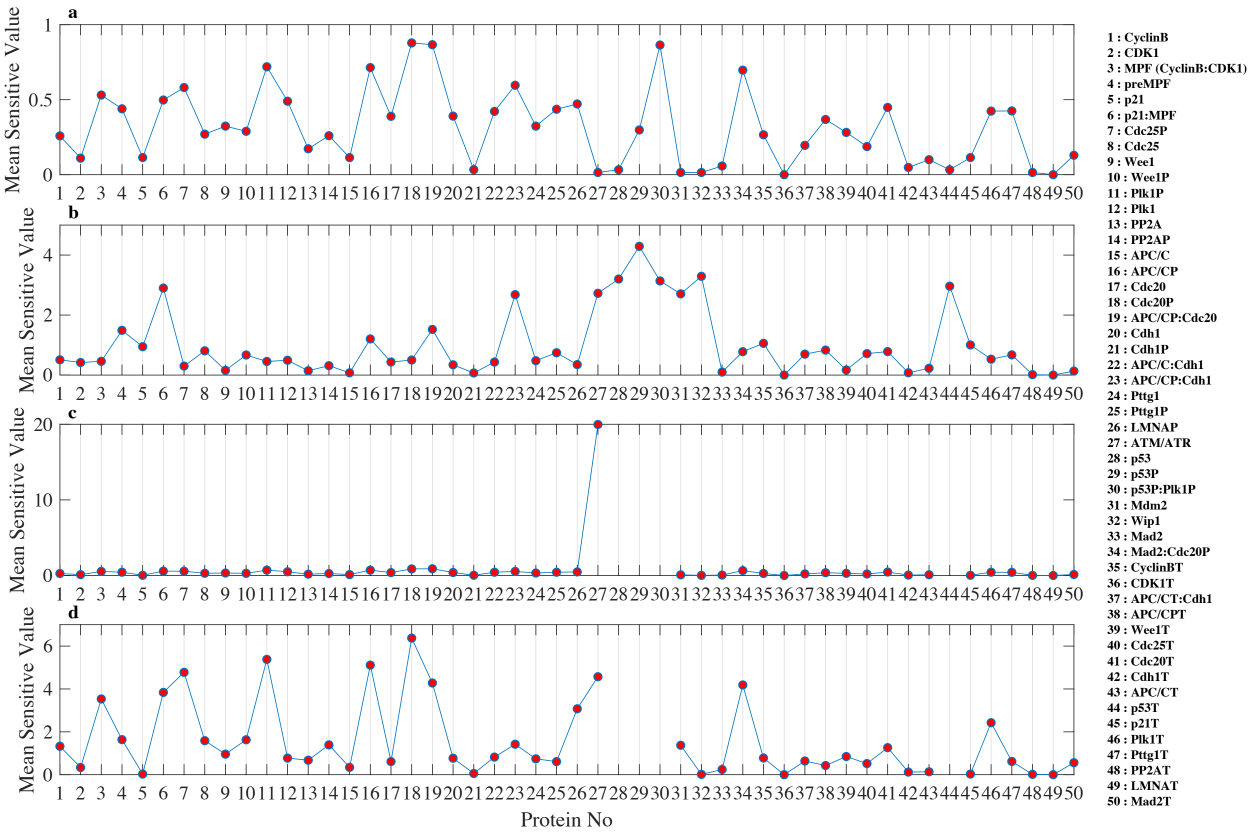

Supplementary Figure 3. The average logarithmic sensitivity intensities for 50 model components for WT (**a**), Plk1 depleted WT (**b**), p53-null cancer (**c**) and Plk1-depleted p53-null cancer (**d**) cells.

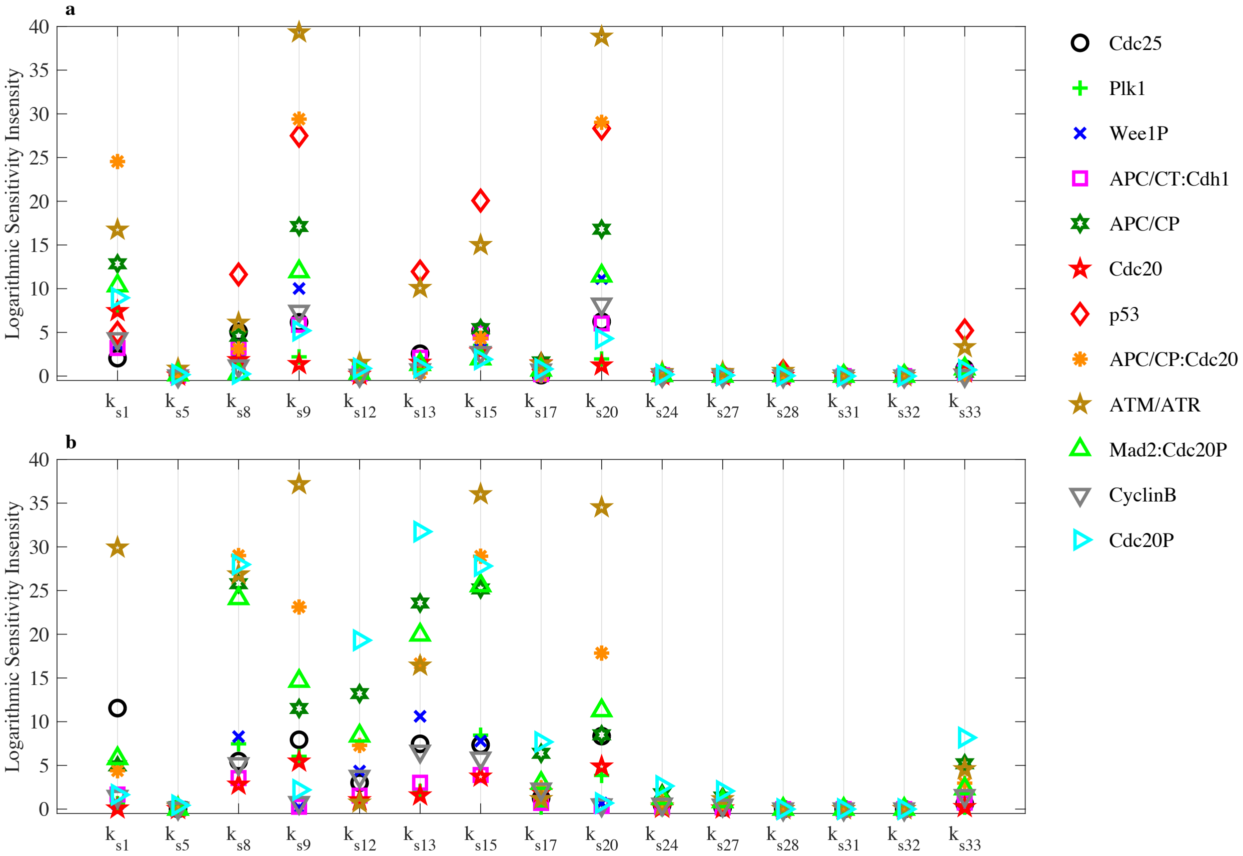

Supplementary Figure 4. Logarithmic intensities for Mad2, Wee1, Cdc20, Cdc25P, PP2AP, Plk1T, ATM/ATR, p53, Mdm2, Wip1, p21, Mad2:Cdc20P, Cdh1, MPF regulators in WT (a) and p53-null cancer (b) cells. The sensitivity intensities are obtained by varying k_{s1}, k_{s5}, k_{s8}, k_{s9}, k_{s12}, k_{s13}, k_{s15}, k_{s17}, k_{s20} , k_{s24}, k_{s27}, k_{s28}, k_{s31}, k_{s32}, k_{s33} parameters that control synthesis rates of Cyclin B, p21, cdc25, Wee1, Plk1, PP2A, APC/C, Cdc20, Cdh1, Pttg1, ATM/ATR, p53, Mdm2, Wip1, Mad2 correspondingly.

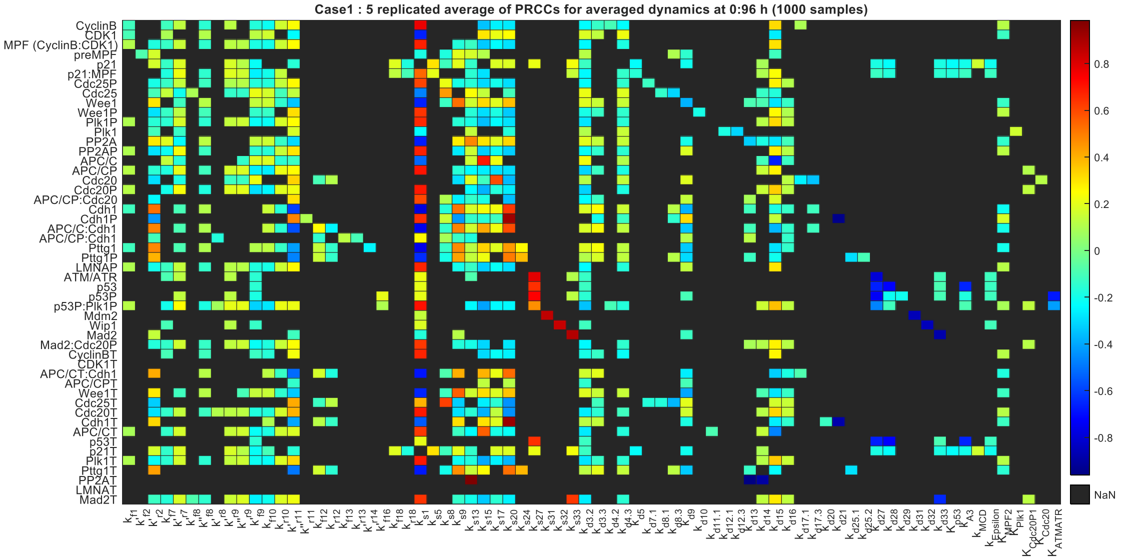
Case1 : 5 replicated average of PRCCs for averaged dynamics at 0:96 h (1000 samples)
CyclinB
CDK1
MPF (CyclinB:CDK1)
preMPF
p21
p21:MPF
Cdc25P
Cdc25
Wee1
Wee1P
Plk1P
Plk1
PP2A
PP2AP
APC/C
APC/CP
Cdc20
Cdc20P
APC/CP:Cdc20
Cdh1
Cdh1P
APC/C:Cdh1
APC/CP:Cdh1
Pttg1
Pttg1P
LMNAP
ATM/ATR
p53
p53P
p53P:Plk1P
Mdm2
Wip1
Mad2
Mad2:Cdc20P
CyclinBT
CDK1T
APC/CT:Cdh1
APC/CPT
Wee1T
Cdc25T
Cdc20T
Cdh1T
APC/CT
p53T
p21T
Plk1T
Pttg1T
PP2AT
LMNAT
Mad2T
0.8
0.6
0.4
0.2
0
-0.2
-0.4
-0.6
-0.8
NaN

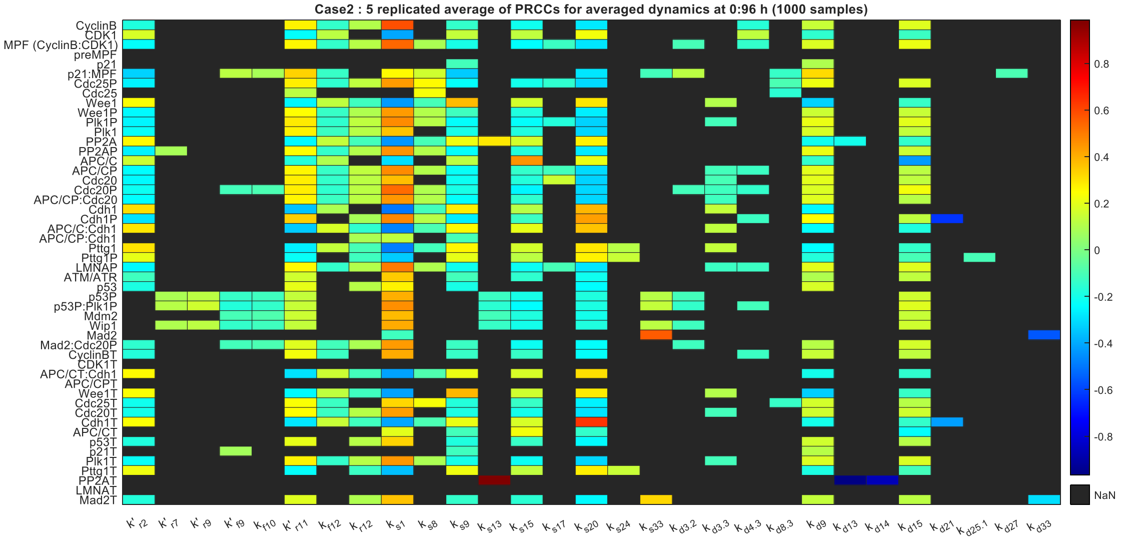
Case2 : 5 replicated average of PRCCs for averaged dynamics at 0:96 h (1000 samples)
CyclinB
CDK1
MPF (CyclinB:CDK1)
preMPF
p21
p21:MPF
Cdc25P
Cdc25
Wee1
Wee1P
Plk1P
Plk1
PP2A
PP2AP
APC/C
APC/CP
Cdc20
Cdc20P
APC/CP:Cdc20
Cdh1
Cdh1P
APC/C:Cdh1
APC/CP:Cdh1
Pttg1
Pttg1P
LMNAP
ATM/ATR
p53
p53P
p53P:Plk1P
Mdm2
Wip1
Mad2
Mad2:Cdc20P
CyclinBT
CDK1T
APC/CT:Cdh1
APC/CPT
Wee1T
Cdc25T
Cdc20T
Cdh1T
APC/CT
p53T
p21T
Plk1T
Pttg1T
PP2AT
LMNAT
Mad2T
0.8
0.6
0.4
0.2
0
-0.2
-0.4
-0.6
-0.8
NaN

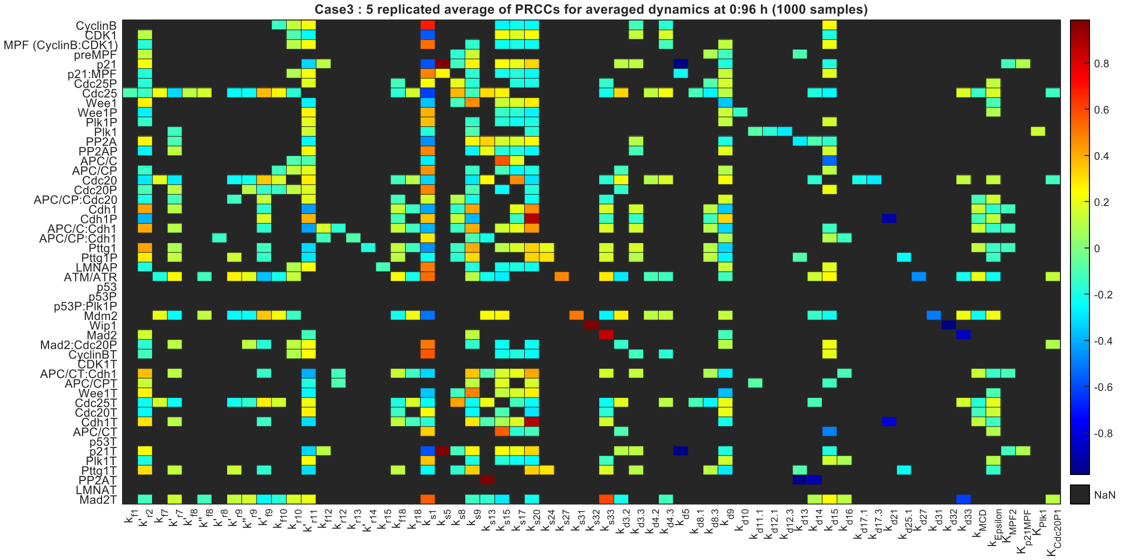

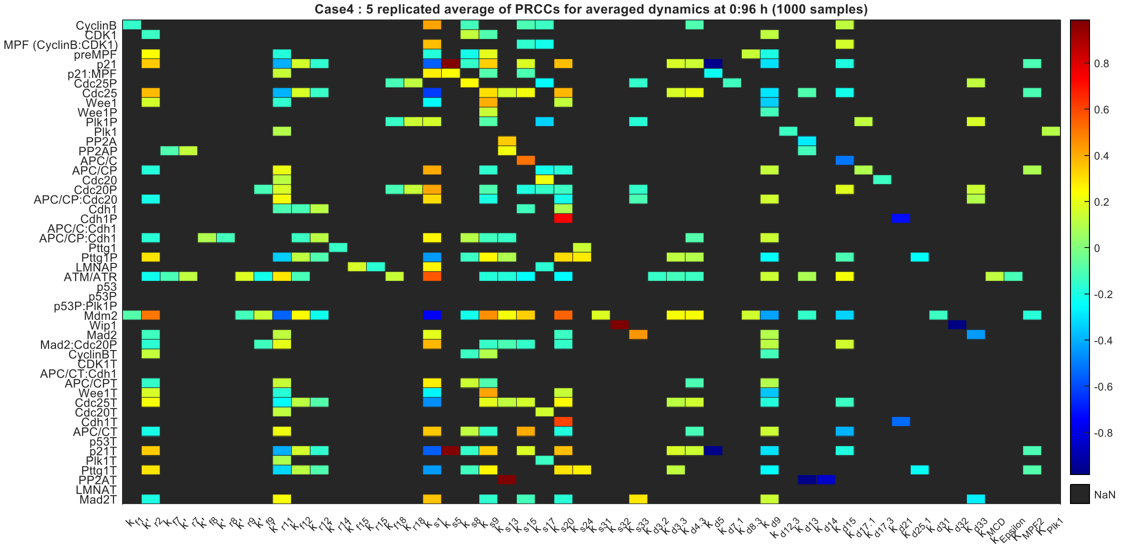

Supplementary Figure 5. Results of Partial Rank Correlation Coefficients (PRCC) analysis for the averaged dynamics of model components at time window 0:96h. Four heatmaps show mean PRCC values for the following four cases: 1) p53-wt Plk1-normal, 2) p53-wt Plk1 depleted by 45%, 3) p53-null Plk1-normal, 4) p53-null Plk1 depleted by 45%. Each pixel on the heatmap shows the statistically significant (p-value < 0.05) PRCC mean value for the corresponding protein (indicated along y-axis) and parameter (listed along x-axis). Black pixels ('NaN') show 'not a number' and represent no significant (p-value > 0.05) correlation between corresponding proteins and parameters. The mean of PRCC values are obtained using five replications of PRCC analysis (1000 runs).

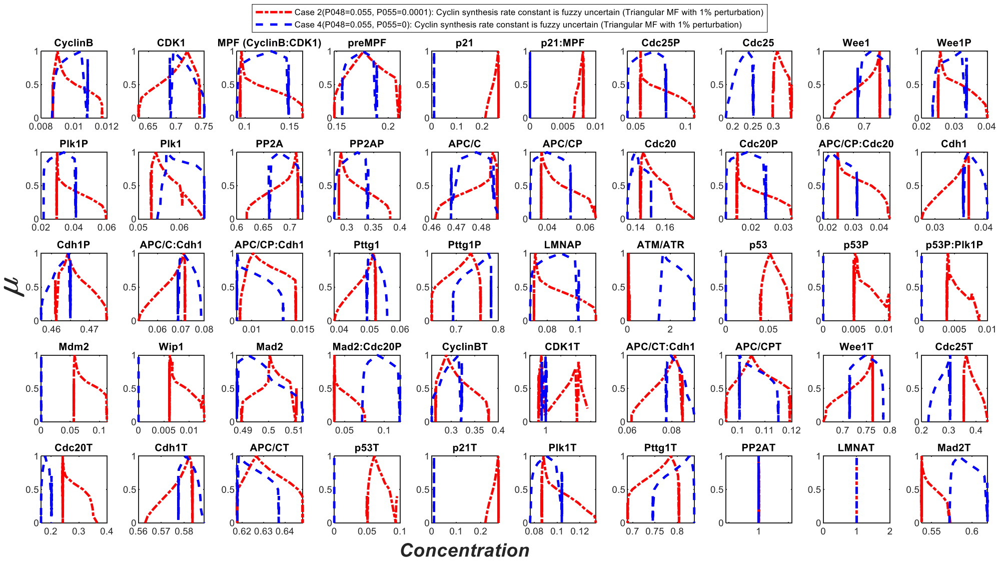
Case 2(P048=0.055, P055=0.0001): Cyclin synthesis rate constant is fuzzy uncertain (Triangular MF with 1% perturbation)
Case 4(P048=0.055, P055=0): Cyclin synthesis rate constant is fuzzy uncertain (Triangular MF with 1% perturbation)
CyclinB
CDK1
MPF (CyclinB:CDK1)
preMPF
p21
p21:MPF
Cdc25P
Cdc25
Wee1
Wee1P
Plk1P
Plk1
PP2A
PP2AP
APC/C
APC/CP
Cdc20
Cdc20P
APC/CP:Cdc20
Cdh1
Cdh1P
APC/C:Cdh1
APC/CP:Cdh1
Pttg1
Pttg1P
LMNAP
ATM/ATR
p53
p53P
p53P:Plk1P
Mdm2
Wip1
Mad2
Mad2:Cdc20P
CyclinBT
CDK1T
APC/CT:Cdh1
APC/CPT
Wee1T
Cdc25T
Cdc20T
Cdh1T
APC/CT
p53T
p21T
Plk1T
Pttg1T
PP2AT
LMNAT
Mad2T
μ
Concentration

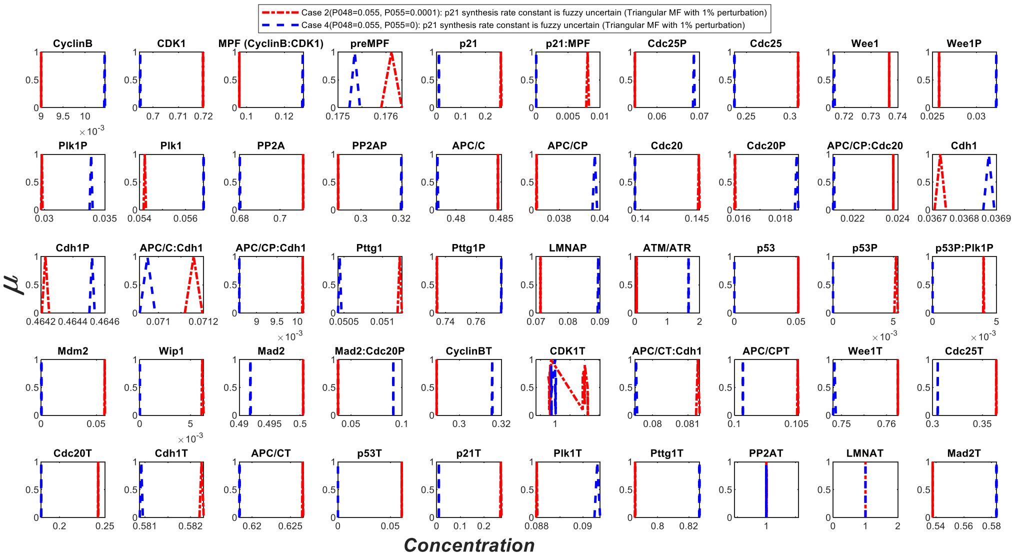
Case 2(P048=0.055, P055=0.0001): p21 synthesis rate constant is fuzzy uncertain (Triangular MF with 1% perturbation)
Case 4(P048=0.055, P055=0): p21 synthesis rate constant is fuzzy uncertain (Triangular MF with 1% perturbation)
CyclinB
CDK1
MPF (CyclinB:CDK1)
preMPF
p21
p21:MPF
Cdc25P
Cdc25
Wee1
Wee1P
Plk1P
Plk1
PP2A
PP2AP
APC/C
APC/CP
Cdc20
Cdc20P
APC/CP:Cdc20
Cdh1
Cdh1P
APC/C:Cdh1
APC/CP:Cdh1
Pttg1
Pttg1P
LMNAP
ATM/ATR
p53
p53P
p53P:Plk1P
Mdm2
Wip1
Mad2
Mad2:Cdc20P
CyclinBT
CDK1T
APC/CT:Cdh1
APC/CPT
Wee1T
Cdc25T
Cdc20T
Cdh1T
APC/CT
p53T
p21T
Plk1T
Pttg1T
PP2AT
LMNAT
Mad2T
μ
Concentration

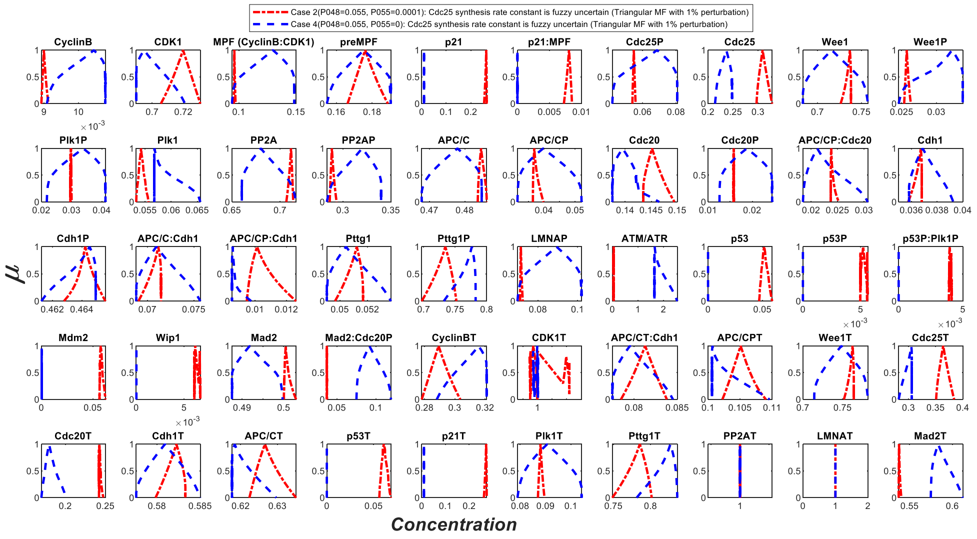
Case 2(P048=0.055, P055=0.0001): Cdc25 synthesis rate constant is fuzzy uncertain (Triangular MF with 1% perturbation)
Case 4(P048=0.055, P055=0): Cdc25 synthesis rate constant is fuzzy uncertain (Triangular MF with 1% perturbation)
CyclinB
CDK1
MPF (CyclinB:CDK1)
preMPF
p21
p21:MPF
Cdc25P
Cdc25
Wee1
Wee1P
Plk1P
Plk1
PP2A
PP2AP
APC/C
APC/CP
Cdc20
Cdc20P
APC/CP:Cdc20
Cdh1
Cdh1P
APC/C:Cdh1
APC/CP:Cdh1
Pttg1
Pttg1P
LMNAP
ATM/ATR
p53
p53P
p53P:Plk1P
Mdm2
Wip1
Mad2
Mad2:Cdc20P
CyclinBT
CDK1T
APC/CT:Cdh1
APC/CPT
Wee1T
Cdc25T
Cdc20T
Cdh1T
APC/CT
p53T
p21T
Plk1T
Pttg1T
PP2AT
LMNAT
Mad2T
μ
Concentration

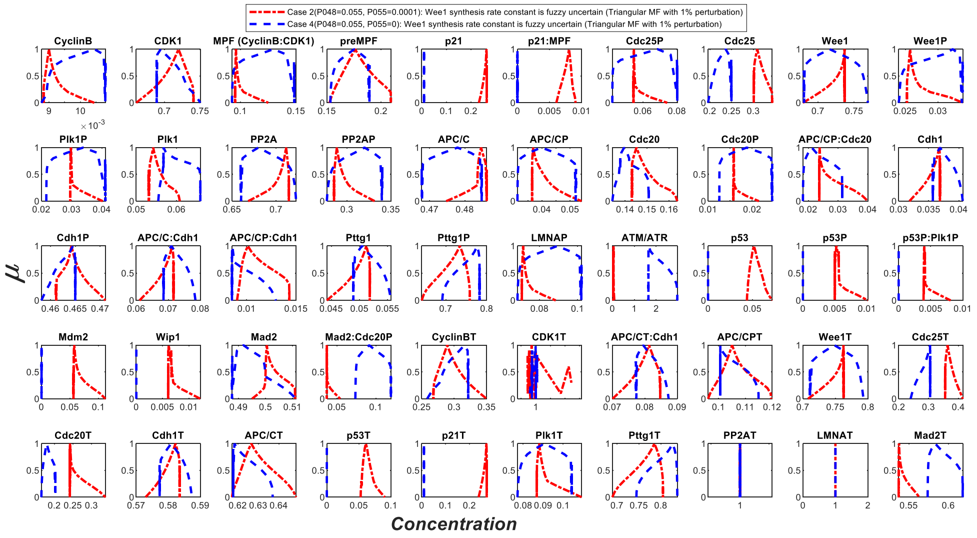
Case 2(P048=0.055, P055=0.0001): Wee1 synthesis rate constant is fuzzy uncertain (Triangular MF with 1% perturbation)
Case 4(P048=0.055, P055=0): Wee1 synthesis rate constant is fuzzy uncertain (Triangular MF with 1% perturbation)
CyclinB
CDK1
MPF (CyclinB:CDK1)
preMPF
p21
p21:MPF
Cdc25P
Cdc25
Wee1
Wee1P
Plk1P
Plk1
PP2A
PP2AP
APC/C
APC/CP
Cdc20
Cdc20P
APC/CP:Cdc20
Cdh1
Cdh1P
APC/C:Cdh1
APC/CP:Cdh1
Pttg1
Pttg1P
LMNAP
ATM/ATR
p53
p53P
p53P:Plk1P
Mdm2
Wip1
Mad2
Mad2:Cdc20P
CyclinBT
CDK1T
APC/CT:Cdh1
APC/CPT
Wee1T
Cdc25T
Cdc20T
Cdh1T
APC/CT
p53T
p21T
Plk1T
Pttg1T
PP2AT
LMNAT
Mad2T
μ
Concentration

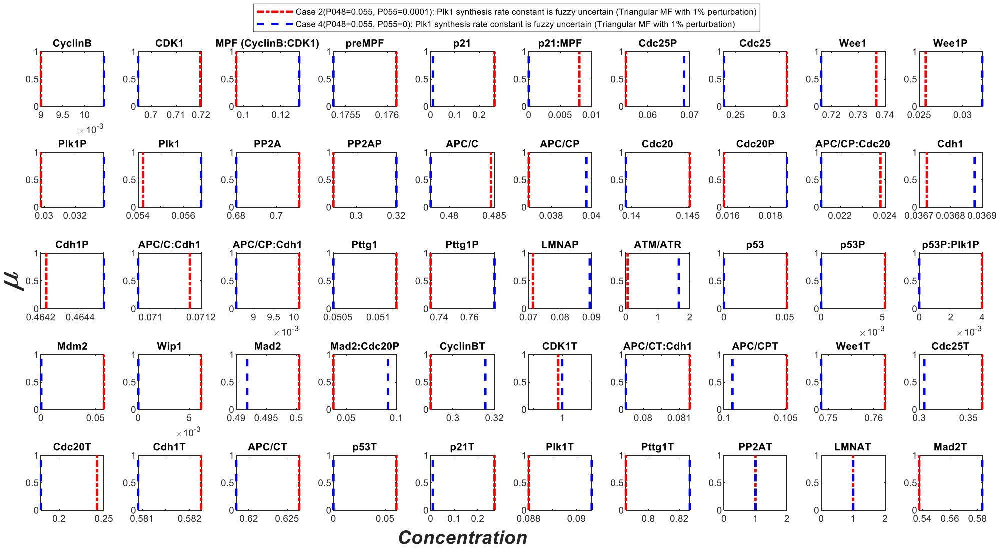

Case 2(P048=0.055, P055=0.0001): Plk1 synthesis rate constant is fuzzy uncertain (Triangular MF with 1% perturbation)
Case 4(P048=0.055, P055=0): Plk1 synthesis rate constant is fuzzy uncertain (Triangular MF with 1% perturbation)
CyclinB
CDK1
MPF (CyclinB:CDK1)
preMPF
p21
p21:MPF
Cdc25P
Cdc25
Wee1
Wee1P
Plk1P
Plk1
PP2A
PP2AP
APC/C
APC/CP
Cdc20
Cdc20P
APC/CP:Cdc20
Cdh1
Cdh1P
APC/C:Cdh1
APC/CP:Cdh1
Pttg1
Pttg1P
LMNAP
ATM/ATR
p53
p53P
p53P:Plk1P
Mdm2
Wip1
Mad2
Mad2:Cdc20P
CyclinBT
CDK1T
APC/CT:Cdh1
APC/CPT
Wee1T
Cdc25T
Cdc20T
Cdh1T
APC/CT
p53T
p21T
Plk1T
Pttg1T
PP2AT
LMNAT
Mad2T
μ
Concentration

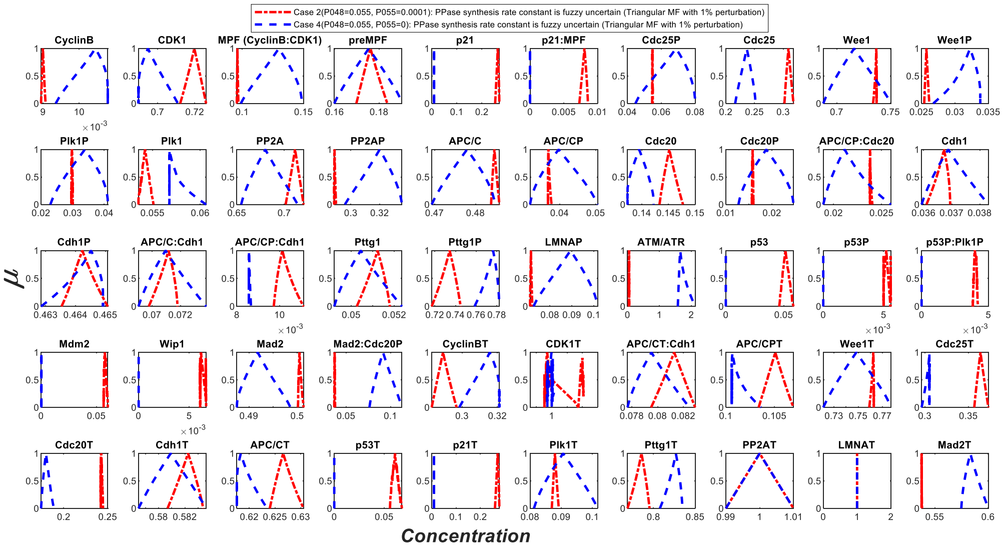

Case 2(P048=0.055, P055=0.0001): PPase synthesis rate constant is fuzzy uncertain (Triangular MF with 1% perturbation)
Case 4(P048=0.055, P055=0): PPase synthesis rate constant is fuzzy uncertain (Triangular MF with 1% perturbation)
CyclinB
CDK1
MPF (CyclinB:CDK1)
preMPF
p21
p21:MPF
Cdc25P
Cdc25
Wee1
Wee1P
Plk1P
Plk1
PP2A
PP2AP
APC/C
APC/CP
Cdc20
Cdc20P
APC/CP:Cdc20
Cdh1
Cdh1P
APC/C:Cdh1
APC/CP:Cdh1
Pttg1
Pttg1P
LMNAP
ATM/ATR
p53
p53P
p53P:Plk1P
Mdm2
Wip1
Mad2
Mad2:Cdc20P
CyclinBT
CDK1T
APC/CT:Cdh1
APC/CPT
Wee1T
Cdc25T
Cdc20T
Cdh1T
APC/CT
p53T
p21T
Plk1T
Pttg1T
PP2AT
LMNAT
Mad2T
μ
Concentration

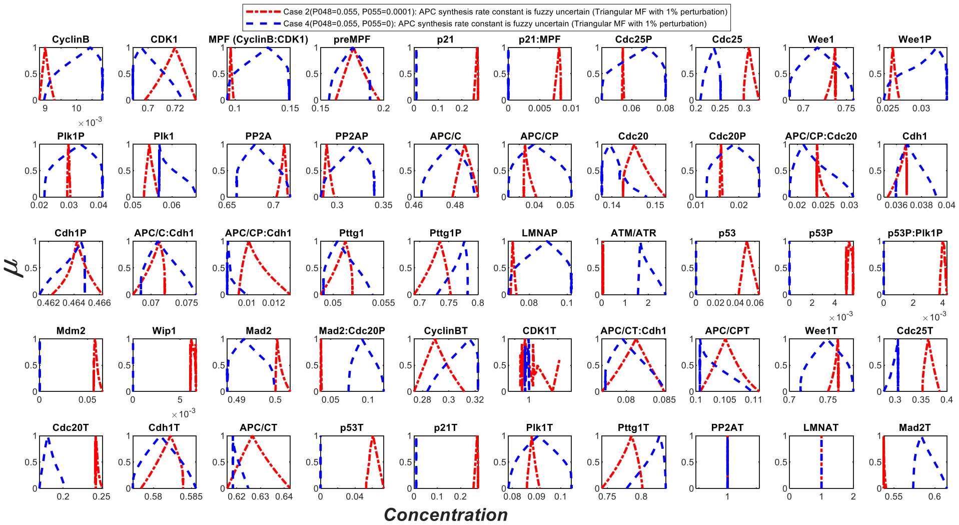
Case 2(P048=0.055, P055=0.0001): APC synthesis rate constant is fuzzy uncertain (Triangular MF with 1% perturbation)
Case 4(P048=0.055, P055=0): APC synthesis rate constant is fuzzy uncertain (Triangular MF with 1% perturbation)
CyclinB
CDK1
MPF (CyclinB:CDK1)
preMPF
p21
p21:MPF
Cdc25P
Cdc25
Wee1
Wee1P
Plk1P
Plk1
PP2A
PP2AP
APC/C
APC/CP
Cdc20
Cdc20P
APC/CP:Cdc20
Cdh1
Cdh1P
APC/C:Cdh1
APC/CP:Cdh1
Pttg1
Pttg1P
LMNAP
ATM/ATR
p53
p53P
p53P:Plk1P
Mdm2
Wip1
Mad2
Mad2:Cdc20P
CyclinBT
CDK1T
APC/CT:Cdh1
APC/CPT
Wee1T
Cdc25T
Cdc20T
Cdh1T
APC/CT
p53T
p21T
Plk1T
Pttg1T
PP2AT
LMNAT
Mad2T
μ
Concentration

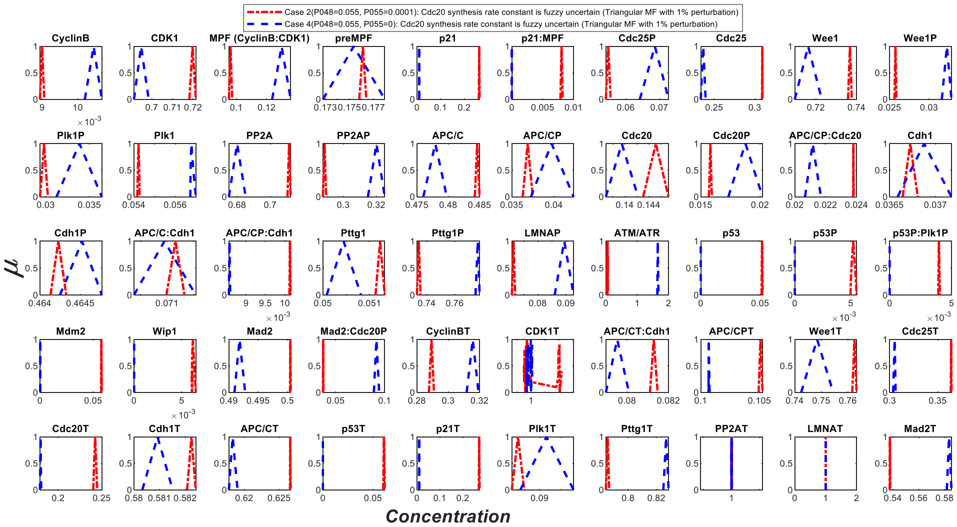
Case 2(P048=0.055, P055=0.0001): Cdc20 synthesis rate constant is fuzzy uncertain (Triangular MF with 1% perturbation)
Case 4(P048=0.055, P055=0): Cdc20 synthesis rate constant is fuzzy uncertain (Triangular MF with 1% perturbation)
CyclinB
CDK1
MPF (CyclinB:CDK1)
preMPF
p21
p21:MPF
Cdc25P
Cdc25
Wee1
Wee1P
Plk1P
Plk1
PP2A
PP2AP
APC/C
APC/CP
Cdc20
Cdc20P
APC/CP:Cdc20
Cdh1
Cdh1P
APC/C:Cdh1
APC/CP:Cdh1
Pttg1
Pttg1P
LMNAP
ATM/ATR
p53
p53P
p53P:Plk1P
Mdm2
Wip1
Mad2
Mad2:Cdc20P
CyclinBT
CDK1T
APC/CT:Cdh1
APC/CPT
Wee1T
Cdc25T
Cdc20T
Cdh1T
APC/CT
p53T
p21T
Plk1T
Pttg1T
PP2AT
LMNAT
Mad2T
μ
Concentration

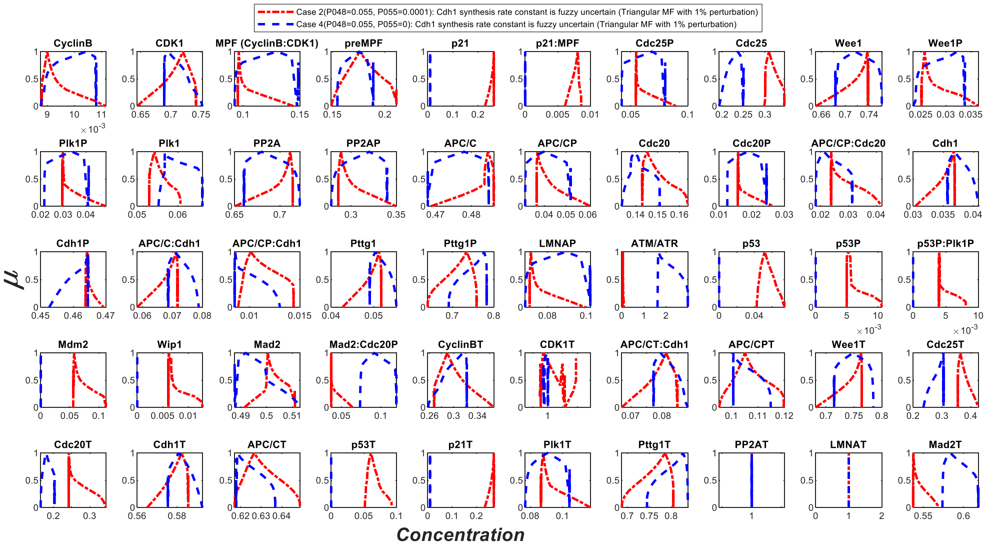
Case 2(P048=0.055, P055=0.0001): Cdh1 synthesis rate constant is fuzzy uncertain (Triangular MF with 1% perturbation)
Case 4(P048=0.055, P055=0): Cdh1 synthesis rate constant is fuzzy uncertain (Triangular MF with 1% perturbation)
CyclinB
CDK1
MPF (CyclinB:CDK1)
preMPF
p21
p21:MPF
Cdc25P
Cdc25
Wee1
Wee1P
Plk1P
Plk1
PP2A
PP2AP
APC/C
APC/CP
Cdc20
Cdc20P
APC/CP:Cdc20
Cdh1
Cdh1P
APC/C:Cdh1
APC/CP:Cdh1
Pttg1
Pttg1P
LMNAP
ATM/ATR
p53
p53P
p53P:Plk1P
Mdm2
Wip1
Mad2
Mad2:Cdc20P
CyclinBT
CDK1T
APC/CT:Cdh1
APC/CPT
Wee1T
Cdc25T
Cdc20T
Cdh1T
APC/CT
p53T
p21T
Plk1T
Pttg1T
PP2AT
LMNAT
Mad2T
μ
Concentration

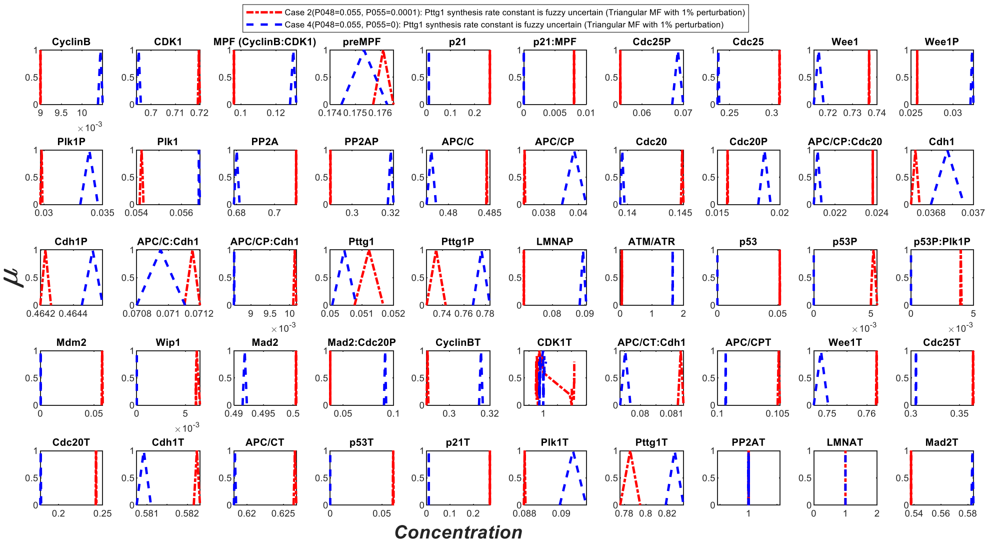
Case 2(P048=0.055, P055=0.0001): Pttg1 synthesis rate constant is fuzzy uncertain (Triangular MF with 1% perturbation)
Case 4(P048=0.055, P055=0): Pttg1 synthesis rate constant is fuzzy uncertain (Triangular MF with 1% perturbation)
CyclinB
CDK1
MPF (CyclinB:CDK1)
preMPF
p21
p21:MPF
Cdc25P
Cdc25
Wee1
Wee1P
Plk1P
Plk1
PP2A
PP2AP
APC/C
APC/CP
Cdc20
Cdc20P
APC/CP:Cdc20
Cdh1
Cdh1P
APC/C:Cdh1
APC/CP:Cdh1
Pttg1
Pttg1P
LMNAP
ATM/ATR
p53
p53P
p53P:Plk1P
Mdm2
Wip1
Mad2
Mad2:Cdc20P
CyclinBT
CDK1T
APC/CT:Cdh1
APC/CPT
Wee1T
Cdc25T
Cdc20T
Cdh1T
APC/CT
p53T
p21T
Plk1T
Pttg1T
PP2AT
LMNAT
Mad2T
μ
Concentration

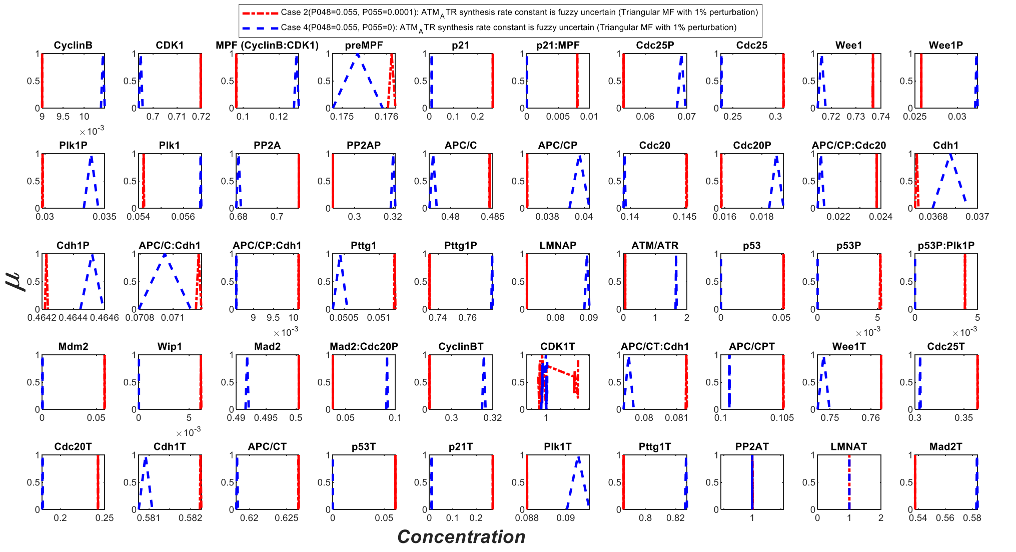

Case 2(P048=0.055, P055=0.0001): ATM$_A$TR synthesis rate constant is fuzzy uncertain (Triangular MF with 1% perturbation)
Case 4(P048=0.055, P055=0): ATM$_A$TR synthesis rate constant is fuzzy uncertain (Triangular MF with 1% perturbation)
CyclinB
CDK1
MPF (CyclinB:CDK1)
preMPF
p21
p21:MPF
Cdc25P
Cdc25
Wee1
Wee1P
Plk1P
Plk1
PP2A
PP2AP
APC/C
APC/CP
Cdc20
Cdc20P
APC/CP:Cdc20
Cdh1
Cdh1P
APC/C:Cdh1
APC/CP:Cdh1
Pttg1
Pttg1P
LMNAP
ATM/ATR
p53
p53P
p53P:Plk1P
Mdm2
Wip1
Mad2
Mad2:Cdc20P
CyclinBT
CDK1T
APC/CT:Cdh1
APC/CPT
Wee1T
Cdc25T
Cdc20T
Cdh1T
APC/CT
p53T
p21T
Plk1T
Pttg1T
PP2AT
LMNAT
Mad2T
μ
Concentration

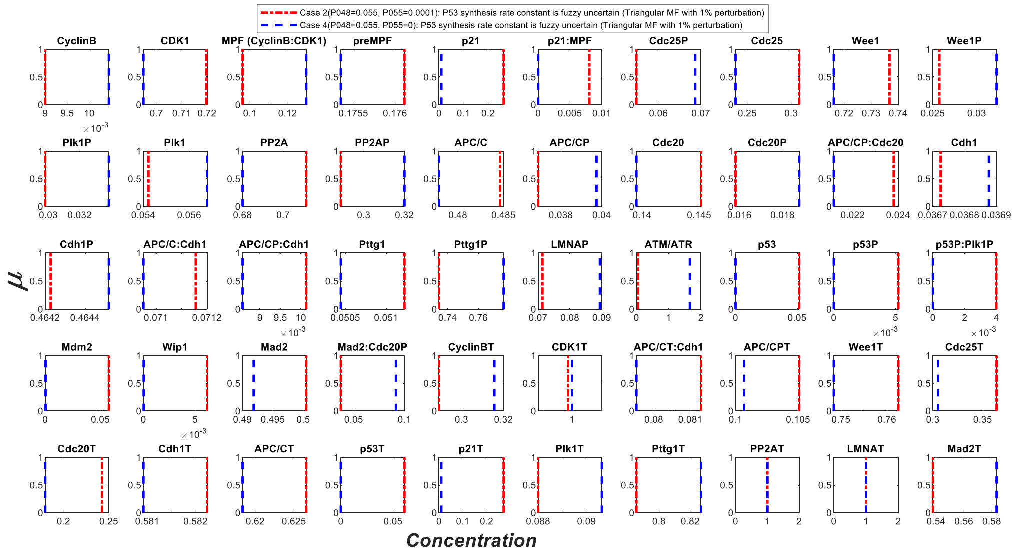

Case 2(P048=0.055, P055=0.0001): P53 synthesis rate constant is fuzzy uncertain (Triangular MF with 1% perturbation)
Case 4(P048=0.055, P055=0): P53 synthesis rate constant is fuzzy uncertain (Triangular MF with 1% perturbation)
CyclinB
CDK1
MPF (CyclinB:CDK1)
preMPF
p21
p21:MPF
Cdc25P
Cdc25
Wee1
Wee1P
Plk1P
Plk1
PP2A
PP2AP
APC/C
APC/CP
Cdc20
Cdc20P
APC/CP:Cdc20
Cdh1
Cdh1P
APC/C:Cdh1
APC/CP:Cdh1
Pttg1
Pttg1P
LMNAP
ATM/ATR
p53
p53P
p53P:Plk1P
Mdm2
Wip1
Mad2
Mad2:Cdc20P
CyclinBT
CDK1T
APC/CT:Cdh1
APC/CPT
Wee1T
Cdc25T
Cdc20T
Cdh1T
APC/CT
p53T
p21T
Plk1T
Pttg1T
PP2AT
LMNAT
Mad2T
μ
Concentration

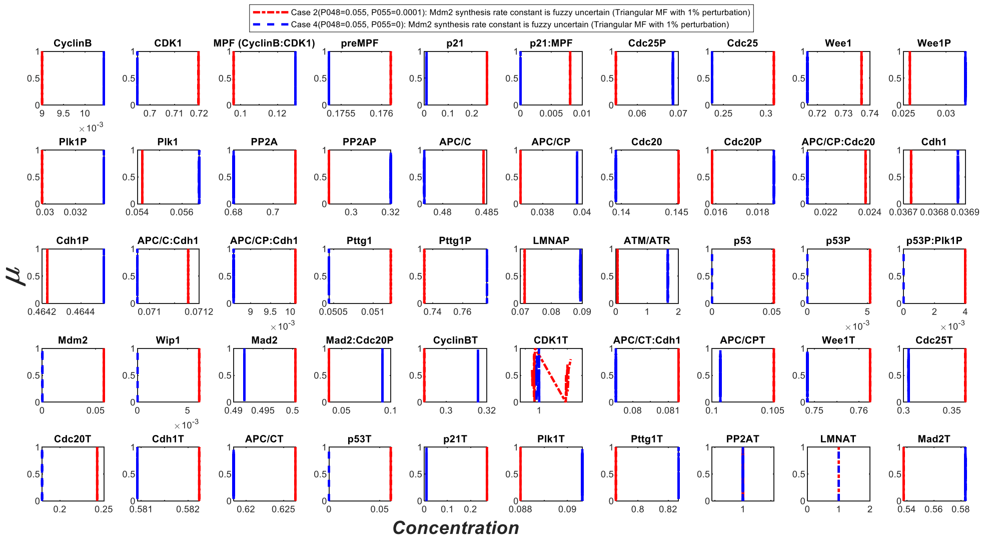
Case 2(P048=0.055, P055=0.0001): Mdm2 synthesis rate constant is fuzzy uncertain (Triangular MF with 1% perturbation)
Case 4(P048=0.055, P055=0): Mdm2 synthesis rate constant is fuzzy uncertain (Triangular MF with 1% perturbation)
CyclinB
CDK1
MPF (CyclinB:CDK1)
preMPF
p21
p21:MPF
Cdc25P
Cdc25
Wee1
Wee1P
Plk1P
Plk1
PP2A
PP2AP
APC/C
APC/CP
Cdc20
Cdc20P
APC/CP:Cdc20
Cdh1
Cdh1P
APC/C:Cdh1
APC/CP:Cdh1
Pttg1
Pttg1P
LMNAP
ATM/ATR
p53
p53P
p53P:Plk1P
Mdm2
Wip1
Mad2
Mad2:Cdc20P
CyclinBT
CDK1T
APC/CT:Cdh1
APC/CPT
Wee1T
Cdc25T
Cdc20T
Cdh1T
APC/CT
p53T
p21T
Plk1T
Pttg1T
PP2AT
LMNAT
Mad2T
μ
Concentration

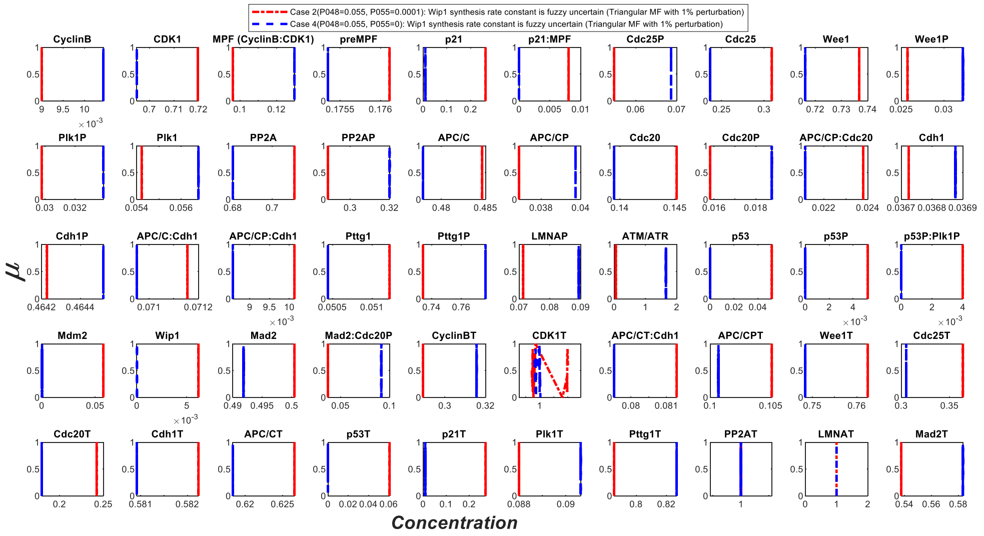
Case 2(P048=0.055, P055=0.0001): Wip1 synthesis rate constant is fuzzy uncertain (Triangular MF with 1% perturbation)
Case 4(P048=0.055, P055=0): Wip1 synthesis rate constant is fuzzy uncertain (Triangular MF with 1% perturbation)
CyclinB
CDK1
MPF (CyclinB:CDK1)
preMPF
p21
p21:MPF
Cdc25P
Cdc25
Wee1
Wee1P
Plk1P
Plk1
PP2A
PP2AP
APC/C
APC/CP
Cdc20
Cdc20P
APC/CP:Cdc20
Cdh1
Cdh1P
APC/C:Cdh1
APC/CP:Cdh1
Pttg1
Pttg1P
LMNAP
ATM/ATR
p53
p53P
p53P:Plk1P
Mdm2
Wip1
Mad2
Mad2:Cdc20P
CyclinBT
CDK1T
APC/CT:Cdh1
APC/CPT
Wee1T
Cdc25T
Cdc20T
Cdh1T
APC/CT
p53T
p21T
Plk1T
Pttg1T
PP2AT
LMNAT
Mad2T
μ
Concentration

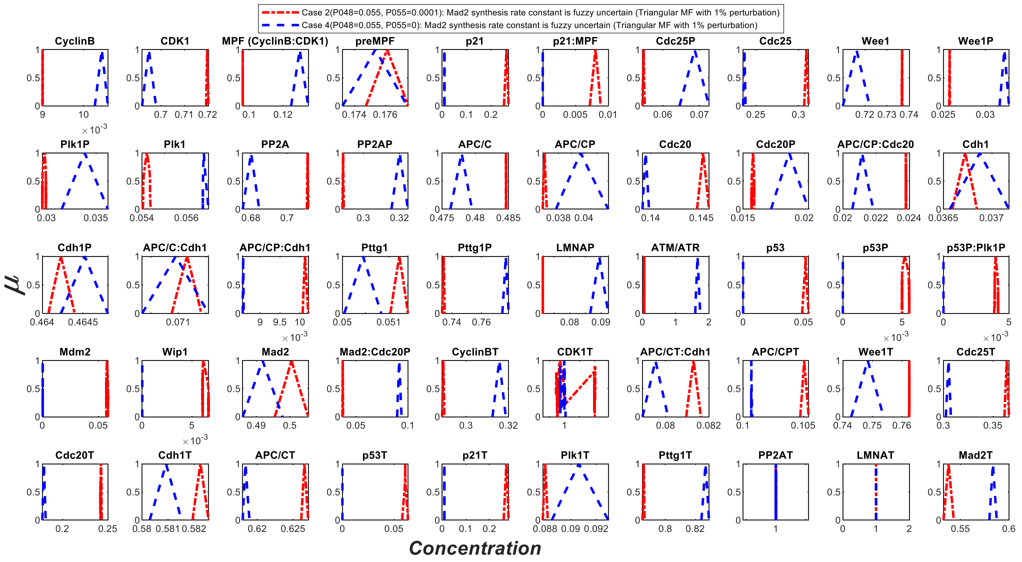

Figure 6. Fuzzy analysis for comparison of case 2 (red dot-dashed lines) (p53-wt Plk1 depleted by 45%) and case 4 (blue dashed lines) (p53-null Plk1 depleted by 45%). The analysis is performed for 50 model components (shown as titles for panels) by setting k_{s1}, k_{s5}, k_{s8}, k_{s9}, k_{s12}, k_{s13}, k_{s15}, k_{s17}, k_{s20}, k_{s24}, k_{s27}, k_{s28}, k_{s31} k_{s32}, k_{s33} as fuzzy parameters. These parameters control the synthesis of Cyclin B, p21, Cdc25, Wee1, Plk1, PP2A, APC/C, Cdc20, Cdh1, Pttg1, ATM/ATR, p53, Mdm2, Wip1, Mad2 proteins, respectively. Each parameter is perturbed by 1% of its value. The horizontal axis of each panel depicts the maximum uncertainty band of concentration of model components affected by uncertainty of the parameter. The vertical axis shows the different α-cut levels (by increasing the α value from zero, the uncertainty decreases and α = 1, depicts the crisp setting (no uncertainty)).

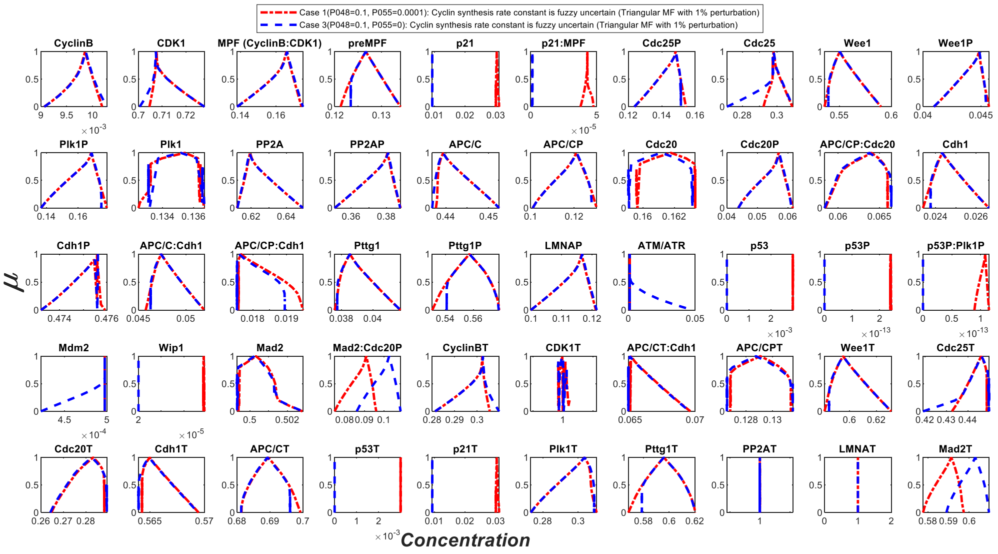
Case 1(P048=0.1, P055=0.0001): Cyclin synthesis rate constant is fuzzy uncertain (Triangular MF with 1% perturbation)
Case 3(P048=0.1, P055=0): Cyclin synthesis rate constant is fuzzy uncertain (Triangular MF with 1% perturbation)
CyclinB
CDK1
MPF (CyclinB:CDK1)
preMPF
p21
p21:MPF
Cdc25P
Cdc25
Wee1
Wee1P
Plk1P
Plk1
PP2A
PP2AP
APC/C
APC/CP
Cdc20
Cdc20P
APC/CP:Cdc20
Cdh1
Cdh1P
APC/C:Cdh1
APC/CP:Cdh1
Pttg1
Pttg1P
LMNAP
ATM/ATR
p53
p53P
p53P:Plk1P
Mdm2
Wip1
Mad2
Mad2:Cdc20P
CyclinBT
CDK1T
APC/CT:Cdh1
APC/CPT
Wee1T
Cdc25T
Cdc20T
Cdh1T
APC/CT
p53T
p21T
Plk1T
Pttg1T
PP2AT
LMNAT
Mad2T
μ
Concentration

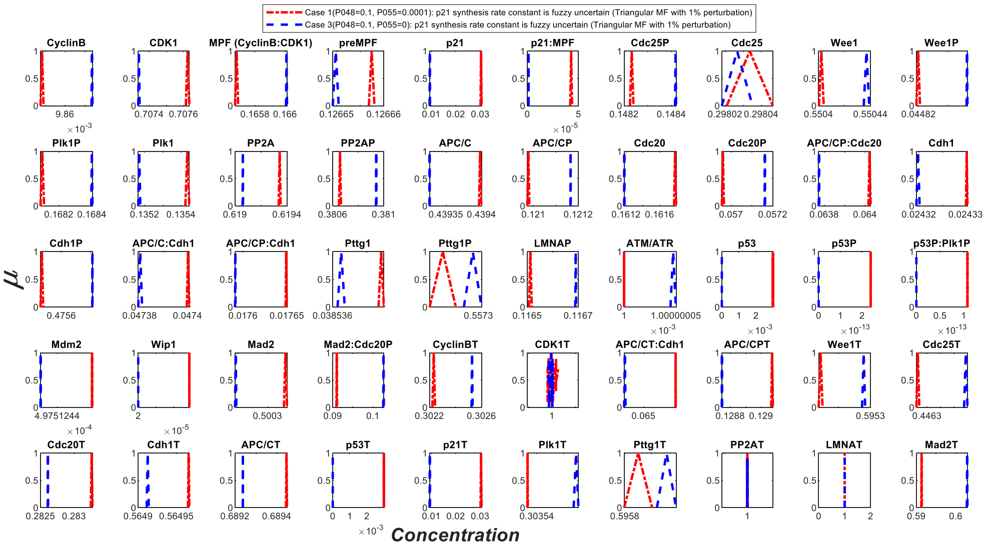
Case 1(P048=0.1, P055=0.0001): p21 synthesis rate constant is fuzzy uncertain (Triangular MF with 1% perturbation)
Case 3(P048=0.1, P055=0): p21 synthesis rate constant is fuzzy uncertain (Triangular MF with 1% perturbation)
CyclinB
CDK1
MPF (CyclinB:CDK1)
preMPF
p21
p21:MPF
Cdc25P
Cdc25
Wee1
Wee1P
Plk1P
Plk1
PP2A
PP2AP
APC/C
APC/CP
Cdc20
Cdc20P
APC/CP:Cdc20
Cdh1
Cdh1P
APC/C:Cdh1
APC/CP:Cdh1
Pttg1
Pttg1P
LMNAP
ATM/ATR
p53
p53P
p53P:Plk1P
Mdm2
Wip1
Mad2
Mad2:Cdc20P
CyclinBT
CDK1T
APC/CT:Cdh1
APC/CPT
Wee1T
Cdc25T
Cdc20T
Cdh1T
APC/CT
p53T
p21T
Plk1T
Pttg1T
PP2AT
LMNAT
Mad2T
μ
Concentration

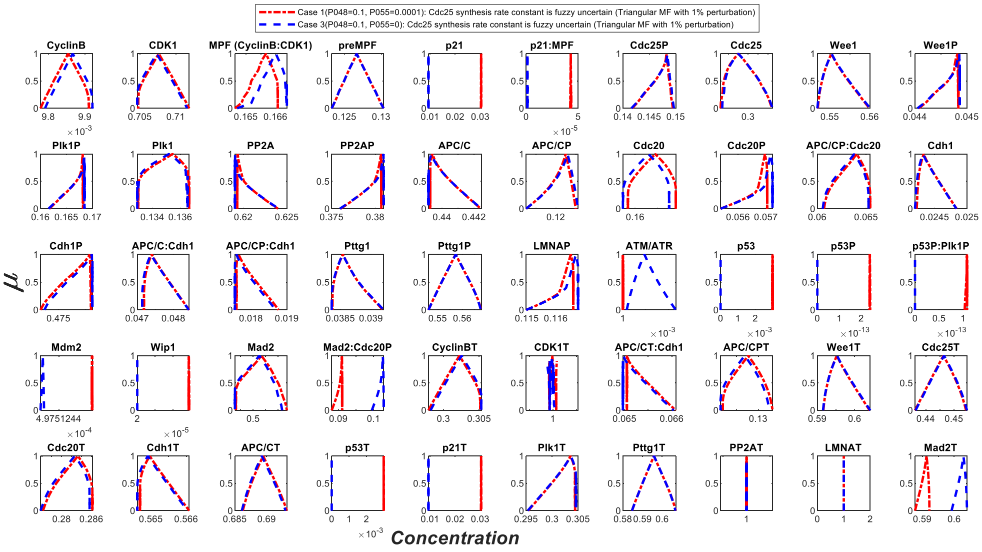
Case 1(P048=0.1, P055=0.0001): Cdc25 synthesis rate constant is fuzzy uncertain (Triangular MF with 1% perturbation)
Case 3(P048=0.1, P055=0): Cdc25 synthesis rate constant is fuzzy uncertain (Triangular MF with 1% perturbation)
CyclinB
CDK1
MPF (CyclinB:CDK1)
preMPF
p21
p21:MPF
Cdc25P
Cdc25
Wee1
Wee1P
Plk1P
Plk1
PP2A
PP2AP
APC/C
APC/CP
Cdc20
Cdc20P
APC/CP:Cdc20
Cdh1
Cdh1P
APC/C:Cdh1
APC/CP:Cdh1
Pttg1
Pttg1P
LMNAP
ATM/ATR
p53
p53P
p53P:Plk1P
Mdm2
Wip1
Mad2
Mad2:Cdc20P
CyclinBT
CDK1T
APC/CT:Cdh1
APC/CPT
Wee1T
Cdc25T
Cdc20T
Cdh1T
APC/CT
p53T
p21T
Plk1T
Pttg1T
PP2AT
LMNAT
Mad2T
μ
Concentration

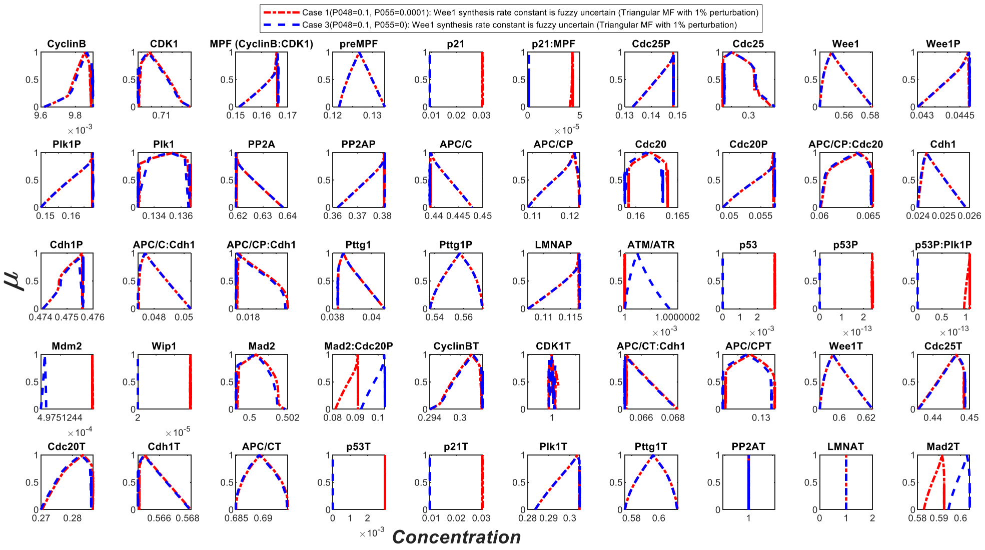
Case 1(P048=0.1, P055=0.0001): Wee1 synthesis rate constant is fuzzy uncertain (Triangular MF with 1% perturbation)
Case 3(P048=0.1, P055=0): Wee1 synthesis rate constant is fuzzy uncertain (Triangular MF with 1% perturbation)
CyclinB
CDK1
MPF (CyclinB:CDK1)
preMPF
p21
p21:MPF
Cdc25P
Cdc25
Wee1
Wee1P
Plk1P
Plk1
PP2A
PP2AP
APC/C
APC/CP
Cdc20
Cdc20P
APC/CP:Cdc20
Cdh1
Cdh1P
APC/C:Cdh1
APC/CP:Cdh1
Pttg1
Pttg1P
LMNAP
ATM/ATR
p53
p53P
p53P:Plk1P
Mdm2
Wip1
Mad2
Mad2:Cdc20P
CyclinBT
CDK1T
APC/CT:Cdh1
APC/CPT
Wee1T
Cdc25T
Cdc20T
Cdh1T
APC/CT
p53T
p21T
Plk1T
Pttg1T
PP2AT
LMNAT
Mad2T
μ
Concentration

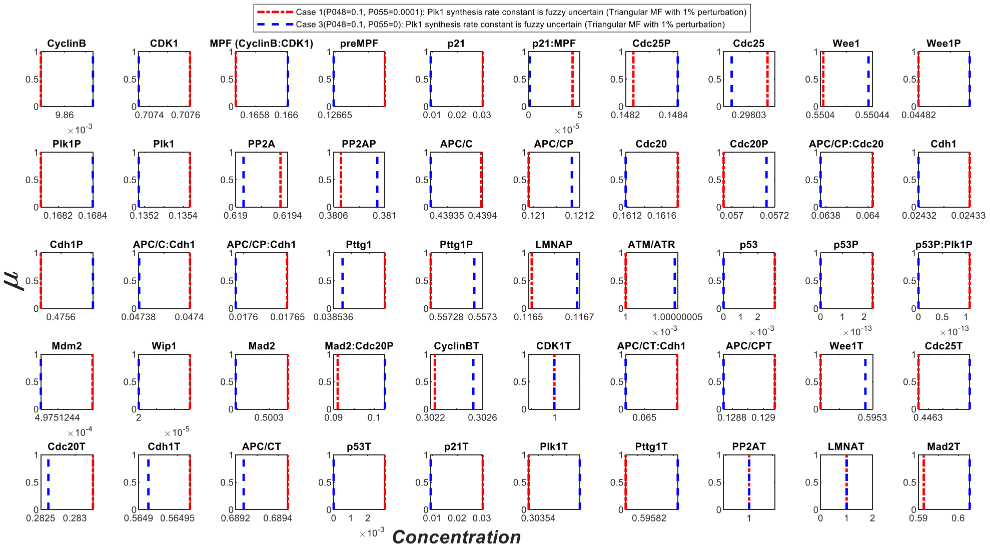

Case 1(P048=0.1, P055=0.0001): Plk1 synthesis rate constant is fuzzy uncertain (Triangular MF with 1% perturbation)
Case 3(P048=0.1, P055=0): Plk1 synthesis rate constant is fuzzy uncertain (Triangular MF with 1% perturbation)
CyclinB
CDK1
MPF (CyclinB:CDK1)
preMPF
p21
p21:MPF
Cdc25P
Cdc25
Wee1
Wee1P
Plk1P
Plk1
PP2A
PP2AP
APC/C
APC/CP
Cdc20
Cdc20P
APC/CP:Cdc20
Cdh1
Cdh1P
APC/C:Cdh1
APC/CP:Cdh1
Pttg1
Pttg1P
LMNAP
ATM/ATR
p53
p53P
p53P:Plk1P
Mdm2
Wip1
Mad2
Mad2:Cdc20P
CyclinBT
CDK1T
APC/CT:Cdh1
APC/CPT
Wee1T
Cdc25T
Cdc20T
Cdh1T
APC/CT
p53T
p21T
Plk1T
Pttg1T
PP2AT
LMNAT
Mad2T
μ
Concentration

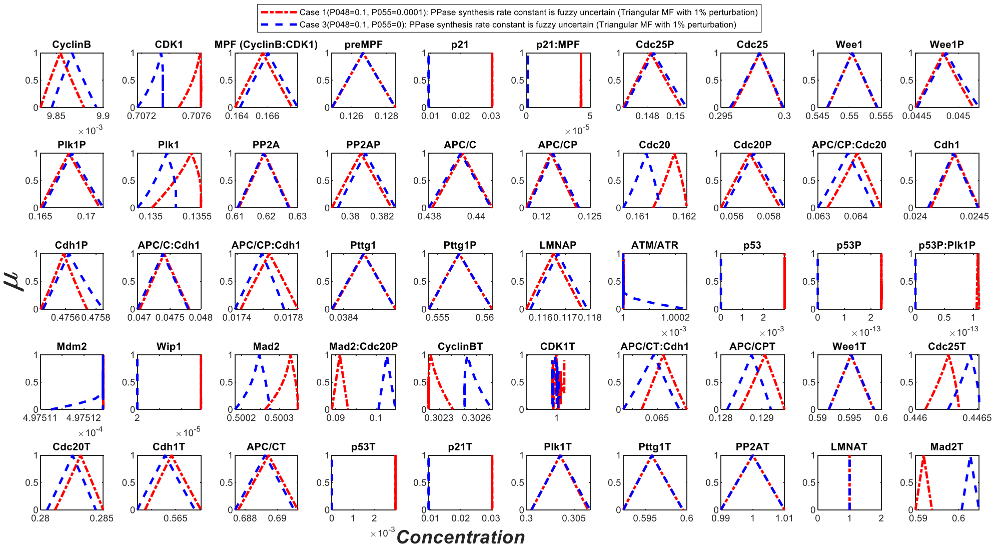

Case 1(P048=0.1, P055=0.0001): PPase synthesis rate constant is fuzzy uncertain (Triangular MF with 1% perturbation)
Case 3(P048=0.1, P055=0): PPase synthesis rate constant is fuzzy uncertain (Triangular MF with 1% perturbation)
CyclinB
CDK1
MPF (CyclinB:CDK1)
preMPF
p21
p21:MPF
Cdc25P
Cdc25
Wee1
Wee1P
Plk1P
Plk1
PP2A
PP2AP
APC/C
APC/CP
Cdc20
Cdc20P
APC/CP:Cdc20
Cdh1
Cdh1P
APC/C:Cdh1
APC/CP:Cdh1
Pttg1
Pttg1P
LMNAP
ATM/ATR
p53
p53P
p53P:Plk1P
Mdm2
Wip1
Mad2
Mad2:Cdc20P
CyclinBT
CDK1T
APC/CT:Cdh1
APC/CPT
Wee1T
Cdc25T
Cdc20T
Cdh1T
APC/CT
p53T
p21T
Plk1T
Pttg1T
PP2AT
LMNAT
Mad2T
μ
Concentration

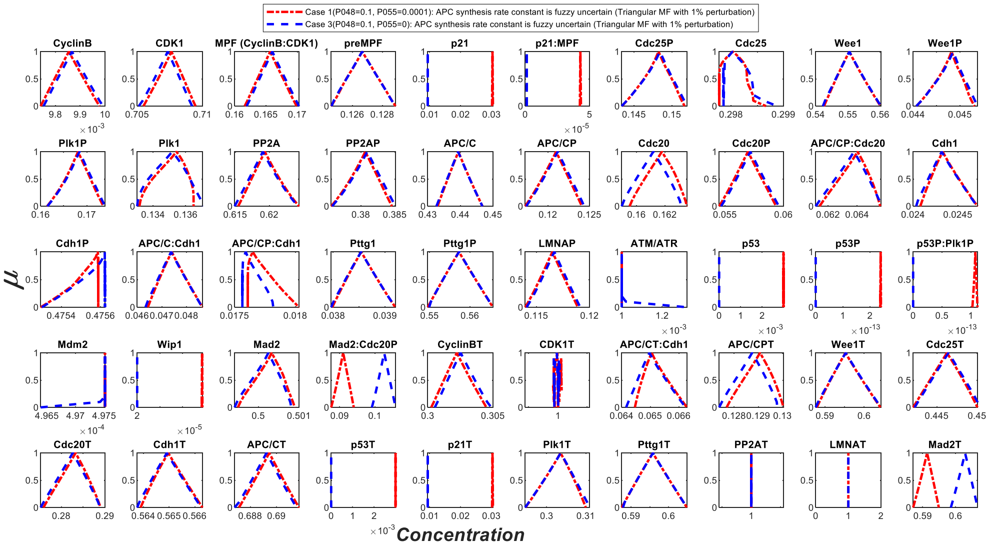
Case 1(P048=0.1, P055=0.0001): APC synthesis rate constant is fuzzy uncertain (Triangular MF with 1% perturbation)
Case 3(P048=0.1, P055=0): APC synthesis rate constant is fuzzy uncertain (Triangular MF with 1% perturbation)
CyclinB
CDK1
MPF (CyclinB:CDK1)
preMPF
p21
p21:MPF
Cdc25P
Cdc25
Wee1
Wee1P
Plk1P
Plk1
PP2A
PP2AP
APC/C
APC/CP
Cdc20
Cdc20P
APC/CP:Cdc20
Cdh1
Cdh1P
APC/C:Cdh1
APC/CP:Cdh1
Pttg1
Pttg1P
LMNAP
ATM/ATR
p53
p53P
p53P:Plk1P
Mdm2
Wip1
Mad2
Mad2:Cdc20P
CyclinBT
CDK1T
APC/CT:Cdh1
APC/CPT
Wee1T
Cdc25T
Cdc20T
Cdh1T
APC/CT
p53T
p21T
Plk1T
Pttg1T
PP2AT
LMNAT
Mad2T
μ
Concentration

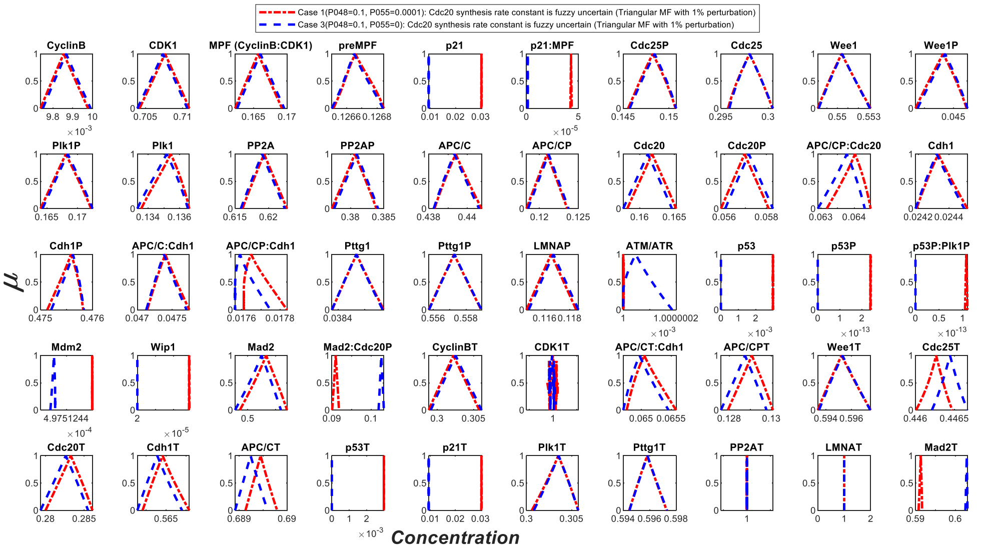
Case 1(P048=0.1, P055=0.0001): Cdc20 synthesis rate constant is fuzzy uncertain (Triangular MF with 1% perturbation)
Case 3(P048=0.1, P055=0): Cdc20 synthesis rate constant is fuzzy uncertain (Triangular MF with 1% perturbation)
CyclinB
CDK1
MPF (CyclinB:CDK1)
preMPF
p21
p21:MPF
Cdc25P
Cdc25
Wee1
Wee1P
Plk1P
Plk1
PP2A
PP2AP
APC/C
APC/CP
Cdc20
Cdc20P
APC/CP:Cdc20
Cdh1
Cdh1P
APC/C:Cdh1
APC/CP:Cdh1
Pttg1
Pttg1P
LMNAP
ATM/ATR
p53
p53P
p53P:Plk1P
Mdm2
Wip1
Mad2
Mad2:Cdc20P
CyclinBT
CDK1T
APC/CT:Cdh1
APC/CPT
Wee1T
Cdc25T
Cdc20T
Cdh1T
APC/CT
p53T
p21T
Plk1T
Pttg1T
PP2AT
LMNAT
Mad2T
μ
Concentration

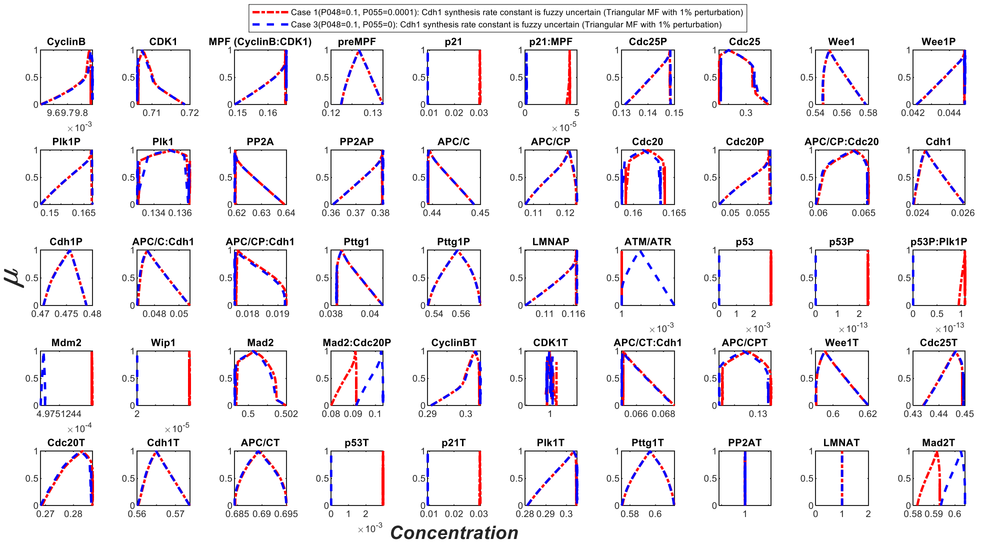
Case 1(P048=0.1, P055=0.0001): Cdh1 synthesis rate constant is fuzzy uncertain (Triangular MF with 1% perturbation)
Case 3(P048=0.1, P055=0): Cdh1 synthesis rate constant is fuzzy uncertain (Triangular MF with 1% perturbation)
CyclinB
CDK1
MPF (CyclinB:CDK1)
preMPF
p21
p21:MPF
Cdc25P
Cdc25
Wee1
Wee1P
Plk1P
Plk1
PP2A
PP2AP
APC/C
APC/CP
Cdc20
Cdc20P
APC/CP:Cdc20
Cdh1
Cdh1P
APC/C:Cdh1
APC/CP:Cdh1
Pttg1
Pttg1P
LMNAP
ATM/ATR
p53
p53P
p53P:Plk1P
Mdm2
Wip1
Mad2
Mad2:Cdc20P
CyclinBT
CDK1T
APC/CT:Cdh1
APC/CPT
Wee1T
Cdc25T
Cdc20T
Cdh1T
APC/CT
p53T
p21T
Plk1T
Pttg1T
PP2AT
LMNAT
Mad2T
μ
Concentration

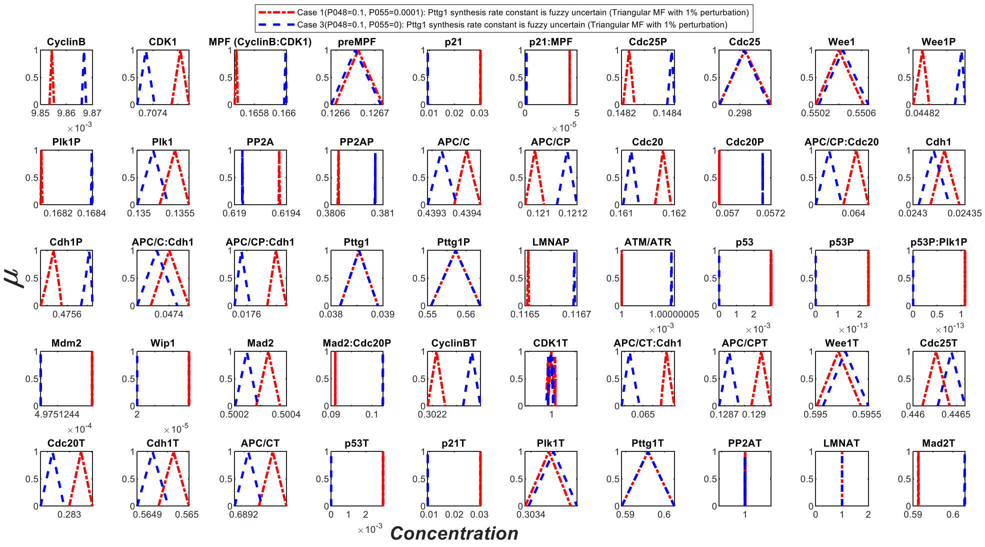
Case 1(P048=0.1, P055=0.0001): Pttg1 synthesis rate constant is fuzzy uncertain (Triangular MF with 1% perturbation)
Case 3(P048=0.1, P055=0): Pttg1 synthesis rate constant is fuzzy uncertain (Triangular MF with 1% perturbation)
CyclinB
CDK1
MPF (CyclinB:CDK1)
preMPF
p21
p21:MPF
Cdc25P
Cdc25
Wee1
Wee1P
Plk1P
Plk1
PP2A
PP2AP
APC/C
APC/CP
Cdc20
Cdc20P
APC/CP:Cdc20
Cdh1
Cdh1P
APC/C:Cdh1
APC/CP:Cdh1
Pttg1
Pttg1P
LMNAP
ATM/ATR
p53
p53P
p53P:Plk1P
Mdm2
Wip1
Mad2
Mad2:Cdc20P
CyclinBT
CDK1T
APC/CT:Cdh1
APC/CPT
Wee1T
Cdc25T
Cdc20T
Cdh1T
APC/CT
p53T
p21T
Plk1T
Pttg1T
PP2AT
LMNAT
Mad2T
μ
Concentration

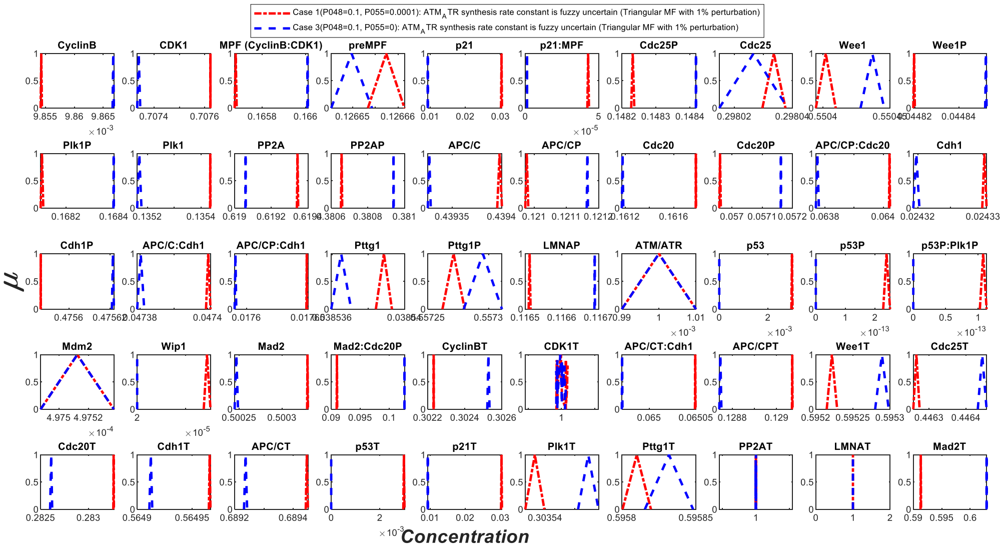
Case 1(P048=0.1, P055=0.0001): ATM$_A$TR synthesis rate constant is fuzzy uncertain (Triangular MF with 1% perturbation)
Case 3(P048=0.1, P055=0): ATM$_A$TR synthesis rate constant is fuzzy uncertain (Triangular MF with 1% perturbation)
CyclinB
CDK1
MPF (CyclinB:CDK1)
preMPF
p21
p21:MPF
Cdc25P
Cdc25
Wee1
Wee1P
Plk1P
Plk1
PP2A
PP2AP
APC/C
APC/CP
Cdc20
Cdc20P
APC/CP:Cdc20
Cdh1
Cdh1P
APC/C:Cdh1
APC/CP:Cdh1
Pttg1
Pttg1P
LMNAP
ATM/ATR
p53
p53P
p53P:Plk1P
Mdm2
Wip1
Mad2
Mad2:Cdc20P
CyclinBT
CDK1T
APC/CT:Cdh1
APC/CPT
Wee1T
Cdc25T
Cdc20T
Cdh1T
APC/CT
p53T
p21T
Plk1T
Pttg1T
PP2AT
LMNAT
Mad2T
μ
Concentration

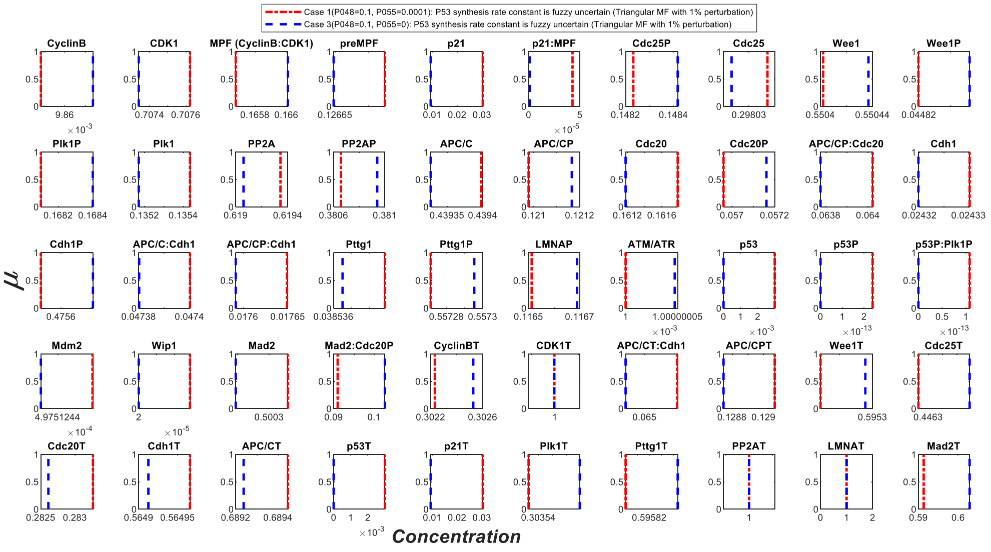
Case 1(P048=0.1, P055=0.0001): P53 synthesis rate constant is fuzzy uncertain (Triangular MF with 1% perturbation)
Case 3(P048=0.1, P055=0): P53 synthesis rate constant is fuzzy uncertain (Triangular MF with 1% perturbation)
CyclinB
CDK1
MPF (CyclinB:CDK1)
preMPF
p21
p21:MPF
Cdc25P
Cdc25
Wee1
Wee1P
Plk1P
Plk1
PP2A
PP2AP
APC/C
APC/CP
Cdc20
Cdc20P
APC/CP:Cdc20
Cdh1
Cdh1P
APC/C:Cdh1
APC/CP:Cdh1
Pttg1
Pttg1P
LMNAP
ATM/ATR
p53
p53P
p53P:Plk1P
Mdm2
Wip1
Mad2
Mad2:Cdc20P
CyclinBT
CDK1T
APC/CT:Cdh1
APC/CPT
Wee1T
Cdc25T
Cdc20T
Cdh1T
APC/CT
p53T
p21T
Plk1T
Pttg1T
PP2AT
LMNAT
Mad2T
μ
Concentration

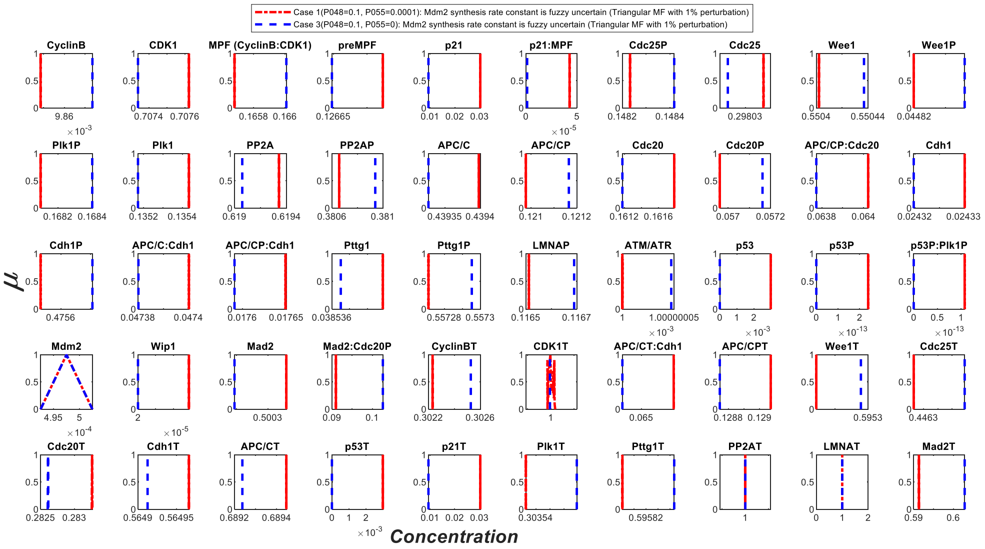
Case 1(P048=0.1, P055=0.0001): Mdm2 synthesis rate constant is fuzzy uncertain (Triangular MF with 1% perturbation)
Case 3(P048=0.1, P055=0): Mdm2 synthesis rate constant is fuzzy uncertain (Triangular MF with 1% perturbation)
μ
Concentration

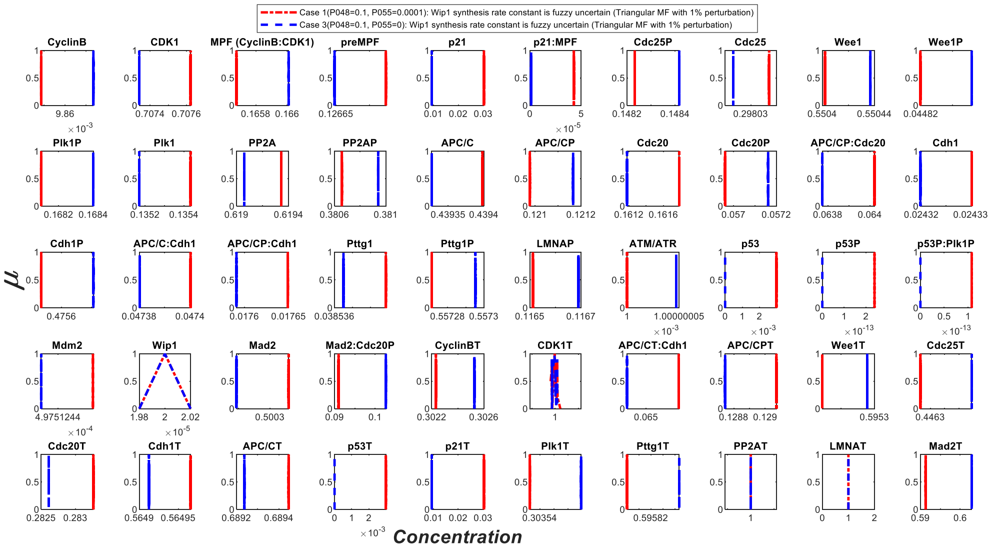
Case 1(P048=0.1, P055=0.0001): Wip1 synthesis rate constant is fuzzy uncertain (Triangular MF with 1% perturbation)
Case 3(P048=0.1, P055=0): Wip1 synthesis rate constant is fuzzy uncertain (Triangular MF with 1% perturbation)
μ
Concentration

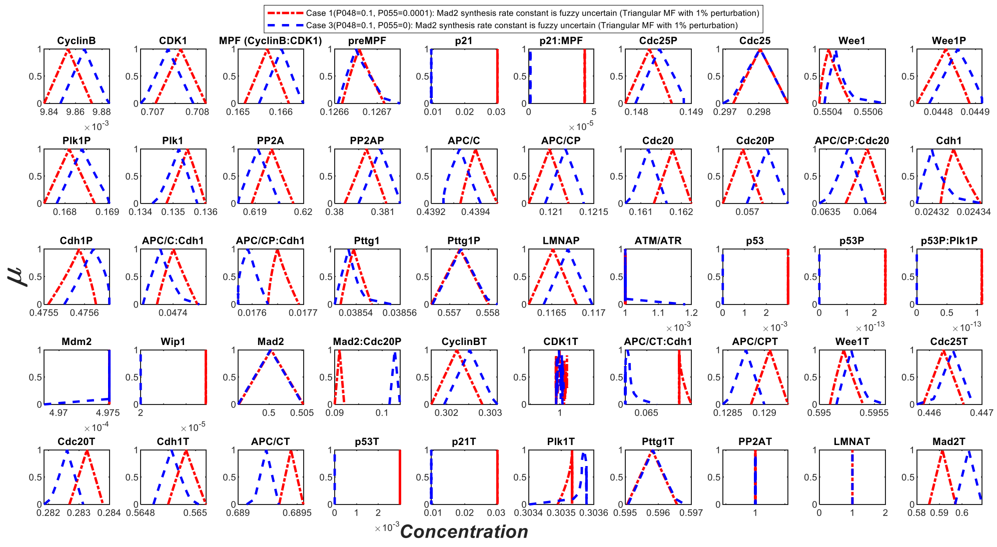

Supplementary Figure 7. Fuzzy analysis for comparison of case 1 (red dot-dashed lines) (p53-wt Plk1-normal) and case 3 (blue dashed lines) (p53-null Plk1-normal). The analysis is performed for 50 model components (shown as titles for panels) by setting k_{s1}, k_{s5}, k_{s8}, k_{s9}, k_{s12}, k_{s13}, k_{s15}, k_{s17}, k_{s20}, k_{s24}, k_{s27}, k_{s28}, k_{s31} k_{s32}, k_{s33} as fuzzy parameters. These parameters control the synthesis of Cyclin B, p21, Cdc25, Wee1, Plk1, PP2A, APC/C, Cdc20, Cdh1, Pttg1, ATM/ATR, p53, Mdm2, Wip1, Mad2 proteins, respectively. Each parameter is perturbed by 1% of its value. The horizontal axis of each panel depicts the maximum uncertainty band of concentration of model components affected by uncertainty of the parameter. The vertical axis shows the different α-cut levels (by increasing the α value from zero, the uncertainty decreases and α = 1, depicts the crisp setting (no uncertainty)).

Supplementary Table 8. Predicted phenotypes of p53-wt and p-53 cancer cells that carry a mutation in the interaction described by the corresponding parameter that is set to 0. "inviable" indicates cell cycle arrest and the number is the ratio of T_m/T, where T_m is the mutant cell cycle period and T is the cell cycle period in wild type.

No	Perturbation	Mutation Description	p53-wt	p53-null	p53-wt (Plk1 =0.055)	p53-null (Plk1 =0.055)
1	$k_{f1} \rightarrow 0$	Loss of association of CycB and CDK1	inviable	inviable	inviable	inviable
2	$k_{r1} \rightarrow 0$	Loss of dissociation of MPF	1.095	1.295	1.382	inviable
3	$k'_{f2} \rightarrow 0$	Loss of dephosphorylation in preMPF by Cdc25P	inviable	inviable	inviable	inviable
4	$k''_{f2} \rightarrow 0$	Loss of dephosphorylation in preMPF by Cdc25	inviable	inviable	inviable	inviable
5	$k'_{r2} \rightarrow 0$	Loss of phosphorylation sites of MPF by Wee1	inviable	inviable	inviable	inviable
6	$k''_{r2} \rightarrow 0$	Loss of phosphorylation sites of MPF by Wee1P	1.006	1.006	1.368	1.839
7	$k'_{f3} \rightarrow 0$	Loss of phosphorylation sites of Cdc25 by MPF	1.335	1.455	inviable	inviable
8	$k''_{f3} \rightarrow 0$	Loss of phosphorylation sites of Cdc25 by Plk1P	1.041	1.043	1.465	1.932
9	$k'_{r3} \rightarrow 0$	Loss of dephosphorylation of Cdc25P by PP2A	0.337	0.335	0.376	0.372
10	$k'_{f4} \rightarrow 0$	Loss of phosphorylation sites of Wee1 by MPF	inviable	inviable	inviable	inviable
11	$k''_{f4} \rightarrow 0$	Loss of phosphorylation sites of Wee1 by Plk1P	1.002	1.002	1.521	1.831
12	$k'_{r4} \rightarrow 0$	Loss of dephosphorylation of Wee1 by PP2A	0.684	0.682	0.756	1.06
13	$k_{f5} \rightarrow 0$	Loss of dissociation of p21:MPF	1.002	1	1.55	1.845
14	$k_{r5} \rightarrow 0$	Loss of association of p21 and MPF	0.998	1	1.351	1.837
15	$k'_{f6} \rightarrow 0$	Loss of phosphorylation sites of Plk1 by MPF	inviable	inviable	inviable	inviable
16	$k'_{r6} \rightarrow 0$	Loss of dephosphorylation of Plk1P by PP2A	inviable	inviable	inviable	inviable
17	$k_{f7} \rightarrow 0$	Loss of dephosphorylation of PP2AP	inviable	inviable	inviable	inviable
18	$k'_{r7} \rightarrow 0$	Loss of phosphorylation sites of PP2A by MPF	0.69	0.688	inviable	inviable
19	$k'_{f8} \rightarrow 0$	Loss of phosphorylation sites of APC/C by MPF	1.056	1.262	1.184	inviable
20	$k''_{f8} \rightarrow 0$	Loss of phosphorylation sites of APC/C by Plk1P	inviable	1.556	1.021	inviable
21	$k'_{r8} \rightarrow 0$	Loss of dephosphorylation of APC/CP by PP2A	inviable	inviable	inviable	inviable
22	$k'_{r9} \rightarrow 0$	Loss of phosphorylation sites of Cdc20 by MPF	0.758	0.76	1.035	1.037
23	$k''_{r9} \rightarrow 0$	Loss of phosphorylation sites of Cdc20 by Plk1P	0.752	0.752	1.184	1.188
24	$k'_{f9} \rightarrow 0$	Loss of dephosphorylation of Cdc20 by PP2A	inviable	inviable	1.822	inviable
25	$k_{f10} \rightarrow 0$	Loss of association of APC/CP and Cdc20	inviable	inviable	inviable	inviable
26	$k_{r10} \rightarrow 0$	Loss of dissociation of APC/CP:Cdc20	inviable	inviable	inviable	inviable

27	$k'_{f11} \to 0$	Loss of dephosphorylation of Cdh1P by PP2A	0.769	0.771	0.853	1.182
28	$k'_{r11} \to 0$	Loss of phosphorylation sites of Cdh1 by MPF	inviable	inviable	inviable	inviable
29	$k''_{r11} \to 0$	Loss of phosphorylation sites of Cdh1 by Plk1P	2.32	2.32	inviable	inviable
30	$k_{f12} \to 0$	Loss of association of APC/C and Cdh1	0.424	0.421	0.467	0.463
31	$k_{r12} \to 0$	Loss of dissociation of APC/C:Cdh1	inviable	inviable	inviable	inviable
32	$k_{f13} \to 0$	Loss of association of APC/CP and Cdh1	0.86	0.944	1.048	1.61
33	$k_{r13} \to 0$	Loss of dissociation of APC/CP:Cdh1	1.64	1.643	inviable	inviable
34	$k'_{f14} \to 0$	Loss of dephosphorylation of Pttg1P by PP2A	1.002	1.004	1.393	1.851
35	$k'_{r14} \to 0$	Loss of phosphorylation sites of Pttg1 by MPF	0.862	0.862	0.977	1.314
36	$k'_{f15} \to 0$	Loss of phosphorylation sites of LMNA by MPF	1	1	1.382	1.837
37	$k_{r15} \to 0$	Loss of dephosphorylation of LMNAP	1	1	1.382	1.837
38	$k'_{f16} \to 0$	Loss of phosphorylation rate constant of p53 by ATM:ATR	1	1	inviable	1.837
39	$k'_{r16} \to 0$	Loss of dephosphorylation rate constant of p53P by Wip1	1	1	1.345	1.837
40	$k_{f17} \to 0$	Loss of association of p53P and Plk1P	1	1	1.407	1.837
41	$k_{r17} \to 0$	Loss of association of p53P: Plk1P	1	1	1.343	1.837
42	$k_{f18} \to 0$	Loss of association of Mad2 and Cdc20P	0.876	0.878	1.18	1.18
43	$k'_{r18} \to 0$	Loss of dissociation of Mad2:Cdc20P	inviable	inviable	inviable	Inviable
44	$k_{s1} \to 0$	Loss of CCNB1 gene	inviable	inviable	inviable	Inviable
45	$k_{s5} \to 0$	Loss of CDKN1A gene	1	1	1.343	1.909
46	$k_{s8} \to 0$	Loss of CDC25 gene	inviable	inviable	inviable	inviable
47	$k_{s9} \to 0$	Loss of WEE1 gene	inviable	inviable	inviable	inviable
48	$k_{s12} \to 0$	Loss of PLK1 gene	inviable	inviable	inviable	inviable
49	$k_{s13} \to 0$	Loss of PP2ACA gene	inviable	inviable	inviable	inviable
50	$k_{s15} \to 0$	Loss of ANAPC/C1 gene	inviable	inviable	inviable	inviable
51	$k_{s17} \to 0$	Loss of CDC20 gene	inviable	inviable	inviable	inviable
52	$k_{s20} \to 0$	Loss of CDH1 gene	0.324	0.322	0.345	inviable
53	$k_{s24} \to 0$	Loss of PTTG1 gene	1.05	1.05	1.626	2.143
54	$k_{s27} \to 0$	Loss of ATM gene	0.998	0.998	1.357	1.357
55	$k_{s28} \to 0$	Loss of TP53 gene	1	1	1.837	1.837
56	$k_{s31} \to 0$	Loss of Mad2 gene	1	1	1.382	1.837
57	$k_{s31p} \to 0$	Loss of p53P-dependent Mdm2 synthesis rate constant	1	1	1.298	1.837
58	$k_{s32} \to 0$	Loss of Wip1 synthesis rate constant	1	1	1.382	1.837
59	$k_{s32p} \to 0$	Loss of p53P-dependent Wip1 synthesis rate constant	1	1	1.655	1.837

60	$k_{s33} \rightarrow 0$	Loss of Mad2 synthesis rate constant	0.876	0.876	1.18	1.18
61	$k_{d1.1} \rightarrow 0$	CycB degron deletion	1.029	1.202	1.159	inviable
62	$k_{d1.2} \rightarrow 0$	Loss of CycB degron site for APC/CP:Cdc20	1.174	1.409	1.424	inviable
63	$k_{d1.3} \rightarrow 0$	Loss of CycB degron site for APC/CT:Cdh1	0.831	0.868	0.901	1.252
64	$k_{d3.1} \rightarrow 0$	MPF degron deletion	1.062	1.225	1.343	inviable
65	$k_{d3.2} \rightarrow 0$	Loss of MPF degron site for APC/CP:Cdc20	inviable	inviable	0.006	inviable
66	$k_{d3.3} \rightarrow 0$	Loss of MPF degron site for APC/CT:Cdh1	0.57	0.847	0.636	inviable
67	$k_{d4.1} \rightarrow 0$	preMPF degron deletion	1.002	1.07	1.264	1.719
68	$k_{d4.2} \rightarrow 0$	Loss of preMPF degron site for APC/CP:Cdc20	1.174	1.411	1.399	inviable
69	$k_{d4.3} \rightarrow 0$	Loss of preMPF degron site for APC/CT:Cdh1	0.758	inviable	0.669	inviable
70	$k_{d5} \rightarrow 0$	p21 degron deletion	0.998	1	1.488	1.783
71	$k_{d6} \rightarrow 0$	Loss of p21 degron site for APC/CP:Cdc20	0.998	1	1.415	1.837
72	$k_{d7.1} \rightarrow 0$	Cdc25P degron deletion	0.696	1.293	1.037	inviable
73	$k_{d7.3} \rightarrow 0$	Loss of Cdc25P degron site for APC/CT:Cdh1	0.992	0.992	1.36	1.81
74	$k_{d8.1} \rightarrow 0$	Cdc25 degron deletion	0.574	0.574	0.643	1.167
75	$k_{d8.3} \rightarrow 0$	Loss of Cdc25 degron site for APC/CT:Cdh1	0.533	0.533	0.57	0.812
76	$k_{d9} \rightarrow 0$	Wee1 degron deletion	inviable	inviable	inviable	inviable
77	$k_{d10} \rightarrow 0$	Wee1P degron deletion	inviable	inviable	inviable	inviable
78	$k_{d11.1} \rightarrow 0$	Plk1P degron deletion	inviable	inviable	inviable	1.719
79	$k_{d11.3} \rightarrow 0$	Loss of Plk1P degron site for APC/CT:Cdh1	0.837	0.839	1.174	1.529
80	$k_{d12.1} \rightarrow 0$	Plk1 degron deletion	inviable	inviable	1.157	1.436
81	$k_{d12.3} \rightarrow 0$	Loss of Plk1 degron site for APC/CT:Cdh1	0.467	0.465	0.634	0.777
82	$k_{d13} \rightarrow 0$	PP2A degron deletion	inviable	inviable	inviable	inviable
83	$k_{d14} \rightarrow 0$	PP2AP degron deletion	1.087	1.087	inviable	inviable
84	$k_{d15} \rightarrow 0$	APC/C degron deletion	inviable	inviable	inviable	inviable
85	$k_{d16} \rightarrow 0$	APC/CP degron deletion	1.256	1.258	1.897	1.93
86	$k_{d17.1} \rightarrow 0$	Cdc20 degron deletion	1.027	1.159	1.911	inviable
87	$k_{d17.3} \rightarrow 0$	Loss of Cdc20 degron site for APC/CT:Cdh1	1.397	1.393	inviable	inviable
88	$k_{d18.1} \rightarrow 0$	Cdc20P degron deletion	0.936	1.207	1.339	inviable
89	$k_{d18.3} \rightarrow 0$	Loss of Cdc20P degron site for APC/CT:Cdh1	0.994	0.998	1.368	1.847
90	$k_{d20} \rightarrow 0$	Cdh1 degron deletion	inviable	inviable	inviable	inviable
91	$k_{d21} \rightarrow 0$	Cdh1P degron deletion	1.095	1.095	1.971	inviable
92	$k_{d24.1} \rightarrow 0$	Pttg1 degron deletion	0.996	0.998	1.372	1.824
93	$k_{d24.2} \rightarrow 0$	Loss of Pttg1 degron site for APC/CP:Cdc20	0.998	1	1.378	1.837
94	$k_{d25.1} \rightarrow 0$	Pttg1P degron deletion	0.998	1	1.38	1.837

95	$k_{d25.2} \to 0$	Loss of Pttg1P degron site for APC/CP:Cdc20	1	1	1.38	1.837
96	$k_{d27} \to 0$	ATM_ATR degron deletion	1.099	inviable	inviable	inviable
97	$k_{d27p} \to 0$	Loss of saturating Wip1-dependent ATM:ATR degradation rate	1	1	1.651	1.837
98	$k_{d28} \to 0$	p53 degron deletion	1.174	1	inviable	1.837
99	$k_{d28p} \to 0$	Loss of Mdm2-dependent p53 degradation rate	1	1	1.362	1.837
100	$k_{d29} \to 0$	Loss of p53P degradation rate constant	1	1	1.357	1.837
101	$k_{d29p} \to 0$	Loss of Mdm2-dependent p53P degradation rate	1	1	1.308	1.837
102	$k_{d31.1} \to 0$	Loss of ATM-ATR dependent Mdm2 inactivation rate	1	1	1.455	1.837
103	$k_{d31} \to 0$	Loss of Mdm2 degradation rate constant	0.998	1	1.355	1.837
104	$k_{d32} \to 0$	Loss of Wip1 degradation rate constant	1	1	1.353	1.829
105	$k_{d33} \to 0$	Loss of Mad2 degradation rate constant	inviable	inviable	inviable	inviable
106	$K_{A1} \to 0$	Loss of Intensifying scale rate of k'_{f6} by ATM_ATR	inviable	inviable	inviable	inviable
107	$K_P \to 0$	Loss of intensifying scale rate of k'_{f11} by Pttg1	0.769	0.771	0.853	1.182
108	$K_{A2} \to 0$	Loss of intensifying scale rate of k_{s8} by ATM_ATR	inviable	inviable	inviable	inviable
109	$K_{p53} \to 0$	Loss of intensifying scale rate of ks5 by p53	inviable	inviable	inviable	inviable
110	$K_{DDS} \to 0$	Loss of intensifying scale rate of DDS	1	1	1.36	1.364
111	$K_{A3} \to 0$	Loss of intensifying scale rate of ks28 by ATM_ATR	inviable	inviable	inviable	inviable
112	$K_{MCD} \to 0$	Loss of intensifying scale rate of Mad2_Cdc20P dissociation by p21	inviable	inviable	inviable	inviable
113	$\varepsilon \to 0$	Loss of intensifying scale of Plk1/p21	1.161	inviable	1.523	inviable
114	$K_{CycB1} \to 0$	Loss of MM constant of CycB degradation by APC/CP:Cdc20	inviable	inviable	inviable	inviable
115	$K_{CycB2} \to 0$	Loss of MM constant of CycB degradation by APC/CP:Cdh1	inviable	inviable	inviable	inviable
116	$K_{MPF1} \to 0$	Loss of MM constant of MPF degradation by APC/CP:Cdc20	inviable	inviable	inviable	inviable
117	$K_{MPF2} \to 0$	Loss of MM constant of MPF degradation by APC/CT:Cdh1	inviable	inviable	inviable	inviable
118	$K_{preMPF1} \to 0$	Loss of MM constant of MPF degradation by APC/CP:Cdc20	inviable	inviable	inviable	inviable
119	$K_{preMPF2} \to 0$	Loss of MM constant of MPF degradation by APC/CP:Cdh1	inviable	inviable	inviable	inviable
120	$K_{p21MPF} \to 0$	Loss of MM constant of $p21_3$:MPF degradation by APC/CP:Cdc20	inviable	inviable	inviable	inviable
121	$K_{Cdc25P1} \to 0$	Loss of MM constant of Cdc25CP Dephosphorylation by PP2A	inviable	inviable	inviable	inviable
122	$K_{Cdc25P2} \to 0$	Loss of MM constant of Cdc25P degradation by APC/CT:Cdh1	inviable	inviable	inviable	inviable
123	$K_{Cdc25} \to 0$	Loss of MM constant of Cdc25 degradation by APC/CT:Cdh1	inviable	inviable	inviable	inviable
124	$K_{Wee1P} \to 0$	Loss of MM constant of Wee1P dephosphorylation by PP2A	inviable	inviable	inviable	inviable

125	$K_{Plk1P1} \rightarrow 0$	Loss of MM constant of Plk1P dephosphorylation by PP2A	inviable	inviable	inviable	inviable
126	$K_{Plk1P2} \rightarrow 0$	Loss of MM constant of Plk1P degradation by APC/CT:Cdh1	inviable	inviable	inviable	inviable
127	$K_{Plk1} \rightarrow 0$	Loss of MM constant of Plk1 degradation by APC/CT:Cdh1	inviable	inviable	inviable	inviable
128	$K_{APC/CP} \rightarrow 0$	Loss of MM constant of APC/CP dephosphorylation by PP2A	inviable	inviable	inviable	inviable
129	$K_{Cdc20P1} \rightarrow 0$	Loss of MM constant of Cdc20P dephosphorylation by PP2A	inviable	inviable	inviable	inviable
130	$K_{Cdc20} \rightarrow 0$	Loss of MM constant of Cdc20 degradation by APC/CT:Cdh1	inviable	inviable	inviable	inviable
131	$K_{Cdc20P2} \rightarrow 0$	Loss of MM constant of Cdc20P degradation by APC/CT:Cdh1	inviable	inviable	inviable	inviable
132	$K_{Cdh1P} \rightarrow 0$	Loss of MM constant of Cdh1P dephosphorylation by PP2A	inviable	inviable	inviable	inviable
133	$K_{Pttg1P1} \rightarrow 0$	Loss of MM constant of Pttg1P dephosphorylation by PP2A	inviable	inviable	inviable	inviable
134	$K_{Pttg1} \rightarrow 0$	Loss of MM constant of Pttg1 degradation by APC/CP:Cdc20	1.002	1.002	1.386	1.839
135	$K_{Pttg1P2} \rightarrow 0$	Loss of MM constant of Pttg1P degradation by APC/CP:Cdc20	inviable	inviable	inviable	inviable
136	$K_{ATMATR} \rightarrow 0$	Loss of MM constant of p53 phosphorylation by Wip1	inviable	inviable	inviable	inviable
137	$K_{Wip1} \rightarrow 0$	Loss of MM constant of ATM_ATR signal degradation by Wip1	inviable	inviable	inviable	inviable

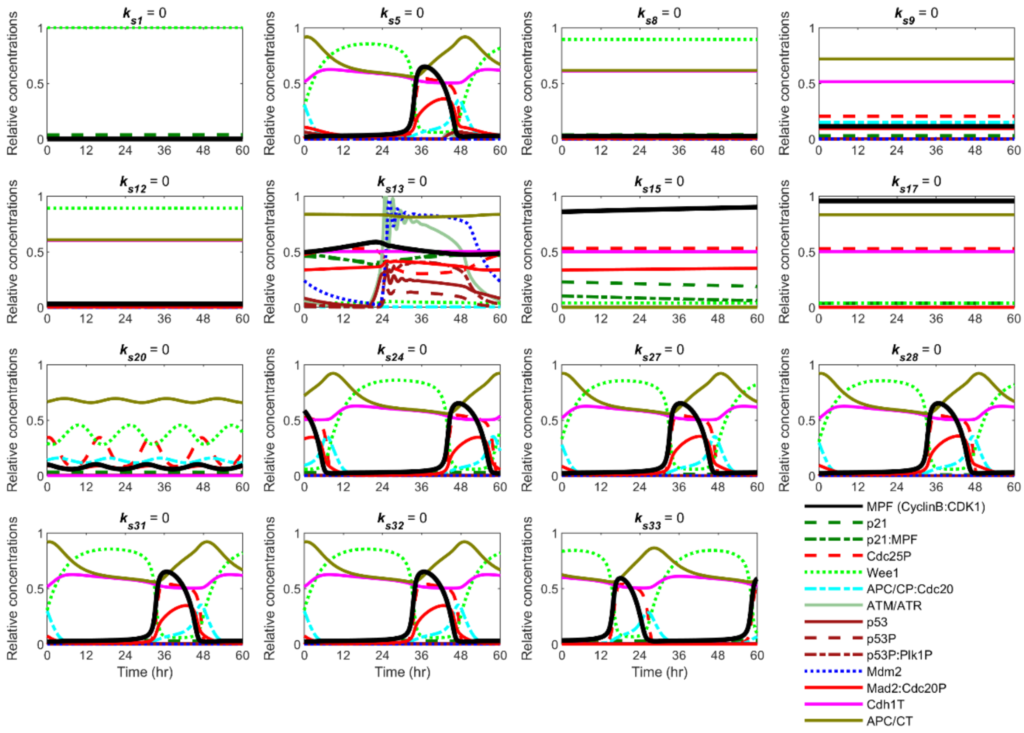

Supplementary Figure 8. Numerical simulations of p53-wt cancer cells with the following gene deletions: CCNB1 and CCNB2 (k_{s1}=0), CDKN1A (k_{s5}=0), CDC25A, CDC25B and CDC25C (k_{s8}=0), WEE1 (k_{s9}=0), PLK1 (k_{s12}=0), PPA2 (k_{s13}=0), ANAPC/C1 (k_{s15}=0), CDC20 (k_{s17}=0), CDH1 (k_{s20}=0), PTTG1 (k_{s24}=0), ATM (k_{s27}=0), TP53 (k_{s28}=0), Mdm2 (k_{s31}=0), Wip1 (k_{s32}=0), MAD2 (k_{s33}=0).

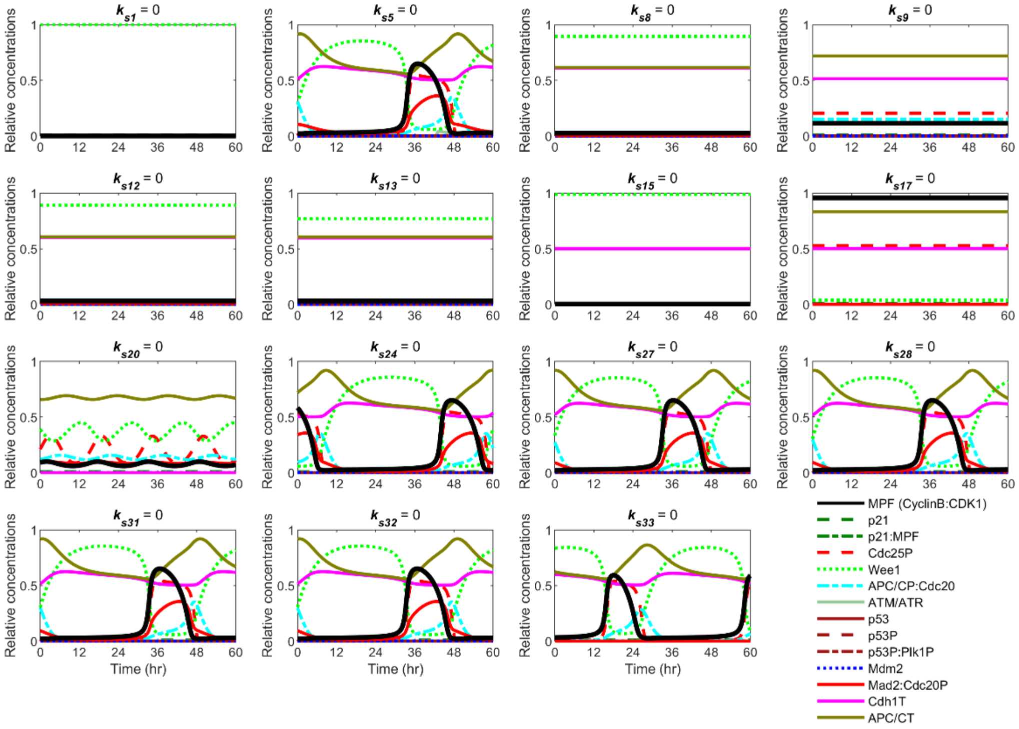

Supplementary Figure 9. Numerical simulations of p53-null cancer cells with the following gene deletions: CCNB1 and CCNB2 (k_{s1}=0), CDKN1A (k_{s5}=0), CDC25A, CDC25B and CDC25C (k_{s8}=0), WEE1 (k_{s9}=0), PLK1 (k_{s12}=0), PPA2 (k_{s13}=0), ANAPC/C1 (k_{s15}=0), CDC20 (k_{s17}=0), CDH1 (k_{s20}=0), PTTG1 (k_{s24}=0), ATM (k_{s27}=0), TP53 (k_{s28}=0), Mdm2 (k_{s31}=0), Wip1 (k_{s32}=0), MAD2 (k_{s33}=0).

Supplementary Table 9. Parameter values used to model cell lines and CRISPR perturbations.

Gene mutated in Cancer Cell Line	Parameter values used to model the corresponding cell line	Perturbed gene	Parameter values used to model the perturbation
CCNB1	$k_{s1} = 0.095$	CCNB1	$k_{s1} = 0.095$
CDKN1A	$k_{s5} = 0$	CDKN1A	$k_{s5} = 0$
CDC25	$k_{s8} = 0.095$	CDC25	$k_{s8} = 0.095$
WEE1	$k_{s9} = 0.0998$	WEE1	$k_{s9} = 0$
PLK1	$k_{s12} = 0.096$	PLK1	$k_{s12} = 0$
PP2ACA	$k_{s13} = 0.098$	PP2ACA	$k_{s13} = 0.062$
APC/C	$k_{s15} = 0.099$	APC/C	$k_{s15} = 0.03$
CDC20	$k_{s17} = 0.0997$	CDC20	$k_{s17} = 0$
FZR1	$k_{s20} = 0$	FZR1	$k_{s20} = 0$
PTTG1	$k_{s24} = 0.05$	PTTG1	$k_{s24} = 0$
ATM and ATR	$k_{s27} = 0$	ATM and ATR	$k_{s27} = 0$
TP53-null	$k_{s28} = 0$	TP53-null	$k_{s28} = 0$
MDM2	$k_{s31} = 0$	MDM2	$k_{s31} = 0$
PPM1D	$k_{s32} = 0.0998$	PPM1D	$k_{s32} = 0$
Mad2L1 or MAD2L2	$k_{s33} = 0.0998$	Mad2L1 or MAD2L2	$k_{s33} = 0$
